# In Vitro and In Silico Evaluation of Anticancer Activity of New Indole-Based 1,3,4-Oxadiazoles as EGFR and COX-2 Inhibitors

**DOI:** 10.3390/molecules25215190

**Published:** 2020-11-07

**Authors:** Belgin Sever, Mehlika Dilek Altıntop, Ahmet Özdemir, Gülşen Akalın Çiftçi, Doha E. Ellakwa, Hiroshi Tateishi, Mohamed O. Radwan, Mahmoud A. A. Ibrahim, Masami Otsuka, Mikako Fujita, Halil I. Ciftci, Taha F. S. Ali

**Affiliations:** 1 Department of Pharmaceutical Chemistry, Faculty of Pharmacy, Anadolu University, Eskişehir 26470, Turkey; mdaltintop@anadolu.edu.tr (M.D.A.); ahmeto@anadolu.edu.tr (A.Ö.); 2 Department of Biochemistry, Faculty of Pharmacy, Anadolu University, Eskişehir 26470, Turkey; gakalin@anadolu.edu.tr; 3 Department of Biochemistry and Molecular Biology, Faculty of Pharmacy, Al-Azhar University, Cairo 11765, Egypt; profdoha@gmail.com; 4 Medicinal and Biological Chemistry Science Farm Joint Research Laboratory, School of Pharmacy, Kumamoto University, Kumamoto 862-0973, Japan; htateishi@kumamoto-u.ac.jp (H.T.); mohamedradwan@kumamoto-u.ac.jp (M.O.R.); motsuka@gpo.kumamoto-u.ac.jp (M.O.); tahafaroukali@gmail.com (T.F.S.A.); 5 Department of Drug Discovery, Science Farm Ltd., Kumamoto 862-0976, Japan; 6 Chemistry of Natural Compounds Department, Pharmaceutical and Drug Industries Research Division, National Research Centre, Dokki, Cairo 12622, Egypt; 7 Computational Chemistry Laboratory, Chemistry Department, Faculty of Science, Minia University, Minia 61519, Egypt; m.ibrahim@compchem.net; 8 Medicinal Chemistry Department, Faculty of Pharmacy, Minia University, Minia 61519, Egypt

**Keywords:** EGFR, COX-2, cancer, indole, oxadiazole, thiazole, benzothiazole, apoptosis, molecular docking

## Abstract

Epidermal growth factor receptor (EGFR) and cyclooxygenase-2 (COX-2) are crucial targetable enzymes in cancer management. Therefore, herein, new 2-[(5-((1*H*-indol-3-yl)methyl)-1,3,4-oxadiazol-2-yl)thio]-*N*-(thiazol/benzothiazol-2-yl)acetamides (**2a–i**) were designed and synthesized as EGFR and COX-2 inhibitors. The cytotoxic effects of compounds **2a**–**i** on HCT116 human colorectal carcinoma, A549 human lung adenocarcinoma, and A375 human melanoma cell lines were determined using MTT assay. 2-[(5-((1*H*-Indol-3-yl)methyl)-1,3,4-oxadiazol-2-yl)thio]-*N*-(6-ethoxybenzothiazol-2-yl)acetamide (**2e**) exhibited the most significant anticancer activity against HCT116, A549, and A375 cell lines with IC_50_ values of 6.43 ± 0.72 μM, 9.62 ± 1.14 μM, and 8.07 ± 1.36 μM, respectively, when compared with erlotinib (IC_50_ = 17.86 ± 3.22 μM, 19.41 ± 2.38 μM, and 23.81 ± 4.17 μM, respectively). Further mechanistic assays demonstrated that compound **2e** enhanced apoptosis (28.35%) in HCT116 cells more significantly than erlotinib (7.42%) and caused notable EGFR inhibition with an IC_50_ value of 2.80 ± 0.52 μM when compared with erlotinib (IC_50_ = 0.04 ± 0.01 μM). However, compound **2e** did not cause any significant COX-2 inhibition, indicating that this compound showed COX-independent anticancer activity. The molecular docking study of compound **2e** emphasized that the benzothiazole ring of this compound occupied the allosteric pocket in the EGFR active site. In conclusion, compound **2e** is a promising EGFR inhibitor that warrants further clinical investigations.

## 1. Introduction

Protein kinases are divided into protein-serine/threonine kinases, protein-tyrosine kinases, and protein-tyrosine kinase-like enzymes related to the origin of the phosphorylated hydroxyl groups. Among them, protein-tyrosine kinases are composed of receptor and non-receptor proteins [1]. Receptor tyrosine kinases (RTKs) lead to the stimulation of crucial downstream signaling pathways related to their structural components, including a single transmembrane helix, which acts as a bridge between an *N*-terminal extracellular ligand-binding domain and a *C*-terminal intracellular tyrosine kinase domain. Ligand-binding to RTKs triggers receptor dimerization, adenosine triphosphate (ATP)-mediated tyrosine autophosphorylation, and initiation of following signal-transduction, which plays a pivotal role in important biological processes such as cell growth, survival, and differentiation [2,3,4]. There are 58 known RTKs in humans, which are classified into several multi-member subfamilies, including receptors of ErbB, insulin, and insulin-like growth factor (IGF-1), platelet-derived growth factor (PDGF), and vascular endothelial growth factor (VEGF) [5,6].

The ErbB subfamily comprises four members, namely epidermal growth factor receptor (EGFR)/HER1/ErbB1, HER2/ErbB2, HER3/ErbB3, and HER4/ErbB4. Although there is no identified ligand for HER2 and no active intrinsic tyrosine kinase domain for HER3, all these members can propagate signal cascades as heterodimers [7,8]. Among these members, EGFR is the most characterized one and has long attracted a great deal of interest. Upon the specific ligands such as EGF and transforming growth factor-α (TGF-α) binding to the extracellular domain of EGFR, homodimerization or heterodimerization with other ErbB family members activates the tyrosine kinase domain and consequent modulation of signal transduction cascades involved in cellular responses including proliferation, migration, adhesion, angiogenesis, and apoptosis [9,10,11,12,13]. Therefore, genetic alterations causing aberrant activity of EGFR have been reported to be implicated in the development of many human cancers, such as lung, colorectal, skin, breast, prostate, kidney, pancreas, ovary, and brain cancers [14,15,16,17].

EGFR is the most detected oncogenic driver in non-small cell lung cancer (NSCLC), which is the most common lung cancer type. A growing body of research focusing on the genetic alterations of EGFR in NSCLC patients has dramatically changed the oncologic management of NSCLC harboring EGFR mutations [18,19,20]. Drugs targeting EGFR have been developed including the use of first-(erlotinib, gefitinib), second-(afatinib, dacomitinib) and third-generation (osimertinib) small-molecule tyrosine kinase inhibitors. Erlotinib is a reversible and competitive ATP inhibitor, which shows its effects by blocking the ATP-binding site within the tyrosine kinase pocket of EGFR [21,22,23].

Colorectal cancer (CRC) is among the most commonly diagnosed cancers and a leading cause of cancer-related mortality across the globe [24]. Overexpression of EGFR has also been reported in CRC along with poor prognosis. EGFR-directed monoclonal antibodies such as cetuximab and panitumumab, which inhibit EGFR activation by binding to the extracellular domain of receptor, are currently recommended therapy options in late-stage CRC without RAS and B-Raf (BRAF) mutations [25,26,27].

Mutations and inadequate regulation of RTKs are also very common alterations in melanoma, which is the most aggressive form of skin cancer [28]. However, the role of EGFR is not completely understood as a regulator or cancer promoter and initiator due to its essential functions in the biology of melanocytes and melanoma [29]. In a study performed by Katunarić et al. [30], high levels of EGFR were found effective on ulceration of nodular melanoma. Wang et al. [31] reported that EGFR was overexpressed in melanoma cells and resistant to proto-oncogene BRAF inhibitors. Several studies also indicated that overexpression of EGFR was correlated with poor prognosis of malignant melanoma [10,32].

Cyclooxygenase-2 (COX-2) and its major metabolite prostaglandin E_2_ (PGE_2_) have also undergone extensive investigation as potential targets of tumor-related angiogenesis, invasion, metastasis, and impairment of apoptosis in a variety of malignancies, including NSCLC and CRC neoplasms. Inducible COX-2 is overexpressed and correlated with cytokines, growth factors, and other stimuli. Celecoxib, as an important COX-2 inhibitor, has been recommended to be included in an active regimen in the treatment of patients with lung cancer. Moreover, insight into the molecular mechanisms underlying cancer, which EGFR and COX-2 interact with, has triggered interest in evaluating the combination of COX-2 and EGFR inhibitors in NSCLC treatment due to the fact that EGFR is activated via PGE_2_ [33,34,35,36,37].

Indole derivatives have been widely used in the management of numerous disorders, including cancer. The indole scaffold is found in natural potent anticancer alkaloids such as vincristine, vinblastine, vinorelbine, and vindesine. Moreover, two marketed RTK inhibitors, sunitinib and osimertinib, possess an indole core. Sunitinib, a PDGFR and VEGFR inhibitor, was approved in the treatment of renal cell carcinoma and gastrointestinal stromal tumor, whereas osimertinib, an EGFR inhibitor, was approved in the treatment of NSCLC [38,39,40]. On the other hand, it was also demonstrated that indomethacin, an indole-based COX-1 and COX-2 inhibitor, exhibited anti-lung cancer and anti-CRC activity through chemical modifications on the main structure [41,42,43,44,45].

1,3,4-Oxadiazole is an important scaffold with its lipophilic nature, its ability to form hydrogen bonds, and its capacity to inhibit different enzymes. Based on these unique features, 1,3,4-oxadiazole-carrying compounds exhibit a broad biological activity spectrum, including anticancer effects. Zibotentan, with a 1,3,4-oxadiazole core, is an important anticancer agent and a lead compound for developing new 1,3,4-oxadiazole-based anticancer agents [46,47,48,49]. In a study performed by El-Sayed et al. [50], novel heterocyclic oxadiazoles were synthesized and evaluated for their anticancer effects as dual selective COX-2 and EGFR inhibitors.

Thiazole and benzothiazole are substantial rings containing electron donating (–S–) and electron-accepting (C=N) groups with multifarious applications, including anticancer activity. Bleomycin, tiazofurin, and dasatinib are thiazole-based agents, whereas phortress is an anticancer agent bearing a benzothiazole core [51,52]. Compounds with thiazole and benzothiazole scaffolds have been determined to possess anticancer effects through COX or EGFR inhibition [53,54,55].

In the light of the aforementioned findings, herein, new compounds containing indole, oxadiazole, and thiazole/benzothiazole cores within an acetamide framework were designed, synthesized, and evaluated for their anticancer effects on NSCLC, CRC, and melanoma cells. The tumor selectivity of the most effective anticancer agent was determined, and this compound was further investigated for its apoptotic influence, and EGFR and COX-2 inhibitory effects to provide a mechanistic insight.

## 2. Results and Discussion

### 2.1. Chemistry

**Scheme 1:**
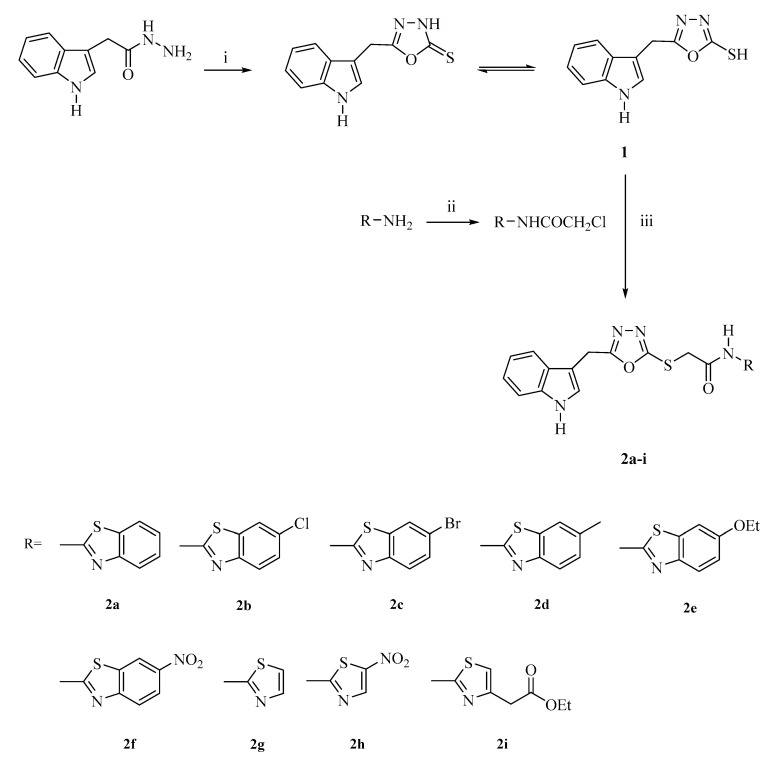
The synthetic route for the preparation of compounds **2a**–**i**. Reagents and conditions: (i) CS_2_, KOH, ethanol, reflux, 6 h; (ii) ClCOCH_2_Cl, TEA, toluene, 0–5 °C; (iii) K_2_CO_3_, acetone, rt, 8 h.

2-[(5-((1*H*-Indol-3-yl)methyl)-1,3,4-oxadiazol-2-yl)thio]-*N*-(thiazol/benzothiazol-2-yl)acetamides (**2a**–**i**) were obtained according to the synthetic route outlined in Scheme 1. Initially, 5-((1*H*-indol-3-yl)methyl)-1,3,4-oxadiazole-2(3*H*)-thione (**1**) was synthesized via the ring closure reaction of indole-3-acetic hydrazide with carbon disulfide in the presence of potassium hydroxide. Then, 2-chloro-*N*-(thiazol/benzothiazol-2-yl)acetamides were synthesized via the reaction of 2-aminothiazoles/benzothiazoles with chloroacetyl chloride in the presence of triethylamine (TEA). In the last step, the nucleophilic substitution reaction of 5-((1*H*-indol-3-yl)methyl)-1,3,4-oxadiazole-2(3*H*)-thione with 2-chloro-*N*-(thiazol/benzothiazol-2-yl)acetamides in the presence of potassium carbonate afforded the target compounds (**2a**–**i**). The structures of the synthesized compounds were verified by infrared (IR), ^1^H nuclear magnetic resonance (NMR), ^13^C-NMR, and high resolution mass spectrometry (HRMS).

In the IR spectra of compounds **2a**–**i**, the N–H stretching vibrations belonging to the indole rings and acetamide moieties gave rise to the bands at 3462.22–3363.86 cm^−1^. Moreover, the aromatic and aliphatic C–H stretching vibrations appeared at 3190.26–3043.67 cm^−1^ and 2962.66–2709.99 cm^−1^, respectively. The amide C=O stretching bands occurring at 1687.71–1664.57 cm^−1^ confirmed the formation of the final compounds. In the IR spectrum of compound **2i**, the ester C=O stretching band was also observed at 1718.58 cm^−1^. The C=N, C=C stretching, and N–H bending bands were determined at 1614.42–1456.26 cm^−1^.

In the ^1^H-NMR spectra of compounds **2a**–**i**, the characteristic singlet peaks at 4.32–4.40 ppm and 12.48–13.47 ppm belonging to S-CH_2_ and NH protons, respectively, confirmed the formation of the thioacetamide moieties of final compounds. Along with this data, CH_2_ and NH protons of the (indol-3-yl)methyl group appeared as singlets at 4.28–4.33 ppm and 11.02–11.05 ppm, respectively. The CH_3_ protons of compound **2d** were detected as a singlet at 2.41 ppm, whereas the CH_3_ protons of compounds **2e** and **2i** were detected as triplets at 1.34 ppm (*J* = 6.93, 6.96, 13.89 Hz) and 1.18 ppm (*J* = 7.11, 7.08, 14.19 Hz), respectively. Moreover, the CH_2_ protons of the ethyl groups of compounds **2e** and **2i** were observed as quartets at 4.06 ppm (*J* = 6.93, 13.92 Hz) and 4.08 ppm (*J* = 7.11, 14.22 Hz), respectively, while the CH_2_ protons of the acetate group of compound **2i** were determined as a singlet at 3.69 ppm. In the ^13^C-NMR spectra of compounds **2a**–**i**, the prominent peaks at 37.09–35.76 ppm and 167.76–168.50 ppm belonging to S-CH_2_ and C=O carbons, respectively also confirmed the formation of the thioacetamide moieties of final compounds. The CH_2_ carbon of the (indol-3-yl)methyl group appeared at 21.88–21.90 ppm. The CH_3_ carbon of compounds **2d**, **2e**, and **2i** was determined at 21.45, 21.90, and 14.56 ppm, respectively, and the CH_2_ carbon of compounds **2e** and **2i** came in sight at 64.07 and 60.80 ppm, respectively. Additionally, the CH_2_ and carbonyl carbons of the acetate group of compound **2i** emanated at 35.74 and 170.45 ppm, respectively. The carbons of the oxadiazole ring became apparent at 162.80–167.75 ppm. All the aromatic protons and carbons belonging to the indole, benzothiazole, and thiazole scaffolds were consistent with the proposed structures of the compounds. Finally, the HRMS data of all compounds were coherent with their molecular formulas.

### 2.2. Cytotoxicity

MTT assay was carried out to determine the cytotoxic effects of compounds **2a**–**i** on HCT116 human colorectal carcinoma, A549 human lung adenocarcinoma, and A375 human melanoma cell lines. Compound **2e** was identified as the most effective compound on HCT116, A549, and A375 cell lines with IC_50_ values of 6.43 ± 0.72 μM, 9.62 ± 1.14 μM, and 8.07 ± 1.36 μM, respectively, when compared with erlotinib (IC_50_ = 17.86 ± 3.22 μM, 19.41 ± 2.38 μM, and 23.81 ± 4.17 μM, respectively) (Table 1). This outcome pointed out that compound **2e** showed higher cytotoxicity than erlotinib on these three cell lines, and 6-ethoxybenzothiazole moiety of this compound remarkably increased the anticancer activity. On the contrary, the introduction of the nitro substituent into the 6th position of benzothiazole scaffold significantly diminished anticancer efficacy as observed in the comparison of compounds **2a** and **2f.** Moreover, thiazole substitution caused a decrease in anticancer activity as shown in compounds **2g**, **2h**, and **2i**. Following compound **2e**, 6-methylbenzothiazole-substituted compound **2d** and benzothiazole-substituted compound **2a** led to an increase in cytotoxic activity towards these three cells, particularly the HCT-116 cell line, with IC_50_ values of 18.23 ± 1.19 μM and 26.25 ± 1.75 μM, respectively. Therefore, the tumor selectivity of compounds **2a**, **2d**, and **2e** was also investigated between Jurkat human leukemic T-cells and peripheral blood mononuclear cells (PBMCs). These compounds exhibited notable tumor selectivity, whereas compound **2e** was found as the most selective anticancer agent (IC_50_ = 6.45 ± 1.02 μM for Jurkat cells, IC_50_ > 300 μM for PBMCs; selectivity index (SI) > 46.51) compared to erlotinib (Table 2). These results show that compound **2e** has a high safety profile on PBMCs and exhibits better potency and SI than erlotinib against the Jurkat cell line.

### 2.3. Apoptosis

**Figure 1:**
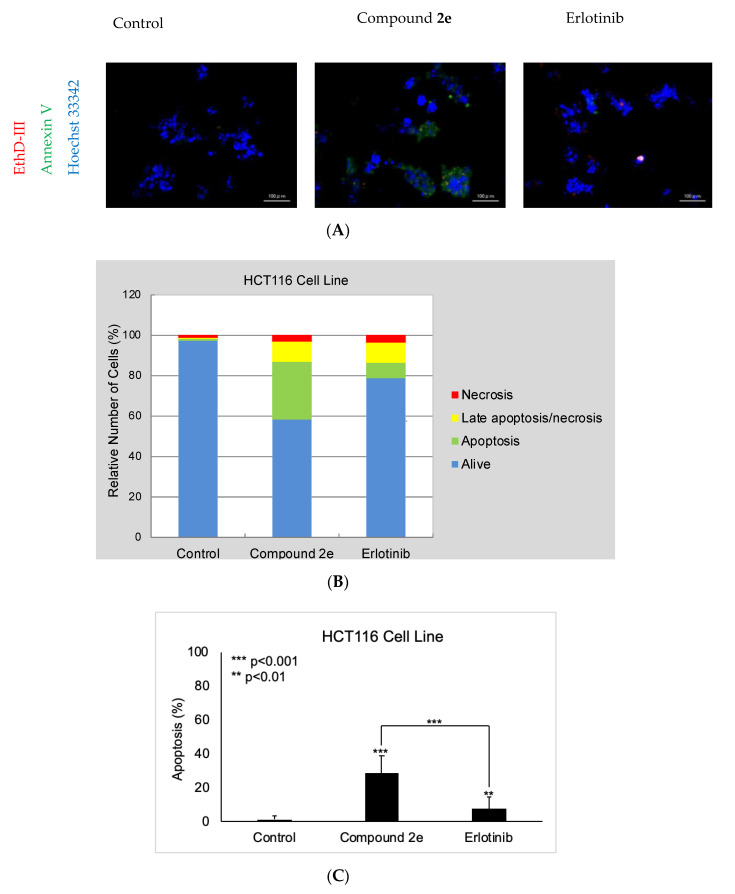
The apoptosis-inducing effects of compound **2e** and erlotinib on the HCT116 cell line. Alteration in HCT116 cells at 10 μM concentration of control, compound **2e**, and erlotinib (**A**) for 12 h. (**B**) A total of approximately 100 stained cells were selected randomly in each experiment of (**A**) and were classified into 4 types: “alive” (blue), “apoptosis” (green), “necrosis or late apoptosis” (both green and red), and “necrosis” (red). (**C**) Quantification of the effect of compound **2e** and erlotinib on apoptosis. Data from three independent experiments are shown as means ± standard deviations and *p* values were determined using Student’s test.

Due to the significant anticancer potential of compound **2e** on the HCT116 cell line, it was further screened for the determination of its apoptotic activity in the HCT116 cell line using the Hoechst 33342/annexin V/ethidium homodimer III staining method (Figure 1A). According to the results, compound **2e** induced apoptosis (28.35%) in HCT116 cells more significantly than erlotinib (7.42%), while late apoptotic or necrotic effects of compound **2e** and erlotinib were determined to be 9.81% and 10.45%, respectively (Figure 1B,C).

### 2.4. Kinase and COX Inhibition

The pivotal role of RTKs in the pathogenesis of cancer prompted us to investigate the inhibitory effects of compound **2e** on eight members of RTKs (EGFR, HER2, HER4, IGF1R, insulin receptor (InsR), kinase insert domain receptor (KDR), and PDGF receptors (PDGFRα and PDGFRβ)) using the kinase profiling assay protocol as previously described [6]. The results indicated that compound **2e** significantly inhibited EGFR with an IC_50_ value of 2.80 ± 0.52 μM when compared with erlotinib (IC_50_ = 0.04 ± 0.01 μM), which shed light on its striking anticancer effects. Moreover, compound **2e** also inhibited HER4 and PDGFRβ with IC_50_ values of 8.22 ± 1.64 μM and 6.16 ± 1.58 μM, respectively. This outcome demonstrated that compound **2e** predominantly inhibited EGFR, the most crucial target in this large panel of kinases (Table 3).

Compound **2e** was also screened for its COX-1 and COX-2 inhibitory potencies. According to the results, compound **2e** displayed weak but selective COX-2 inhibition with an IC_50_ value of 37.5 ± 3.54 μM (SI = 1.96) compared to celecoxib (IC_50_ = 2.75 ± 0.05 μM; SI = 3.23) and indomethacin (IC_50_ = 0.575 ± 0.075 μM; SI = 0.21). These results remarked that compound **2e** showed its anticancer effects via EGFR inhibition and COX-independent action (Table 4).

### 2.5. Molecular Docking

In silico molecular docking simulation was accomplished to rationalize the significant EGFR inhibitory effect of compound **2e**. It was reported that erlotinib binds to both the active and inactive conformations of EGFR. In contrast, lapatinib selectively binds to the inactive kinase due to its bulky [(3-fluorobenzyl)oxy] moiety on its aniline ring. This bulky group cannot bind to the narrow space of the ATP-binding pocket. On the contrary, it can project into the allosteric site opened up upon dislocation of the αC helix in the inactive conformation. For compound **2e**, similarly, its bulky benzothiazole ring cannot be adapted in the narrow space of the ATP-binding site. Therefore, it was clear that the large molecular size of compound **2e** allows it to selectively bind to the inactive conformation of EGFR but not to the active configuration. Subsequently, compound **2e** was docked in the inactive conformation of EGFR (PDB code: 4HJO) to elucidate the structural mechanism of EGFR inhibition by this new class of compounds [56]. It can be considered that compound **2e** is an ATP-competitive EGFR inhibitor like erlotinib, although its binding affinity is lower than that of erlotinib [57].

**Figure 2:**
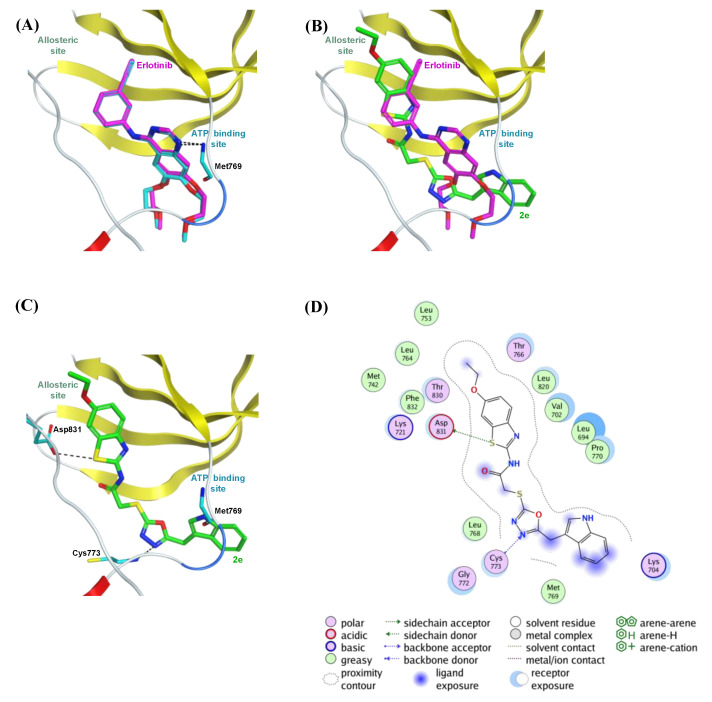
The top-scoring docked pose of compound **2e** to the EGFR active site (PDB code 4HJO) as predicted by MOE 2019.01. (**A**) Comparison of modeled binding mode of the co-crystallized ligand erlotinib (magenta sticks) and its superimposed docking conformation (cyan sticks). (**B**) Comparison of modeled binding mode of compound **2e** (green sticks) and erlotinib (magenta sticks). (**C**) Detailed binding mode of compound **2e** (green sticks) displaying hydrogen bonds (black dashed line) with the critical amino acid residues (cyan sticks). (**D**) Two-dimensional depiction of compound **2e** binding interactions with the essential amino acid residues.

The co-crystallized ligand erlotinib was initially re-docked in its corresponding co-crystal structure (PDB code: 4HJO) to inspect whether MOE is capable of precisely replicating the correct binding mode of the co-crystallized inhibitor. Figure 2A illustrates the superimposition of the co-crystallized ligand erlotinib and its superimposed docking conformation, where erlotinib was correctly docked into its crystal structure with an RMSD value 0.3 Å, and it showed H-bond interaction to the essential amino acid residue (Met769), similar to the co-crystallized ligand.

As shown in Figure 2B, the top-ranked pose for compound **2e** was incorporated into the EGFR active site, where its bulky 6-ethoxybenzothiazole moiety was oriented to the allosteric space. Moreover, binding free energies of the top-ranked docking poses for re-docked erlotinib and compound **2e** were −9.01 and −8.34 kcal/mol, respectively. These binding affinities are well associated with the in vitro EGFR inhibition assay (Table 3), where erlotinib inhibits the EGFR more potently than compound **2e**.

Figure 2C illustrates the detailed binding of compound **2e** within the EGFR active site. The benzothiazole sulfur atom made H-bond interaction with the amino acid residue Asp831. Moreover, one nitrogen atom of the oxadiazole ring showed H-bond interaction with the amino acid residue Cys773. The 2D depiction of compound **2e** binding to the critical amino acid residues (Figure 2D) highlighted the importance of 6-ethoxy substituent of benzothiazole moiety. This substituent was deeply embedded in the allosteric space, where it increased the binding affinity of compound **2e** through hydrophobic contacts with the surrounding hydrophobic amino acid residues (Ala719, Leu753, and Leu764). These hydrophobic and hydrophilic binding interactions may be accountable for the EGFR inhibition potency of compound **2e**, although the lack of the interactions between the compound **2e** and the crucial amino acid residue (Met769) in the ATP binding pocket could rationalize the lower EGFR inhibitory activity of compound **2e** compared to erlotinib. Despite further structural modifications required to enhance compound **2e** potency via binding to the critical amino acid Met769, these docking results demonstrate that compound **2e** is a promising EGFR inhibitor.

**Figure 3:**
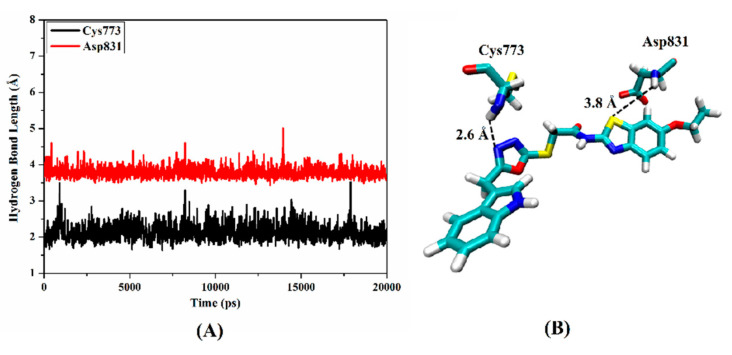
(**A**) Hydrogen bond lengths (in **A**) between compound **2e** and the key amino residues (Cys773 and Asp831) of EGFR over 20 ns MD simulation. (**B**) Three-dimensional representation of the average structure of compound **2e** inside the active site of EGFR during 20 ns of MD simulation.

Molecular dynamics (MD) simulation over 20 ns was performed on compound **2e** in complex with EGFR to obtain a more in-depth insight into the stability of compound **2e** inside the active site. The hydrogen bond lengths between compound **2e** and the key amino acids (Cys773 and Asp831) were investigated throughout the MD course (Figure 3). From the data in Figure 3, it is apparent that compound **2e** demonstrated a stable hydrogen bond with Cys773 with an average bond length of 2.6 A and showed a persistent 97.6% of the production MD trajectory snapshots. However, the average hydrogen bond length between compound **2e** and Asp831 was 3.8 Å, demonstrating such interaction’s instability.

**Figure 4:**
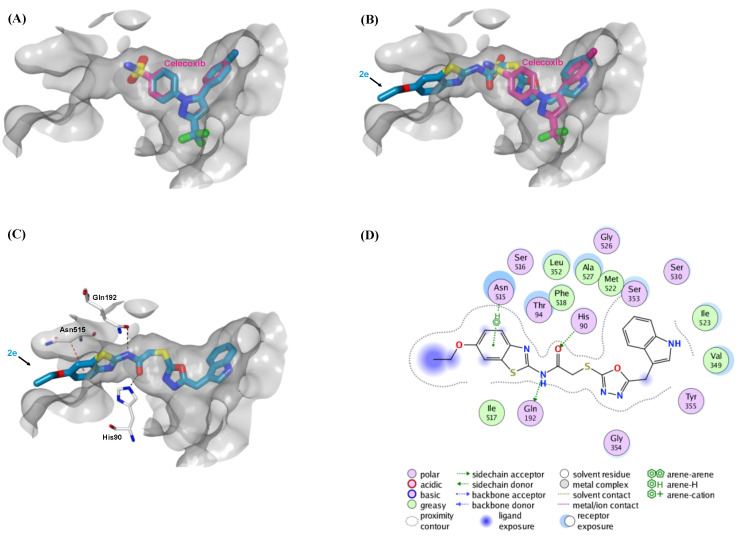
The top-scoring docked pose of compound **2e** bound to the active site of COX-1 (PDB code 3KK6). The active site is displayed in surface view (light grey). (**A**) Comparison of modeled binding mode of the co-crystallized ligand celecoxib (magenta sticks) and its superimposed docking conformation (cyan sticks). (**B**) Comparison of modeled binding mode of compound **2e** (cyan sticks) and celecoxib (magenta sticks). (**C**) Detailed binding mode of compound **2e** (cyan sticks) displaying hydrogen bonds (black dashed line) and CH–π interaction (red dashed line) with the critical amino acid residues (white sticks). (**D**) Two-dimensional depiction of compound **2e** binding interactions with the essential amino acid residues.

**Figure 5:**
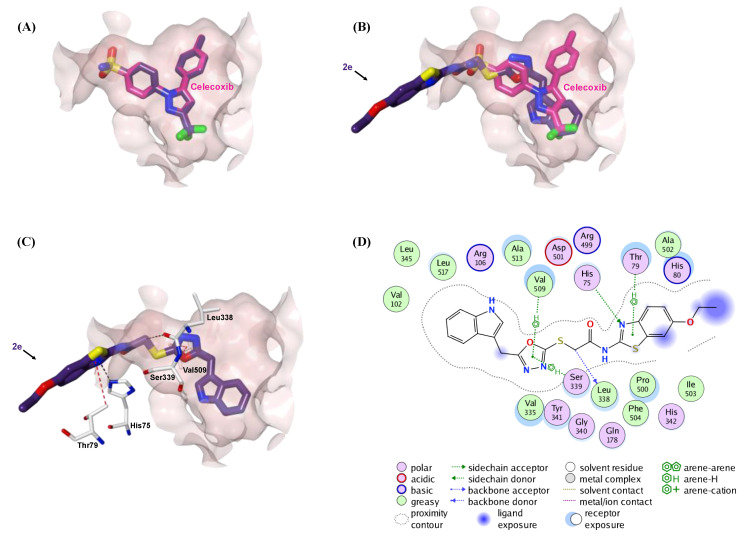
The top-scoring docked pose of compound **2e** bound to the active site of COX-2 (PDB code 3LN1). The active site is displayed in the surface view (light pink). (**A**) Comparison of modeled binding mode of the co-crystallized ligand celecoxib (magenta sticks) and its superimposed docking conformation (violet sticks). (**B**) Comparison of modeled binding mode of compound **2e** (violet sticks) and celecoxib (magenta sticks). (**C**) Detailed binding mode of compound **2e** (violet sticks) displaying hydrogen bonds (black dashed line) and CH–π interaction (red dashed line) with the critical amino acid residues (white sticks). (**D**) Two-dimensional depiction of compound **2e** binding interactions with the essential amino acid residues.

Before docking simulation of compound **2e** to COX enzymes, the co-crystallized ligand celecoxib was initially re-docked in both COX X-ray structures (PDB codes: 3KK6 and 3LN1) to inspect whether MOE is capable of precisely replicating the correct binding mode of the co-crystallized inhibitor. Figure 4A and Figure 5A illustrated the superimposition of the co-crystallized ligand celecoxib and its superimposed docking conformation, where celecoxib was correctly docked into its co-crystallized COX-1 and COX-2 structures with RMSD values of 0.90 Å and 0.86 Å, respectively.

Compared to the large EGFR active site, the active sites of both COX enzymes are narrower to accommodate larger molecules such as compound **2e**. Therefore, compound **2e** is expected to be partially embedded in the binding sites of both COX enzymes and thus has weak inhibitory effects on these enzymes. As illustrated in Figure 4B and Figure 5B, the binding mode of compound **2e** revealed that its bulky 6-ethoxybenzothiazole moiety oriented towards the solvent-exposed area outside the active site. Moreover, the oxadiazole ring of compound **2e** formed two CH–π interactions with two residues (Ser339 and Val509) in the COX-2 active site, whereas this ring was unable to make any interactions in COX-1 (Figure 4C,D and Figure 5C,D). These extra binding interactions with COX-2 could explain why compound **2e** is more selective to COX-2 compared to COX-1. As anticipated, the binding free energies of top-ranked docking poses for compound **2e** to COX-1 and COX-2 proteins were −8.3 and −8.8 kcal/mol, respectively, which are lower than those for celecoxib (−9.7 and −10.1 kcal/mol, respectively). These relative binding affinities are well associated with the in vitro COX inhibition assay (Table 4), where celecoxib inhibits the COX enzyme more potently than does compound **2e** by more than 10-fold.

To sum up, our in silico study discloses the role of the benzothiazole ring to the EGFR inhibitory mechanism of compound **2e** mainly through binding to the allosteric site. However, the bulkiness of this ring prevents the strong binding of compound **2e** to COX enzymes. Consequently, the antiproliferative activity of compound **2e** cannot be attributed to its COX inhibitory activity, but due to its EGFR inhibition.

## 3. Materials and Methods

### 3.1. Chemistry

All reagents purchased from commercial suppliers were used without additional purification. The melting points (mps) of the compounds were determined on an MP90 digital melting point apparatus (Mettler, Toledo, OH, USA) and are uncorrected. IR spectra were recorded on an IRPrestige-21 Fourier transform infrared spectrophotometer (Shimadzu, Tokyo, Japan). ^1^H-NMR and ^13^C-NMR spectra were recorded on a Bruker spectrometer (Bruker, Billerica, MA, USA), while HRMS spectra were recorded on a Shimadzu LCMS–IT–TOF system (Shimadzu, Kyoto, Japan). Thin layer chromatography (TLC) was performed on TLC Silica gel 60 F_254_ aluminum sheets (Merck, Darmstadt, Germany) to control the purity of the compounds.

#### General Procedure for the Synthesis of the Compounds

##### 5-((1*H*-Indol-3-yl)methyl)-1,3,4-oxadiazole-2(3*H*)-thione (**1**)

A mixture of indole-3-acetic hydrazide (0.025 mol) and carbon disulfide (0.03 mol) in the presence of potassium hydroxide (0.025 mol) in ethanol (50 mL) was refluxed for 6 h. The solution was cooled and acidified with hydrochloric acid solution. The solid was filtered off, washed with water, and dried. The product was crystallized from ethanol [47].

##### 2-Chloro-*N*-(aryl)acetamides

Chloroacetyl chloride (0.03 mol) was added dropwise to a mixture of aromatic amine (0.025 mol) and TEA (0.025 mol) in toluene (50 mL) at 0–5 °C. The solvent was evaporated under reduced pressure. The residue was washed with water to remove TEA and crystallized from ethanol. The products were crystallized from ethanol [47].

##### 2-[(5-((1*H*-Indol-3-yl)methyl)-1,3,4-oxadiazol-2-yl)thio]-*N*-(thiazol/benzothiazol-2-yl)acetamides (**2a**–**i**)

A mixture of compound **1** (0.0015 mol) and appropriate 2-chloro-*N*-(aryl)acetamide (0.0015 mol) in acetone (25 mL) was stirred at room temperature for 8 h in the presence of potassium carbonate (0.0015 mol). The solvent was evaporated under reduced pressure. The residue was washed with water and crystallized from ethanol [47].

##### 2-[(5-((1*H*-Indol-3-yl)methyl)-1,3,4-oxadiazol-2-yl)thio]-*N*-(benzothiazol-2-yl)acetamide (**2a**)

Mp: 200.9–201.7 °C. Yield: 80%. IR ν_max_ (cm^−1^): 3450.65, 3429.43 (N–H stretching), 3170.97, 3136.25, 3057.17 (aromatic C–H stretching), 2962.66, 2920.23, 2848.86, 2735.06 (aliphatic C–H stretching), 1681.93 (amide C=O stretching), 1604.77, 1566.20, 1560.41, 1489.05 (N–H bending, C=N and C=C stretching), 1438.90, 1382.96, 1332.81, 1332.81, 1261.45, 1236.37, 1161.15, 1089.78, 1012.63 (C–H bending, C–N and C–O stretching, aromatic C–H in plane bending), 995.27, 935.48, 867.97, 810.10, 750.31, 671.23 (aromatic C–H out of plane bending and C–S stretching) (Supplementary Material Figure S1). ^1^H-NMR (300 MHz, DMSO-*d*_6_): 4.33 (s, 2H), 4.39 (s, 2H), 6.98 (t, *J* = 7.47, 7.37, 14.84 Hz, 1H), 7.08 (t, *J* = 7.46, 7.59, 15.05 Hz, 1H), 7.30–7.38 (m, 3H), 7.43–7.51 (m, 2H), 7.78 (d, *J* = 7.98 Hz, 1H), 7.99 (d, *J* = 7.59 Hz, 1H), 11.05 (s, 1H), 12.74 (s, 1H) (Supplementary Material Figure S2). ^13^C-NMR (100 MHz, DMSO-*d*_6_): 21.90 (CH_2_), 36.06 (CH_2_), 106.97 (C), 112.05 (CH), 118.61 (CH), 119.24 (CH), 121.18 (CH), 121.78 (CH), 122.25 (CH), 124.21 (CH), 124.66 (CH), 126.69 (CH), 127.03 (C), 131.93 (C), 136.62 (C), 148.97 (C), 158.09 (C), 163.00 (C), 166.67 (C), 167.82 (C) (Supplementary Material Figure S3). HRMS (ESI) (*m/z*) [M + H]^+^ calcd. for C_20_H_15_N_5_O_2_S_2_: 422.0740, found: 422.0737 (Supplementary Material Figure S4).

##### 2-[(5-((1*H*-Indol-3-yl)methyl)-1,3,4-oxadiazol-2-yl)thio]-*N*-(6-chlorobenzothiazol-2-yl)acetamide (**2b**)

Mp: 241.2–242.0 °C. Yield: 86%. IR ν_max_ (cm^−1^): 3462.22, 3433.29 (N–H stretching), 3174.83, 3062.96 (aromatic C–H stretching), 2916.37, 2819.93, 2727.35 (aliphatic C–H stretching), 1681.93 (amide C=O stretching), 1606.70, 1571.99, 1487.12 (N–H bending, C=N and C=C stretching), 1440.83, 1419.61, 1375.25, 1336.67, 1267.23, 1234.44, 1166.93, 1097.50, 1051.20, 1014.56 (C–H bending, C–N and C–O stretching, aromatic C–H in plane bending), 979.84, 860.25, 815.89, 727.16, 688.59 (aromatic C–H out of plane bending and C–S stretching) (Supplementary Material Figure S5). ^1^H-NMR (300 MHz, DMSO-*d*_6_): 4.33 (s, 2H), 4.40 (s, 2H), 6.98 (t, *J* = 7.47, 14.94 Hz, 1H), 7.06 (t, *J* = 7.62, 15.24 Hz, 1H), 7.35 (t, *J* = 8.10, 6.48, 14.58 Hz, 2H), 7.48 (t, *J* = 6.69, 6.33, 13.02 Hz, 2H), 7.77 (d, *J* = 8.61 Hz, 1H), 8.14 (brs, 1H), 11.05 (s, 1H), 12.84 (s, 1H) (Supplementary Material Figure S6). ^13^C-NMR (100 MHz, DMSO-*d*_6_): 21.89 (CH_2_), 36.01 (CH_2_), 106.96 (C), 112.05 (CH), 118.60 (CH), 119.24 (2CH), 121.78 (CH), 121.98 (CH), 122.40 (CH), 124.65 (CH), 127.04 (C), 128.27 (C), 133.63 (C), 136.62 (C), 147.87 (C), 158.95 (C), 162.95 (C), 167.00 (C), 167.83 (C) (Supplementary Material Figure S7). HRMS (ESI) (*m/z*) [M + H]^+^ calcd. for C_20_H_14_ClN_5_O_2_S_2_: 456.0350, found: 456.0351(Supplementary Material Figure S8).

##### 2-[(5-((1*H*-Indol-3-yl)methyl)-1,3,4-oxadiazol-2-yl)thio]-*N*-(6-bromobenzothiazol-2-yl)acetamide (**2c**)

Mp: 240.6–241.5 °C. Yield: 82%. IR ν_max_ (cm^−1^): 3454.51 (N–H stretching), 3170.97, 3078.39 (aromatic C–H stretching), 2960.73, 2843.07, 2723.49 (aliphatic C–H stretching), 1681.93 (amide C=O stretching), 1604.77, 1568.13, 1487.12 (N–H bending, C=N and C=C stretching), 1436.97, 1375.25, 1334.74, 1267.23, 1234.44, 1165.00, 1083.99 (C–H bending, C–N and C–O stretching, aromatic C–H in plane bending), 991.41, 854.47, 812.03, 775.38, 744.52, 729.09, 682.80, 650.01 (aromatic C–H out of plane bending and C–S stretching) (Supplementary Material Figure S9). ^1^H-NMR (300 MHz, DMSO-*d*_6_): 4.33 (s, 2H), 4.39 (s, 2H), 6.98 (t, *J* = 7.11, 7.08, 14.19 Hz, 1H), 7.08 (t, *J* = 7.02, 7.59, 14.61 Hz, 1H), 7.32–7.37 (m, 2H), 7.49 (d, *J* = 7.83 Hz, 1H), 7.59 (d, *J* = 8.60 Hz, 1H), 7.71 (d, *J* = 8.61 Hz, 1H), 8.26 (brs, 1H), 11.04 (s, 1H), 12.83 (s, 1H) (Supplementary Material Figure S10). ^13^C-NMR (100 MHz, DMSO-*d*_6_): 21.89 (CH_2_), 36.01 (CH_2_), 106.96 (C), 112.04 (CH), 116.16 (C), 118.60 (CH), 119.24 (CH), 121.78 (CH), 122.81 (CH), 124.65 (CH), 124.82 (CH), 127.02 (CH), 129.73 (C), 134.13 (C), 136.62 (C), 148.16 (C), 158.91 (C), 162.95 (C), 167.01 (C), 167.83 (C) (Supplementary Material Figure S11). HRMS (ESI) (*m/z*) [M + H]^+^ calcd. for C_20_H_14_BrN_5_O_2_S_2_: 499.9845, found: 499.9864(Supplementary Material Figure S12).

##### 2-[(5-((1*H*-Indol-3-yl)methyl)-1,3,4-oxadiazol-2-yl)thio]-*N*-(6-methylbenzothiazol-2-yl)acetamide (**2d**)

Mp: 204.6–205.3 °C. Yield: 79%. IR ν_max_ (cm^−1^): 3429.43, 3408.22 (N-H stretching), 3159.40, 3055.24 (aromatic C–H stretching), 2960.73, 2864.29 (aliphatic C–H stretching), 1680.00 (amide C=O stretching), 1610.56, 1587.42, 1556.55, 1487.12, 1456.26 (N–H bending, C=N and C=C stretching), 1421.54, 1338.60, 1265.30, 1155.36, 1087.85 (C–H bending, C–N and C–O stretching, aromatic C–H in plane bending), 987.55, 889.18, 813.96, 742.59, 684.73, 653.87 (aromatic C–H out of plane bending and C–S stretching) (Supplementary Material Figure S13). ^1^H-NMR (300 MHz, DMSO-*d*_6_): 2.41 (s, 3H), 4.32 (s, 2H), 4.37 (s, 2H), 6.97 (t, *J* = 7.86, 7.02, 14.88 Hz, 1H), 7.08 (t, *J* = 7.02, 8.07, 15.09 Hz, 1H), 7.26 (d, *J* = 8.28 Hz, 1H), 7.32–7.37 (m, 2H), 7.49 (d, *J* = 7.83 Hz, 1H), 7.65 (d, *J* = 8.25 Hz, 1H), 7.76 (brs, 1H), 11.04 (s, 1H), 12.65 (s, 1H) (Supplementary Material Figure S14). ^13^C-NMR (100 MHz, DMSO-*d*_6_): 21.45 (CH_3_), 21.89 (CH_2_), 36.04 (CH_2_), 106.96 (C), 112.05 (CH), 118.61 (CH), 119.24 (2CH), 120.82 (CH), 121.78 (CH), 124.65 (2CH), 127.03 (C), 128.01 (C), 133.70 (C), 136.62 (C), 146.93 (C), 157.19 (C), 162.99 (C), 166.60 (C), 167.81 (C) (Supplementary Material Figure S15). HRMS (ESI) (*m/z*) [M + H]^+^ calcd. for C_21_H_17_N_5_O_2_S_2_: 436.0896, found: 436.0893(Supplementary Material Figure S16).

##### 2-[(5-((1*H*-Indol-3-yl)methyl)-1,3,4-oxadiazol-2-yl)thio]-*N*-(6-ethoxybenzothiazol-2-yl)acetamide (**2e**)

Mp: 201.6–202.8 °C. Yield: 77%. IR ν_max_ (cm^−1^): 3377.36 (N–H stretching), 3174.83, 3076.46 (aromatic C–H stretching), 2978.09, 2924.09, 2873.94 (aliphatic C–H stretching), 1664.57 (amide C=O stretching), 1604.77, 1589.34, 1558.48, 1490.97, 1456.26 (N–H bending, C=N and C=C stretching), 1431.18, 1392.61, 1340.53, 1255.66, 1224.80, 1161.15, 1058.92, 1037.70 (C–H bending, C–N and C–O stretching, aromatic C–H in plane bending), 993.34, 941.26, 891.11, 819.75, 785.03, 740.67, 682.80 (aromatic C–H out of plane bending and C–S stretching) (Supplementary Material Figure S17). ^1^H-NMR (300 MHz, DMSO-*d*_6_): 1.34 (t, *J* = 6.93, 6.96, 13.89 Hz, 3H), 4.06 (q, *J* = 6.93, 13.92 Hz, 2H), 4.33 (s, 2H), 4.36 (s, 2H), 6.96 (d, *J* = 6.07 Hz, 1H), 7.00–7.11 (m, 2H), 7.32–7.38 (m, 2H), 7.48–7.55 (m, 2H), 7.65 (d, *J* = 8.82 Hz, 1H), 11.04 (s, 1H), 12.59 (s, 1H) (Supplementary Material Figure S18). ^13^C-NMR (100 MHz, DMSO-*d*_6_): 15.15 (CH_3_), 21.90 (CH_2_), 36.00 (CH_2_), 64.07 (CH_2_), 105.84 (CH), 106.97 (C), 115.86 (CH), 118.61 (CH), 119.24 (2CH), 121.79 (2CH), 124.65 (CH), 127.03 (C), 133.26 (C), 136.62 (C), 142.98 (C), 155.95 (2C), 163.00 (C), 166.41 (C), 167.80 (C) (Supplementary Material Figure S19). HRMS (ESI) (*m/z*) [M + H]^+^ calcd. for C_22_H_19_N_5_O_3_S_2_: 466.1002, found: 466.1004(Supplementary Material Figure S20).

##### 2-[(5-((1*H*-Indol-3-yl)methyl)-1,3,4-oxadiazol-2-yl)thio]-*N*-(6-nitrobenzothiazol-2-yl)acetamide (**2f**)

Mp: 221.9–222.8 °C. Yield: 88%. IR ν_max_ (cm^−1^): 3442.94 (N–H stretching), 3169.04, 3078.39 (aromatic C–H stretching), 2922.16, 2856.58, 2783.28, 2725.42 (aliphatic C–H stretching), 1685.79 (amide C=O stretching), 1614.42, 1573.91, 1562.34, 1514.12, 1485.19 (N–H bending, NO_2_, C=N and C=C stretching), 1446.61, 1421.54, 1332.81, 1288.45, 1273.02, 1168.86, 1124.50, 1041.56 (C–H bending, NO_2_, C–N and C–O stretching, aromatic C–H in plane bending), 991.41, 904.61, 889.18, 827.46, 729.09, 686.66, 659.66 (aromatic C–H out of plane bending and C–S stretching) (Supplementary Material Figure S21). ^1^H-NMR (300 MHz, DMSO-*d*_6_): 4.32 (s, 2H), 4.38 (s, 2H), 6.97 (t, *J* = 7.46, 14.92 Hz, 1H), 7.04 (t, *J* = 7.08, 14.16 Hz, 1H), 7.32–7.36 (m, 2H), 7.49 (d, *J* = 7.83 Hz, 1H), 7.86 (d, *J* = 8.94 Hz, 1H), 8.25 (d, *J* = 8.96 Hz, 1H), 8.99 (s, 1H), 11.03 (s, 1H), 13.12 (s, 1H) (Supplementary Material Figure S22). ^13^C-NMR (100 MHz, DMSO-*d*_6_): 21.90 (CH_2_), 36.76 (CH_2_), 106.98 (C), 112.04 (CH), 118.60 (CH), 119.23 (CH), 119.29 (CH), 120.79 (CH), 121.77 (CH), 122.11 (CH), 124.64 (CH), 127.03 (C), 132.83 (C), 136.61 (C), 143.14 (C), 154.36 (C), 163.16 (C), 165.28 (C), 167.74 (C), 168.50 (C) (Supplementary Material Figure S23). HRMS (ESI) (*m/z*) [M + H]^+^ calcd. for C_20_H_14_N_6_O_4_S_2_: 467.0591, found: 467.0597 (Supplementary Material Figure S24).

##### 2-[(5-((1*H*-Indol-3-yl)methyl)-1,3,4-oxadiazol-2-yl)thio]-*N*-(thiazol-2-yl)acetamide (**2g**)

Mp: 177.4–178.2 °C. Yield: 82%. IR ν_max_ (cm^−1^): 3429.43, 3406.29 (N–H stretching), 3190.26, 3103.46 (aromatic C–H stretching), 2941.44, 2839.22, 2740.85 (aliphatic C–H stretching), 1687.71 (amide C=O stretching), 1575.84, 1483.26, 1456.26 (N–H bending, C=N and C=C stretching), 1415.75, 1394.53, 1327.03, 1261.45, 1220.94, 1166.93, 1147.65, 1093.64, 1064.71, 1008.77 (C–H bending, C–N and C–O stretching, aromatic C–H in plane bending), 968.27, 879.54, 815.89, 748.38, 709.80, 684.73 (aromatic C–H out of plane bending and C–S stretching) (Supplementary Material Figure S25). ^1^H-NMR (300 MHz, DMSO-*d*_6_): 4.32 (s, 4H), 6.98 (t, *J* = 7.46, 7.40, 14.86 Hz, 1H), 7.09 (t, *J* = 7.46, 7.59, 15.05 Hz, 1H), 7.24–7.32 (m, 2H), 7.37 (d, *J* = 8.07 Hz, 1H), 7.48–7.50 (m, 2H), 11.04 (s, 1H), 12.48 (s, 1H) (Supplementary Material Figure S26). ^13^C-NMR (100 MHz, DMSO-*d*_6_): 21.89 (CH_2_), 35.78 (CH_2_), 106.98 (C), 112.06 (CH), 114.35 (CH), 118.61 (CH), 119.25 (CH), 121.79 (CH), 124.64 (CH), 127.03 (C), 136.63 (C), 138.23 (CH), 158.18 (C), 163.02 (C), 165.67 (C), 167.77 (C) (Supplementary Material Figure S27). HRMS (ESI) (*m/z*) [M + H]^+^ calcd. for C_16_H_13_N_5_O_2_S_2_: 372.0583, found: 372.0575(Supplementary Material Figure S28).

##### 2-[(5-((1*H*-Indol-3-yl)methyl)-1,3,4-oxadiazol-2-yl)thio]-*N*-[5-nitrothiazol-2-yl]acetamide (**2h**)

Mp: 233.9–234.5 °C. Yield: 85%. IR ν_max_ (cm^−1^): 3425.58 (N–H stretching), 3149.76, 3097.68 (aromatic C–H stretching), 2922.16, 2792.93, 2709.99 (aliphatic C–H stretching), 1681.93 (amide C=O stretching), 1597.06, 1571.99, 1514.12, 1485.19, 1477.47 (N–H bending, NO_2_, C=N and C=C stretching), 1421.54, 1373.32, 1342.46, 1305.81, 1276.88, 1161.15, 1120.64, 1095.57, 1031.92 (C–H bending, NO_2_, C–N and C–O stretching, aromatic C–H in plane bending), 974.05, 898.83, 810.10, 744.52, 725.23, 682.80, 630.72 (aromatic C–H out of plane bending and C–S stretching) (Supplementary Material Figure S29). ^1^H-NMR (300 MHz, DMSO-*d*_6_): 4.32 (s, 2H), 4.39 (s, 2H), 6.97 (t, *J* = 7.44, 7.40, 14.84 Hz, 1H), 7.07 (t, *J*= 7.49, 7.58, 15.07 Hz, 1H), 7.30–7.36 (m, 2H), 7.48 (d, *J* = 7.86 Hz, 1H), 8.63 (s, 1H), 11.03 (s, 1H), 13.47 (s, 1H) (Supplementary Material Figure S30). ^13^C-NMR (100 MHz, DMSO-*d*_6_): 21.89 (CH_2_), 35.76 (CH_2_), 106.92 (C), 112.02 (CH), 118.57 (CH), 119.22 (CH), 121.76 (CH), 124.64 (CH), 127.01 (C), 136.62 (C), 142.61 (C), 143.06 (CH), 161.91 (C), 162.80 (C), 167.75 (C), 167.88 (C) (Supplementary Material Figure S31). HRMS (ESI) (*m/z*) [M + H]^+^ calcd. for C_16_H_12_N_6_O_4_S_2_: 417.0434, found: 417.0440 (Supplementary Material Figure S32).

##### 2-[(5-((1*H*-Indol-3-yl)methyl)-1,3,4-oxadiazol-2-yl)thio]-*N*-[4-(ethoxycarbonylmethyl)thiazol-2-yl]acetamide (**2i**)

Mp: 158.0–158.6 °C. Yield: 88%. IR ν_max_ (cm^−1^): 3363.86 (N–H stretching), 3165.19, 3043.67 (aromatic C–H stretching), 2956.87, 2875.86, 2740.85 (aliphatic C–H stretching), 1718.58 (ester C=O stretching), 1674.21 (amide C=O stretching), 1593.20, 1568.13, 1489.05, 1458.18 (N–H bending, C=N and C=C stretching), 1423.47, 1375.25, 1327.03, 1259.52, 1203.58, 1161.15, 1097.50, 1024.20 (C–H bending, C–N and C–O stretching, aromatic C–H in plane bending), 960.55, 952.84, 933.55, 871.82, 796.60, 750.31, 686.66, 642.30 (aromatic C–H out of plane bending and C–S stretching) (Supplementary Material Figure S33). ^1^H-NMR (300 MHz, DMSO-*d*_6_): 1.18 (t, *J* = 7.11, 7.08, 14.19 Hz, 3H), 3.69 (s, 2H), 4.08 (q, *J* = 7.11, 14.22 Hz, 2H), 4.28 (s, 2H), 4.32 (s, 2H), 6.97 (d, *J* = 7.02 Hz, 1H), 7.00–7.02 (m, 1H), 7.09 (t, *J* = 7.04, 8.06, 15.10 Hz, 1H), 7.31–7.38 (m, 2H), 7.49 (d, *J* = 8.83 Hz, 1H), 11.02 (s, 1H), 12.53 (s, 1H) (Supplementary Material Figure S34). ^13^C-NMR (100 MHz, DMSO-*d*_6_): 14.56 (CH_3_), 21.88 (CH_2_), 35.74 (CH_2_), 37.09 (CH_2_), 60.80 (CH_2_), 106.97 (C), 111.30 (C), 112.05 (CH), 118.61 (CH), 119.24 (CH), 121.79 (CH), 124.64 (CH), 127.03 (C), 136.62 (C), 144.28 (CH), 157.75 (C), 162.97 (C), 165.69 (C), 167.76 (C), 170.45 (C) (Supplementary Material Figure S35). HRMS (ESI) (*m/z*) [M + H]^+^ calcd. for C_20_H_19_N_5_O_4_S_2_: 458.0951, found: 458.0955(Supplementary Material Figure S36).

### 3.2. Biochemistry

#### 3.2.1. Cell Culture and Drug Treatment

A549, A375, and HCT116 cell lines were incubated in Dulbecco’s modified Eagle’s medium (DMEM) (Wako Pure Chemical Industries, Osaka, Japan). Human leukemic Jurkat and normal blood (Precision Bioservices, Frederic, MD, USA) cells were cultured in RPMI 1640 (Wako Pure Chemical Industries). All media were supplemented with 10% fetal bovine serum (FBS) (Sigma Aldrich, St. Louis, MO, USA) and 89 μg/mL streptomycin (Meiji Seika Pharma, Tokyo, Japan) at 37 °C in a humid atmosphere and 5% CO_2_. In experiments, all cancer cell lines and PBMCs were cultured in 24-well and 96-well plates (Iwaki brand Asahi Glass Co., Chiba, Japan) at 2 × 10^4^ cells/mL and 1 × 10^6^ cells/mL concentrations, respectively, for 24 h (the optimal cell number for cytotoxicity assay was determined previously) [58,59]. The stock solution of the compounds and erlotinib in concentrations between 0.1–10 mM were prepared in DMSO (Wako Pure Chemical Industries, Osaka, Japan) and further dilution were made with fresh culture medium. The concentration of DMSO in the final culture medium was 1% which had no effect on the cell viability [60,61].

#### 3.2.2. Cytotoxicity Assay

The effects of compounds **2a–i** and erlotinib on cell viability were assessed by using MTT (Dojindo Molecular Technologies, Kumamoto, Japan), as previously described in the literature [62,63]. Cells were exposed to various concentrations (1–100 μM) of the compounds for 48 h at 37 °C and then stained with MTT solution and incubated for additional 4 h. At the end of this period, supernatants were removed, and 100 μL DMSO was added to each well to solubilize the formazan crystals. The absorbance of the solution was determined on an Infinite M1000 plate reader (Tecan, Mannedorf, Switzerland) at a wavelength of 550 nm with background subtraction at 630 nm. All experiments were performed in triplicate, and IC_50_ values were calculated from MTT results and defined as the drug concentrations that reduced absorbance to 50% of control values [64,65].

#### 3.2.3. Detection of Cell Death

After the cells were incubated with the most potent compound in this series at 10 μM concentration for 12 h, apoptotic/necrotic/healthy detection kit (PromoKine, Heidelberg, Germany) was performed according to manufacturer’s instructions with modification [66]. The cells were then briefly washed twice with 1× binding buffer, a staining solution containing 50 μL of 1× binding buffer, 5 μL of FITC-Annexin V solution, 5 μL of ethidium homodimer III solution, and 5 μL of Hoechst 33342 solution and incubated for 15 min at room temperature in the dark. Then, cells were washed with 1× binding buffer and analyzed by a Biorevo Fluorescence BZ-9000 all-in-one fluorescence microscope (Keyence, Osaka, Japan). Numbers of healthy cells (Hoechst 33342), apoptotic cells (Annexin V), late apoptotic or necrotic cells (Annexin V and Ethidium homodimer III), and necrotic cells (Ethidium homodimer III) were counted, as previously described [67].

#### 3.2.4. Kinase Inhibition Assay

The kinase profiling assay protocol was applied according to the manufacturer’s instructions (Promega Corporation, Madison, WI, USA) with modification [58,68]. In this system, the kinases (EGFR, HER2, HER4, IGF1R, InsR, KDR, PDGFRα, and PDGFRβ) and their substrates were diluted with 95 µL 2.5× kinase buffer and 15 µL of 100 µM ATP solutions, respectively. Then, the reaction of the kinase was performed using 2 µL of compound solution at varying concentrations (0.1–100 µM), 4 µL of kinase working stock, and 4 µL of ATP/substrate working stock in the 384-well plate. After 1 h of incubation at room temperature, the activity of kinase was detected using ADP-Glo kinase assay (Promega Corporation, Madison, WI, USA), and kinase inhibitory activity of compounds in dose–response mode was measured by an Infinite M1000 luminescence plate reader (Tecan, Groding, Austria). The IC_50_ values of tested compounds required to decrease the kinase activity by 50% were calculated using ImageJ software.

#### 3.2.5. In Vitro COX Inhibition Assay

COX (ovine/human) inhibitor screening assay kit (Cayman Chemical, Ann Arbor, MI, USA) was used according to the manufacturer’s instructions for measuring COX-1 and COX-2 inhibition rates. Briefly, COX reactions were performed. Background tubes were prepared for COX-1 and COX-2 in boiling water for three minutes. The inactivated enzymes were used to generate the background values. Then, 160 µL reaction buffer, 10 µL of heme, and 10 µL of COX-1 or COX-2 were added to reaction tubes for COX-1 or COX-2 100% initial activity tubes and inhibitor tubes. Next, 10 µL of inhibitors was added to inhibitor tubes, whilst 10 µL of vehicle was added to 100% initial activity tubes and background tubes. Celecoxib and indomethacin were administered at 0.01–10 µM concentrations, whereas compound **2e** was applied at 10–100 µM concentrations. Then, these tubes were incubated for 10 min at 37 °C. Reaction was initiated by adding 10 µL of arachidonic acid to all the reaction tubes, mixed, and incubated for two minutes at 37 °C. Then 30 µL of the saturated stannous chloride solution was added to each tube. The prostaglandins were quantified by ELISA. The plate was read at 405 nm in an ELISA reader (BioTek Instruments Synergy HTX S1 LFA).

### 3.3. In Silico Studies

The co-crystalized X-ray structure of erlotinib binds to the inactive conformation of the EGFR tyrosine kinase domain (PDB code: 4HJO) was used in the present study [56]. Moreover, the crystal structures of COX-1 protein (PDB code: 3KK6), where COX-1 was co-crystallized with celecoxib [69], and COX-2 protein (PDB code: 3LN1), where COX-2 co-crystallized with celecoxib [70], were retrieved from the Protein Data Bank. The QuickPrep wizard in MOE 2019.01 was used to prepare both protein structures for docking. Before docking, non-essential ligands and cofactors as well as waters were deleted from the protein structure. The native ligand, in both COX proteins and EGFR protein, was used as the core of the binding site. Compound **2e** was docked by applying the rigid-receptor protocol, where thirty docking poses were generated for both COX-1 and COX-2 proteins as well as EGFR protein [71]. Other options were kept at their default parameters. The docking method was evaluated by re-docking the co-crystallized ligands with compound **2e**. The generated docking poses were visually inspected using MOE. The binding free energy (∆G) values in kcal/mol of the re-docked erlotinib, celecoxib, and compound **2e** were calculated using the top-scored docking poses.

MD simulations were executed on the **2e**-EGFR docked structure utilizing AMBER16 software [72]. The technical details of the applied MD simulation are reported in details elsewhere [73,74]. Concisely, AMBER force field 14SB [75] and general AMBER force field2 (GAFF2) [76] were utilized to describe EGFR and compound **2e**, respectively. The atomic partial charges were assigned using the restrained electrostatic potential (RESP) approach with the help of Gaussian09 software [77,78]. The **2e**-EGFR complex was solvated in a cubic water box with 15 A. The solvated 2e-EGFR complex was minimized for 5000 steps and thereafter gently heated from 0 K to 300 K over 50 ps. In addition, **2e**-EGFR complex was simulated for 1 ns of equilibration and 20 ns of production utilizing periodic boundary conditions and NPT ensemble.

## 4. Conclusions

In the current work, a new series of indole, 1,3,4-oxadiazole, and thiazole/benzothiazole skeletons incorporated compounds **2a**–**i** were obtained with good yields through efficient and facile synthetic methods. All compounds were tested in vitro for the evaluation of their cytotoxic effects on HCT116 (CRC), A549 (NSCLC) and A375 (melanoma) cells. The 6-ethoxybenzothiazole substituted compound **2e** displayed the most significant cytotoxicity against these three cell lines compared to erlotinib. Furthermore, compounds **2a** and **2d** exhibited anticancer activity against these cell lines. Therefore, the tumor selectivity of these three compounds between Jurkat cells and PBMCs (healthy) was also enlightened. Compound **2e** exerted the highest selectivity to healthy cells. This compound was chosen for further comprehensive experiments to offer a new insight into its molecular mechanisms of action including the determination of apoptotic activity in the HCT116 cell line and inhibitory effects on cancer related enzymes such as RTKs, in particular EGFR and COX-2. HCT116 cells treated with compound **2e** underwent apoptosis, and the apoptotic activity of compound **2e** was more significant than that of erlotinib. When compared to erlotinib, compound **2e** significantly inhibited EGFR, the most crucial target in this large panel of kinases. Moreover, the binding mode of compound **2e** to the EGFR active site was confirmed with in silico docking simulation and molecular docking studies demonstrating that the 6-ethoxybenzothiazole moiety is mainly responsible for its improved EGFR inhibitory activity, which strongly supports the biological data. Compound **2e** also caused selective COX-2 inhibition compared to celecoxib and indomethacin, whereas its COX-2 inhibition was found to be weak. Molecular docking of compound **2e** in the active sites of COX-1 and COX-2 also confirmed the in vitro assays indicating selective and weak COX-2 inhibition. It can be concluded that compound **2e** exhibits anticancer effects preventing EGFR-dependent activation selectively. This work represents that compound **2e** attracts a great deal of attention with high anticancer potency as a promising EGFR inhibitor for further anticancer studies.

## Figures and Tables

**Table 1:** The cytotoxic effects of compounds **2a–i** on HCT-116, A549, and A375 cell lines.

Compound	IC_50_ (μM)		
	HCT-116 Cell Line	A549 Cell Line	A375 Cell Line
**2a**	26.25 ± 1.75	40.39 ± 7.74	65.12 ± 10.11
**2b**	>100	60.67 ± 9.48	37.55 ± 5.62
**2c**	>100	24.88 ± 1.96	48.53 ± 5.78
**2d**	18.23 ± 1.19	36.86 ± 5.43	30.19 ± 3.12
**2e**	6.43 ± 0.72	9.62 ± 1.14	8.07 ± 1.36
**2f**	>100	>100	>100
**2g**	>100	>100	>100
**2h**	>100	>100	>100
**2i**	>100	71.89 ± 10.27	95.86 ± 12.41
**Erlotinib**	17.86 ± 3.22	19.41 ± 2.38	23.81 ± 4.17

**Table 2:** The cytotoxicity of compounds **2a**, **2d**, and **2e** against Jurkat cells and PBMCs.

Compound	IC_50_ (μM)		SI *
	Jurkat Cells	PBMCs	
**2a**	17.68 ± 3.59	>300	>16.97
**2d**	21.06 ± 1.85	>300	>14.25
**2e**	6.45 ± 1.02	>300	>46.51
**Erlotinib**	9.47 ± 2.15	45.71 ± 8.88	4.83

* SI = IC_50_ for PBMCs/IC_50_ for Jurkat cells.

**Table 3:** The tyrosine kinase inhibition of compound **2e** and erlotinib.

Kinase	IC_50_ (μM)	
	Compound 2e	Erlotinib
**EGFR**	2.80 ± 0.52	0.04 ± 0.01
**HER2**	>30	>30
**HER4**	8.22 ± 1.64	2.18 ± 0.73
**IGF1R**	>30	>30
**InsR**	29.10 ± 4.66	>30
**KDR**	>30	4.48 ± 1.02
**PDGFRα**	>30	6.85 ± 1.82
**PDGFRβ**	6.16 ± 1.58	>30

**Table 4:** The inhibitory effects of compound **2e**, celecoxib, and indomethacin on COX-1 and COX-2.

Compound	IC_50_ (µM)		SI *
	COX-1	COX-2	
**2e**	73.5 ± 2.12	37.5 ± 3.54	1.96
**Celecoxib**	8.875 ± 0.375	2.75 ± 0.05	3.23
**Indomethacin**	0.12 ± 0.01	0.575 ± 0.075	0.21

* SI = IC_50_ for COX-1/IC_50_ for COX-2.

## Supplementary Materials

Figure S1: IR spectrum of compound **2a**, Figure S2: ^1^H-NMR spectrum of compound **2a**, Figure S3: ^13^C-NMR spectrum of compound **2a**, Figure S4: HRMS spectrum of compound **2a**, Figure S5: IR spectrum of compound **2b**, Figure S6: ^1^H-NMR spectrum of compound **2b**, Figure S7: ^13^C-NMR spectrum of compound **2b**, Figure S8: HRMS spectrum of compound **2b**, Figure S9: IR spectrum of compound **2c**, Figure S10: ^1^H-NMR spectrum of compound **2c**, Figure S11: ^13^C-NMR spectrum of compound **2c**, Figure S12: HRMS spectrum of compound **2c**, Figure S13: IR spectrum of compound **2d**, Figure S14: ^1^H-NMR spectrum of compound **2d**, Figure S15: ^13^C-NMR spectrum of compound **2d**, Figure S16: HRMS spectrum of compound **2d**, Figure S17: IR spectrum of compound **2e**, Figure S18: ^1^H-NMR spectrum of compound **2e**, Figure S19: ^13^C-NMR spectrum of compound **2e**, Figure S20: HRMS spectrum of compound **2e**, Figure S21: IR spectrum of compound **2f**, Figure S22: ^1^H-NMR spectrum of compound **2f**, Figure S23: ^13^C-NMR spectrum of compound **2f**, Figure S24: HRMS spectrum of compound **2f**, Figure S25: IR spectrum of compound **2g**, Figure S26: ^1^H-NMR spectrum of compound **2g**, Figure S27: ^13^C-NMR spectrum of compound **2g**, Figure S28: HRMS spectrum of compound **2g**, Figure S29: IR spectrum of compound **2h**, Figure S30: ^1^H-NMR spectrum of compound **2h**, Figure S31: ^13^C-NMR spectrum of compound **2h**, Figure S32: HRMS spectrum of compound **2h**, Figure S33: IR spectrum of compound **2i**, Figure S34: ^1^H-NMR spectrum of compound **2i**, Figure S35: ^13^C-NMR spectrum of compound **2i**, Figure S36: HRMS spectrum of compound **2i.**
