# Supplementary material for: In Vitro and In Silico Evaluation of Anticancer Activity of New Indole-Based 1,3,4-Oxadiazoles as EGFR and COX-2 Inhibitors
Source: Molecules. 2020 Nov 7;25(21):5190. doi: 10.3390/molecules25215190 (PMC7664637; doi:10.3390/molecules25215190)
Supplement: Supplementary file 1 [file molecules-25-05190-s001.pdf]

Supplementary material

In Vitro and *In Silico* Evaluation of Anticancer Activity of New Indole-based 1,3,4-Oxadiazoles as EGFR and COX-2 Inhibitors

Belgin Sever [1,*], Mehlika Dilek Altıntop [1], Ahmet Özdemir [1], Gülşen Akalın Çiftçi [2], Doha E. Ellakwa [3], Hiroshi Tateishi [4], Mohamed O. Radwan [4,5,6], Mahmoud A. A. Ibrahim[7], Masami Otsuka [4,5], Mikako Fujita [4,*], Halil I. Ciftci [4,5,*], Taha F.S. Ali [4,8]

1 Department of Pharmaceutical Chemistry, Faculty of Pharmacy, Anadolu University, Eskişehir 26470, Turkey; mdaltintop@anadolu.edu.tr (M.D.A.); ahmeto@anadolu.edu.tr (A.Ö.)
2 Department of Biochemistry, Faculty of Pharmacy, Anadolu University, Eskişehir 26470, Turkey; gakalin@anadolu.edu.tr (G.A.Ç.)
3 Department of Biochemistry and Molecular Biology, Faculty of Pharmacy, Al-Azhar University, Cairo 11765, Egypt; profdoha@gmail.com (D.E.E.)
4 Medicinal and Biological Chemistry Science Farm Joint Research Laboratory, School of Pharmacy, Kumamoto University, Kumamoto 862-0973, Japan; htateishi@kumamoto-u.ac.jp (H.T.); mohamedradwan@kumamoto-u.ac.jp (M.O.R.); motsuka@gpo.kumamoto-u.ac.jp (M.O.); tahafaroukali@gmail.com (T.F.S.A.)
5 Department of Drug Discovery, Science Farm Ltd., Kumamoto 862-0976, Japan
6 Chemistry of Natural Compounds Department, Pharmaceutical and Drug Industries Research Division, National Research Centre, Dokki, Cairo 12622, Egypt
7 Computational Chemistry Laboratory, Chemistry Department, Faculty of Science, Minia University, Minia 61519, Egypt; m.ibrahim@compchem.net (M.A.A.I.)
8 Medicinal Chemistry Department, Faculty of Pharmacy, Minia University, Minia 61519, Egypt
* Correspondence: belginsever@anadolu.edu.tr (B.S.); mfujita@kumamoto-u.ac.jp (M.F.); hiciftci@kumamoto-u.ac.jp (H.I.C.); Tel.: +90-222-335-0580 (ext. 3807)

Table of Contents

Figure S1. IR spectrum of compound **2a**.

Figure S2. ^{1}H NMR spectrum of compound **2a**.

Figure S3. ^{13}C NMR spectrum of compound **2a**.

Figure S4. HRMS spectrum of compound **2a**.

Figure S5. IR spectrum of compound **2b**.

Figure S6. ^{1}H NMR spectrum of compound **2b**.

Figure S7. ^{13}C NMR spectrum of compound **2b**.

Figure S8. HRMS spectrum of compound **2b**.

Figure S9. IR spectrum of compound **2c**.

Figure S10. ^{1}H NMR spectrum of compound **2c**.

Figure S11. ^{13}C NMR spectrum of compound **2c**.

Figure S12. HRMS spectrum of compound **2c**.

Figure S13. IR spectrum of compound **2d**.

Figure S14. ^{1}H NMR spectrum of compound **2d**.

Figure S15. ^{13}C NMR spectrum of compound **2d**.

Figure S16. HRMS spectrum of compound **2d**.

Figure S17. IR spectrum of compound **2e**.

Figure S18. ^{1}H NMR spectrum of compound **2e**.

Figure S19. ^{13}C NMR spectrum of compound **2e**.

Figure S20. HRMS spectrum of compound **2e**.

Figure S21. IR spectrum of compound **2f**.

Figure S22. ^{1}H NMR spectrum of compound **2f**.

Figure S23. ^{13}C NMR spectrum of compound **2f**.

Figure S24. HRMS spectrum of compound **2f**.

Figure S25. IR spectrum of compound **2g**.

Figure S26. ^{1}H NMR spectrum of compound **2g**.

Figure S27. ^{13}C NMR spectrum of compound **2g**.

Figure S28. HRMS spectrum of compound **2g**.

Figure S29. IR spectrum of compound **2h.**

Figure S30. ^{1}H NMR spectrum of compound **2h**.

Figure S31. ^{13}C NMR spectrum of compound **2h**.

Figure S32. HRMS spectrum of compound **2h.**

Figure S33. IR spectrum of compound **2i**.

Figure S34. ^{1}H NMR spectrum of compound **2i**.

Figure S35. ^{13}C NMR spectrum of compound **2i**.

Figure S36. HRMS spectrum of compound **2i**.

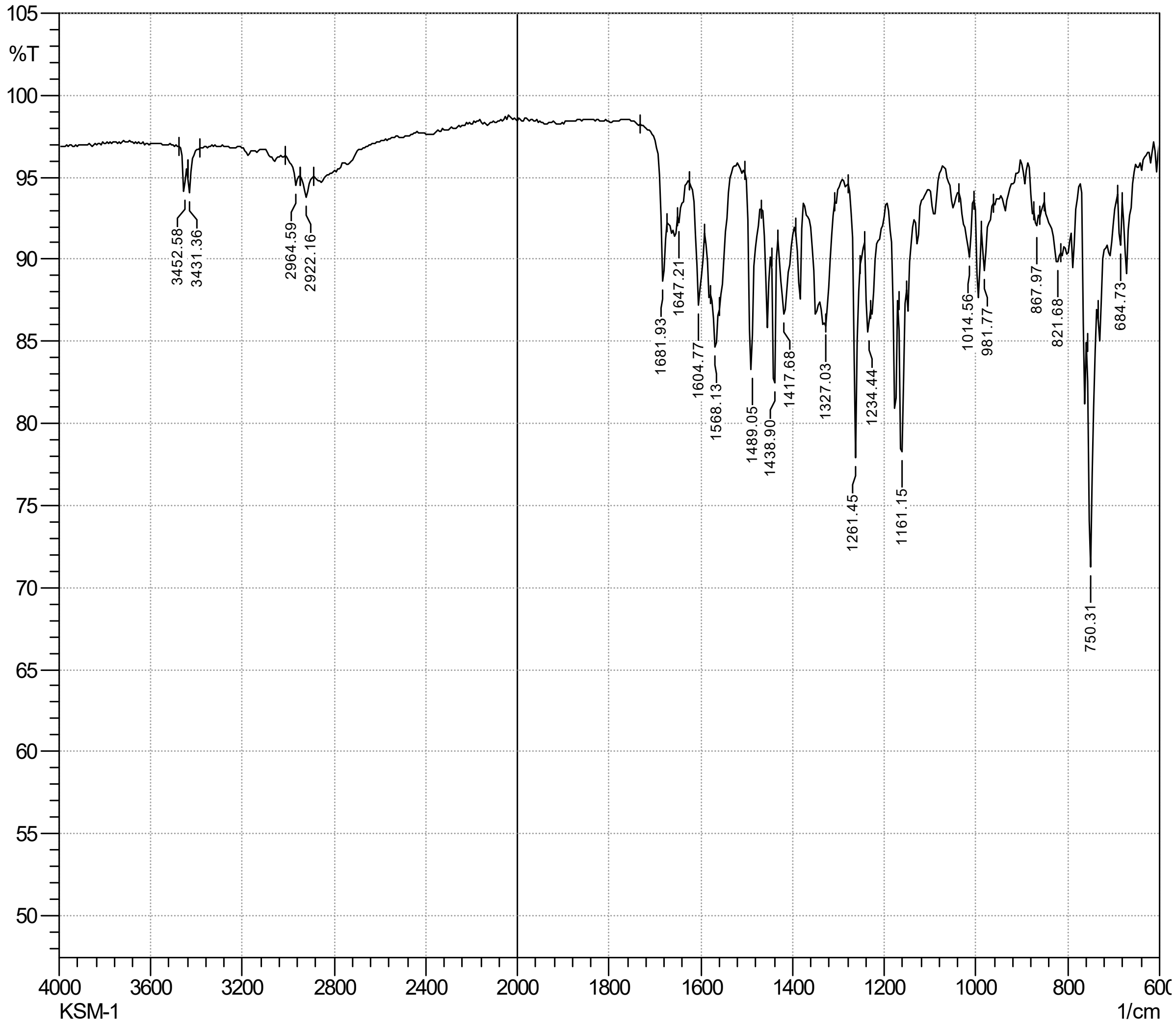

Figure S1. IR spectrum of compound **2a**.

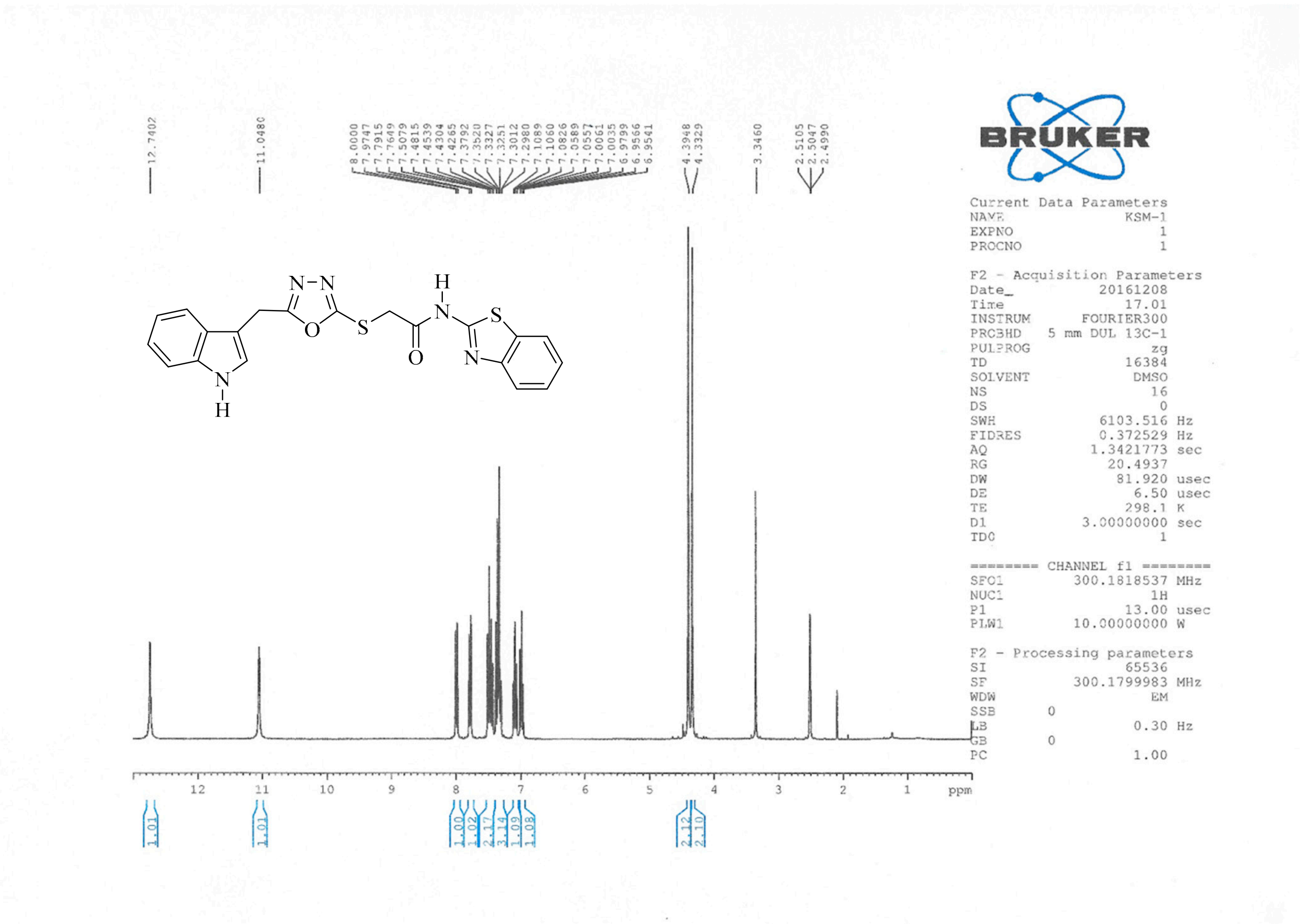

Figure S2. ^{1}H NMR spectrum of compound **2a**.

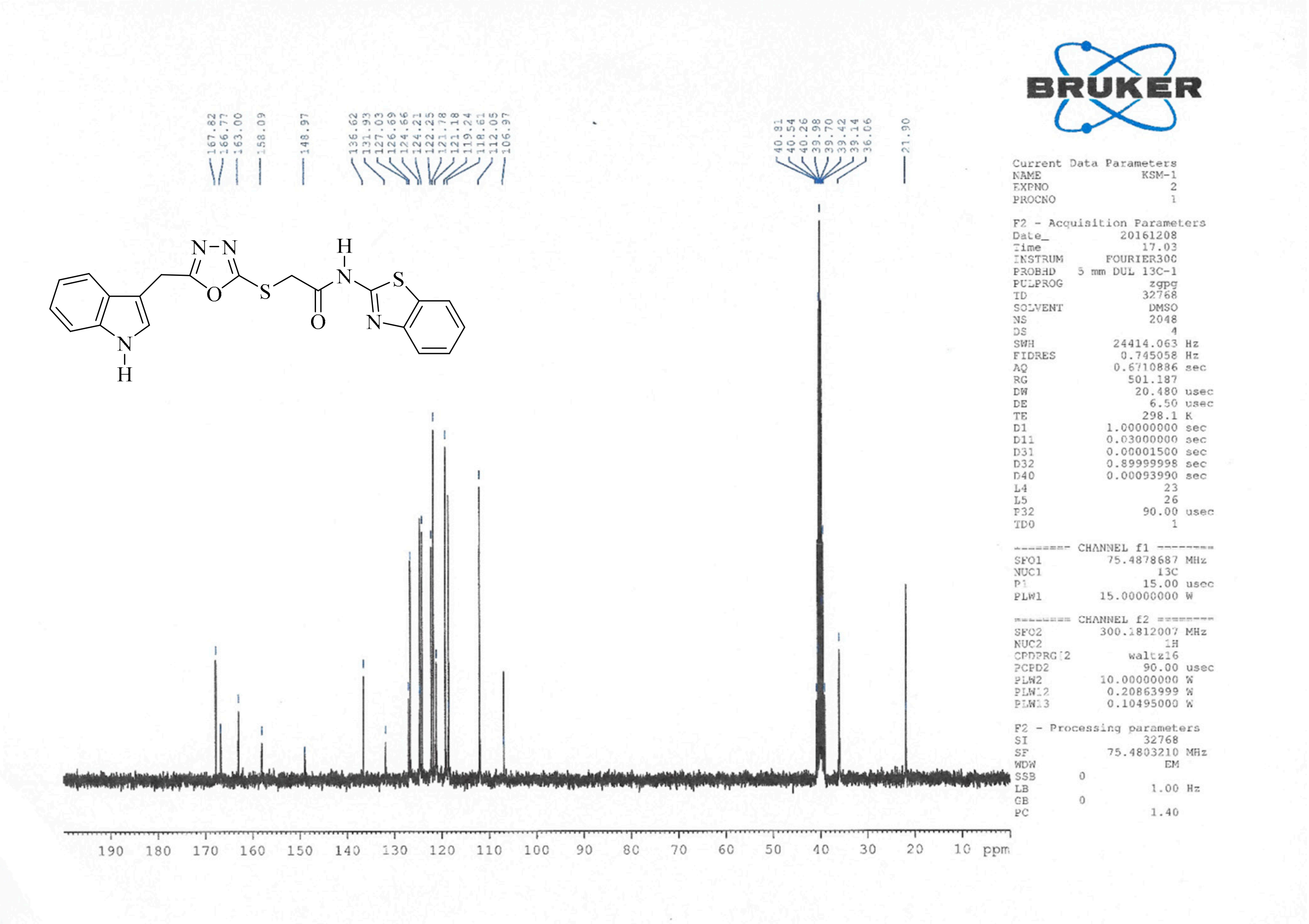

Figure S3. ^{13}C NMR spectrum of compound **2a**.

Formula Predictor Report - KSM-1_1.lcd

Data File: C:\LabSolutions\Data\Analiz\mdaltintop\KSM-1_1.lcd

Elmt	Val.	Min	Max	Elmt	Val.	Min	Max	Elmt	Val.	Min	Max	Elmt	Val.	Min	Max	Use Adduct
H	1	10	20	O	2	2	4	Cl	1	0	0	I	3	0	0	H
C	4	18	30	F	1	0	0	Br	1	0	0					
N	3	5	6	S	2	2	2	Ru	2	0	0					

Error Margin (ppm): 5
HC Ratio: unlimited
Max Isotopes: 3
MSn Iso RI (%): 10.00

DBE Range: 0.0 - 30.0
Apply N Rule: yes
Isotope RI (%): 1.00
MSn Logic Mode: AND

Electron Ions: both
Use MSn Info: no
Isotope Res: 10000
Max Results: 500

Event#: 1 MS(E+) Ret. Time : 6.280 -> 6.427 Scan# : 943 -> 965

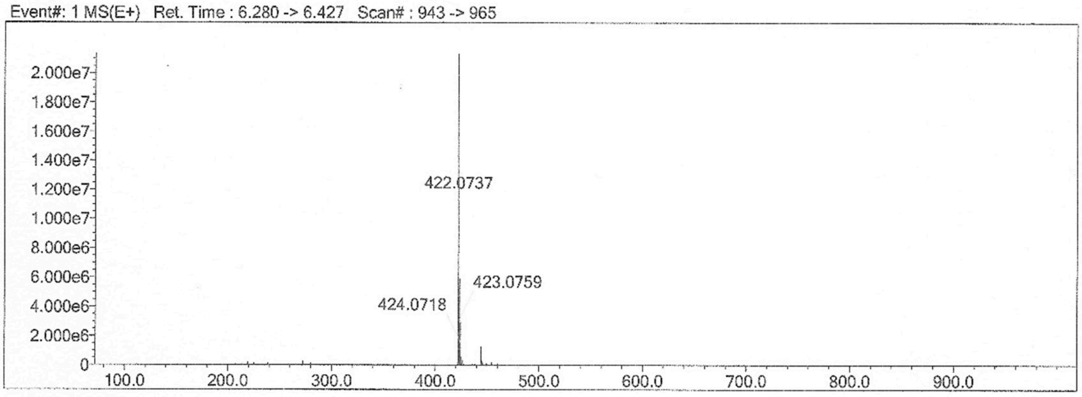

Measured region for 422.0737 m/z

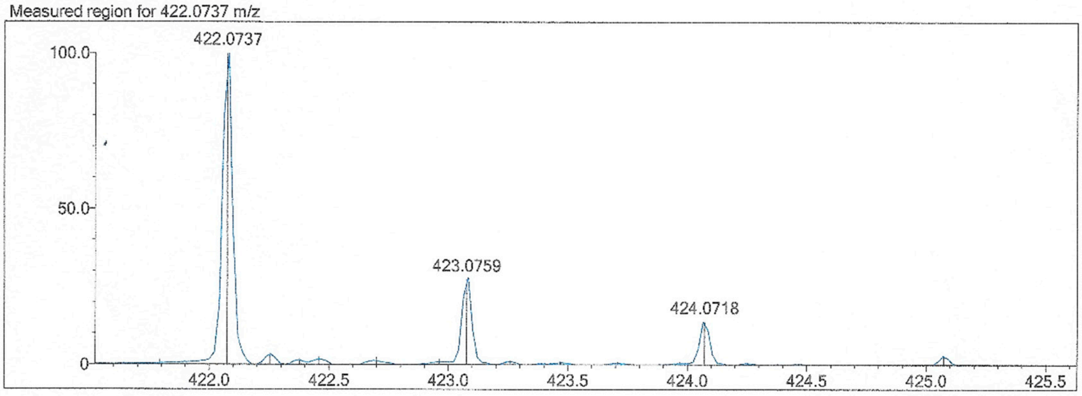

C20 H15 N5 O2 S2 [M+H]+ : Predicted region for 422.0740 m/z

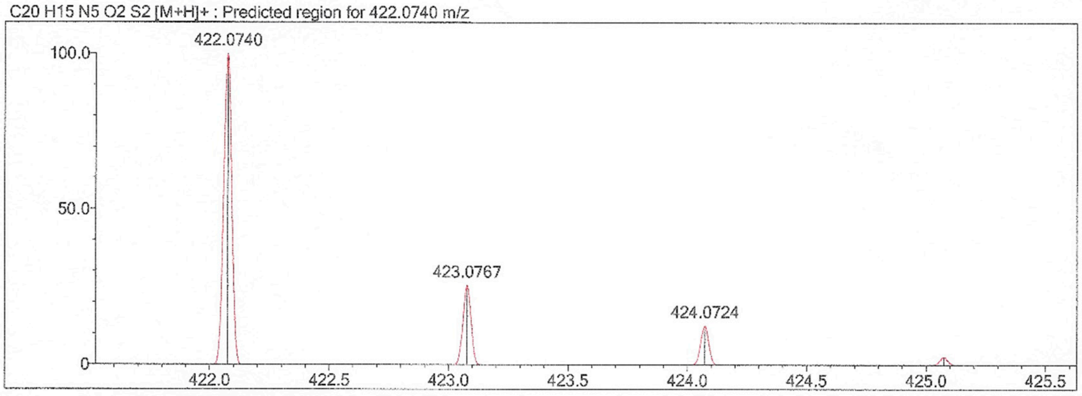

Rank	Score	Formula (M)	Ion	Meas. m/z	Pred. m/z	Df. (mDa)	Df. (ppm)	Iso	DBE
1	93.51	C20 H15 N5 O2 S2	[M+H]+	422.0737	422.0740	-0.3	-0.71	93.51	16.0

Figure S4. HRMS spectrum of compound **2a**.

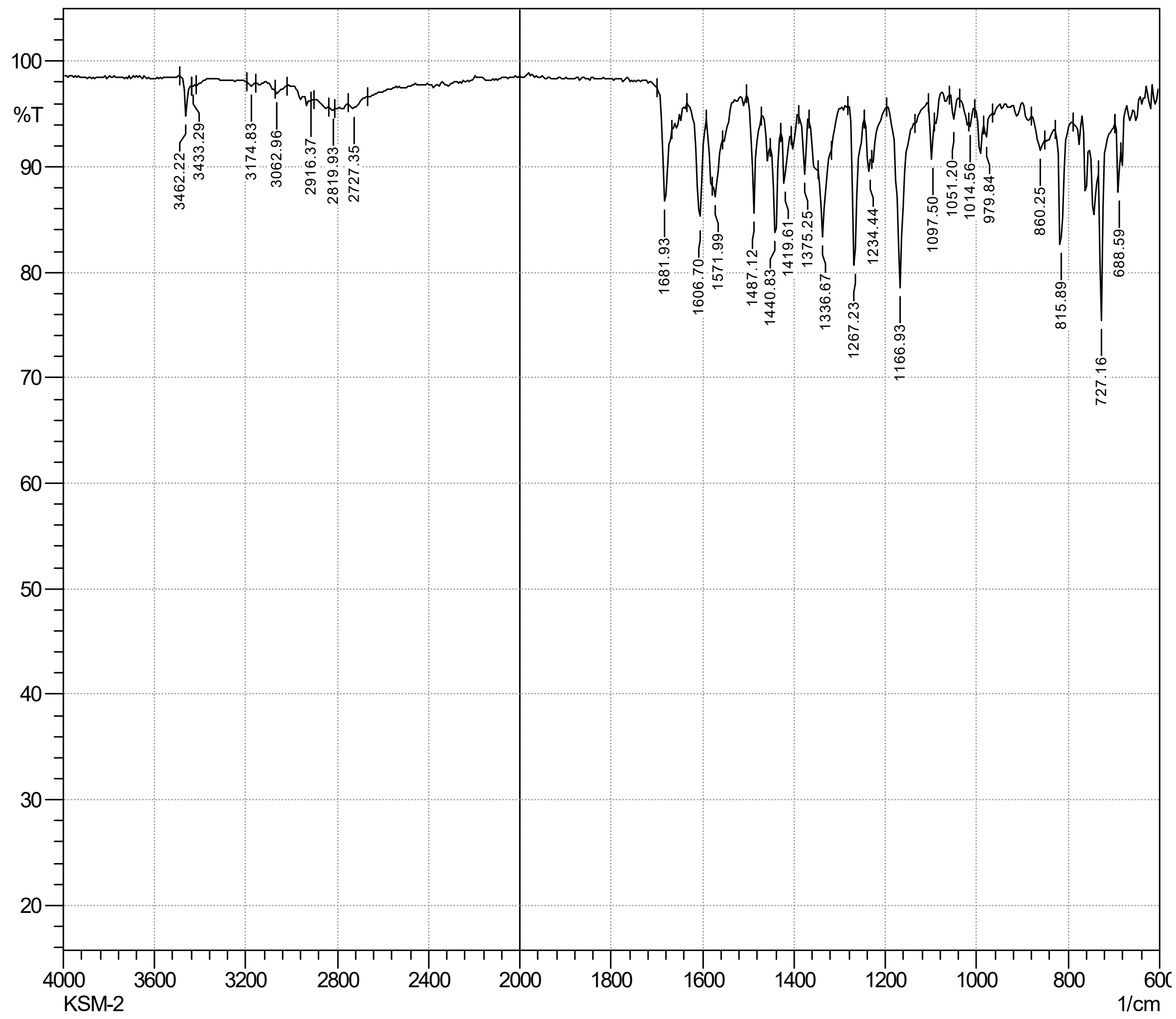

Figure S5. IR spectrum of compound **2b**.

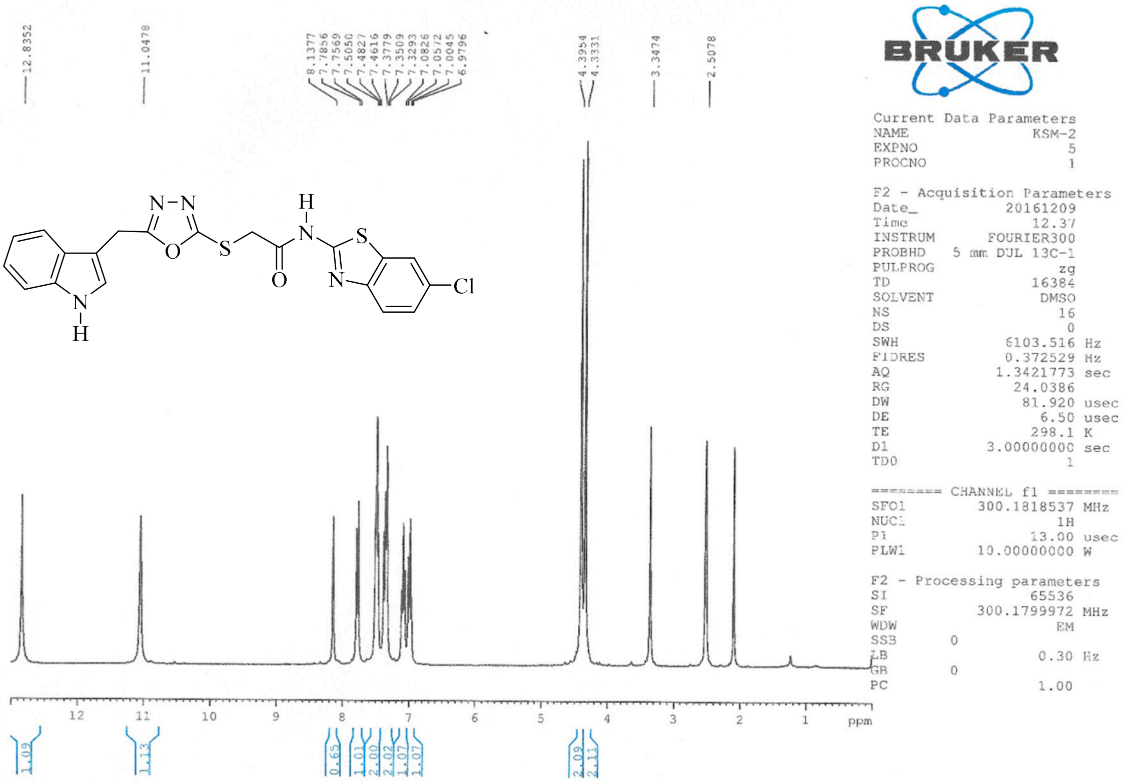

Figure S6. ^{1}H NMR spectrum of compound **2b**.

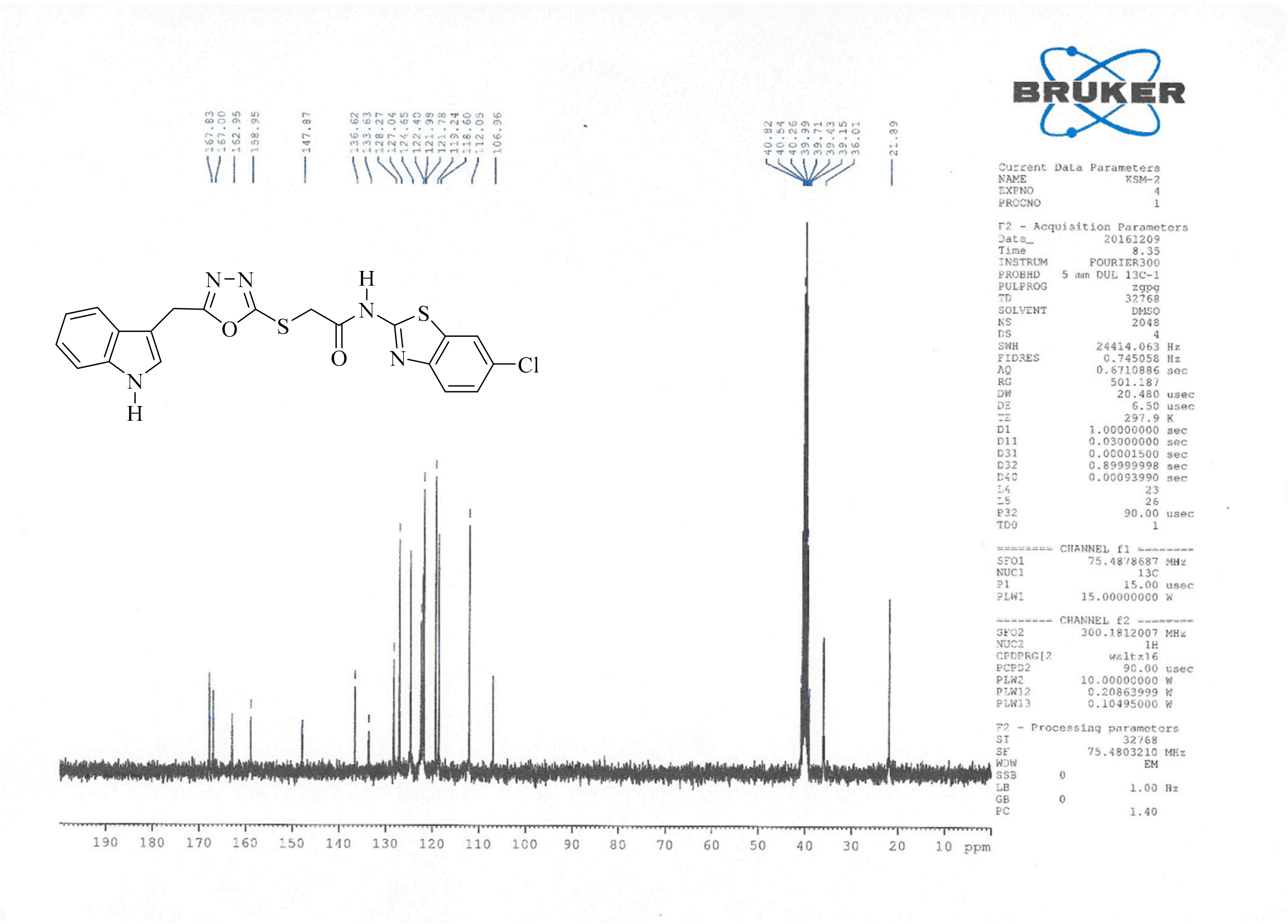

Figure S7. ^{13}C NMR spectrum of compound **2b**.

Formula Predictor Report - KSM-2_2.lcd

Data File: C:\LabSolutions\Data\Analiz\mdaltintop\KSM-2_2.lcd

Elmt	Val.	Min	Max	Elmt	Val.	Min	Max	Elmt	Val.	Min	Max	Elmt	Val.	Min	Max	Use Adduct
H	1	10	20	O	2	2	4	Cl	1	0	1	I	3	0	0	H
C	4	18	30	F	1	0	0	Br	1	0	0					
N	3	5	6	S	2	2	2	Ru	2	0	0					

Error Margin (ppm): 5
HC Ratio: unlimited
Max Isotopes: 3
MSn Iso RI (%): 10.00

DBE Range: 0.0 - 30.0
Apply N Rule: yes
Isotope RI (%): 1.00
MSn Logic Mode: AND

Electron Ions: both
Use MSn Info: no
Isotope Res: 10000
Max Results: 500

Event#: 1 MS(E+) Ret. Time : 6.787 -> 6.960 Scan# : 1019 -> 1045

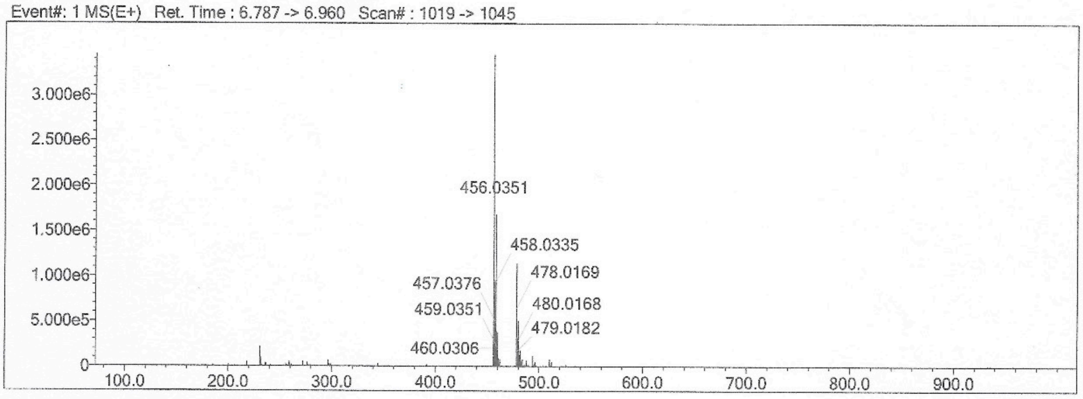

Measured region for 456.0351 m/z

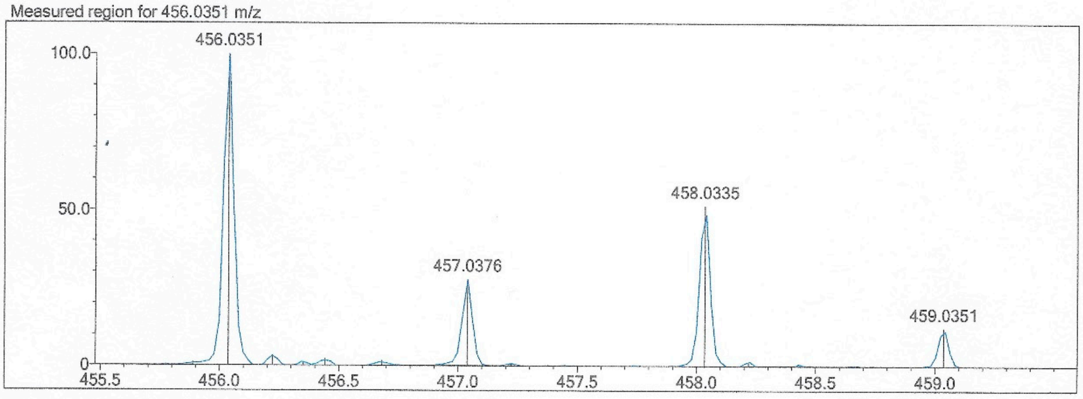

C20 H14 N5 O2 S2 Cl [M+H]+ : Predicted region for 456.0350 m/z

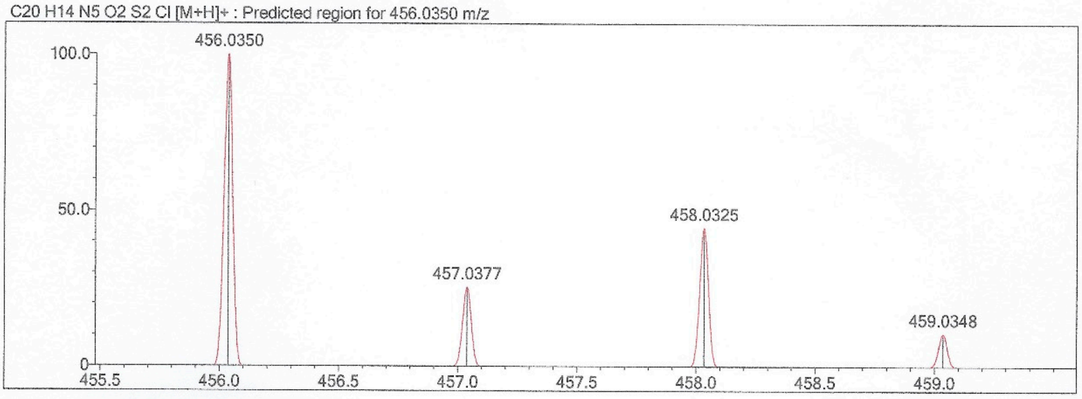

Rank	Score	Formula (M)	Ion	Meas. m/z	Pred. m/z	Df. (mDa)	Df. (ppm)	Iso	DBE
1	96.94	C20 H14 N5 O2 S2 Cl	[M+H]+	456.0351	456.0350	0.1	0.22	96.94	16.0

Figure S8. HRMS spectrum of compound **2b**.

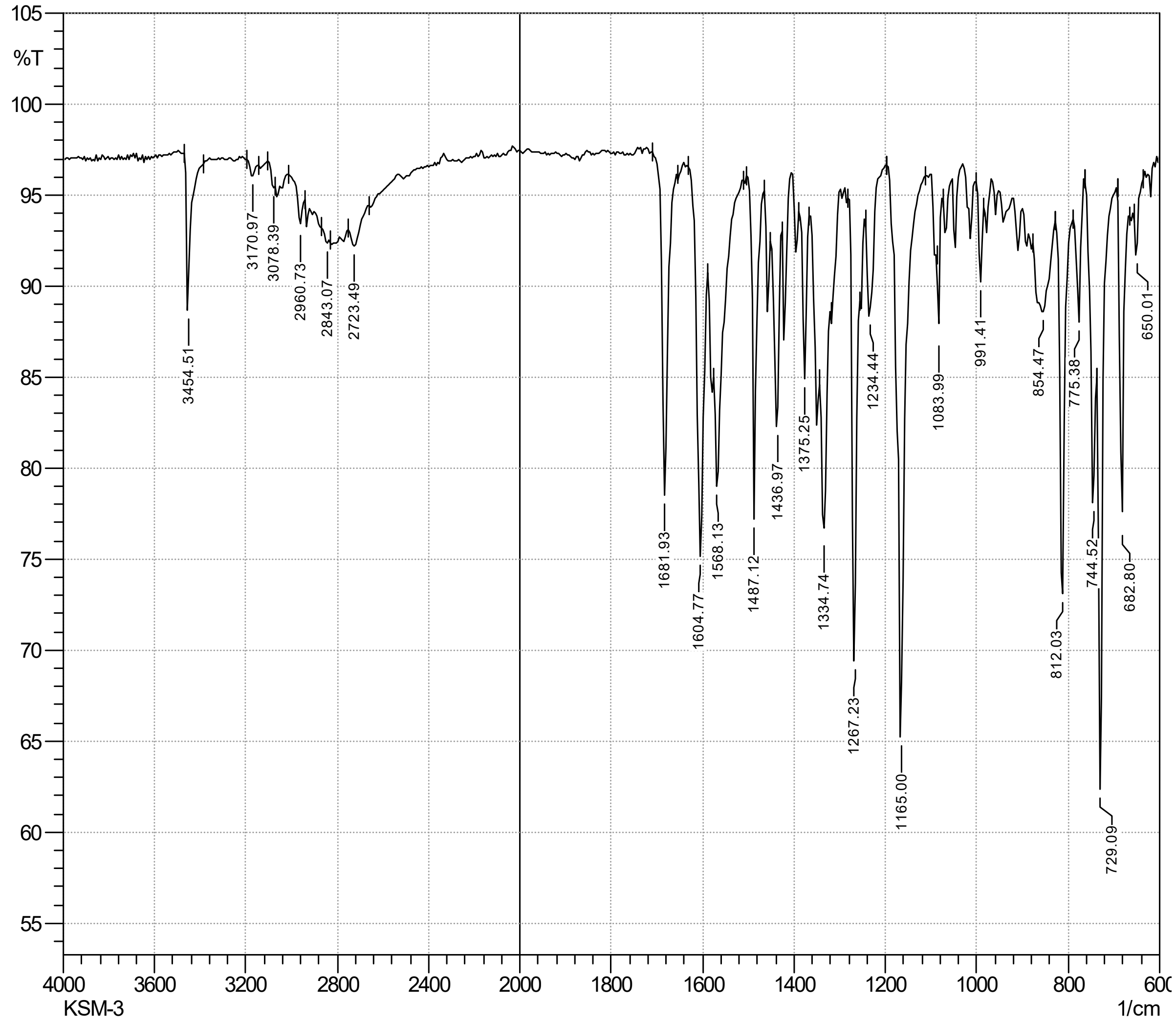

Figure S9. IR spectrum of compound **2c.**

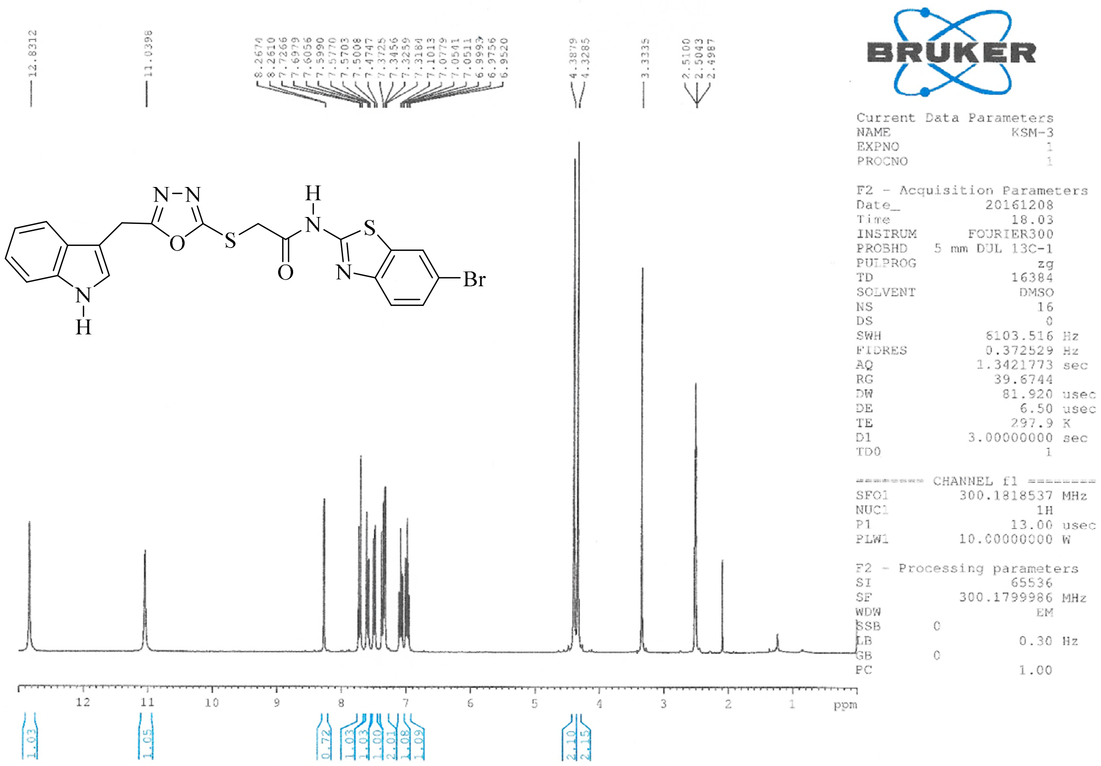

Figure S10. ^{1}H NMR spectrum of compound **2c**.

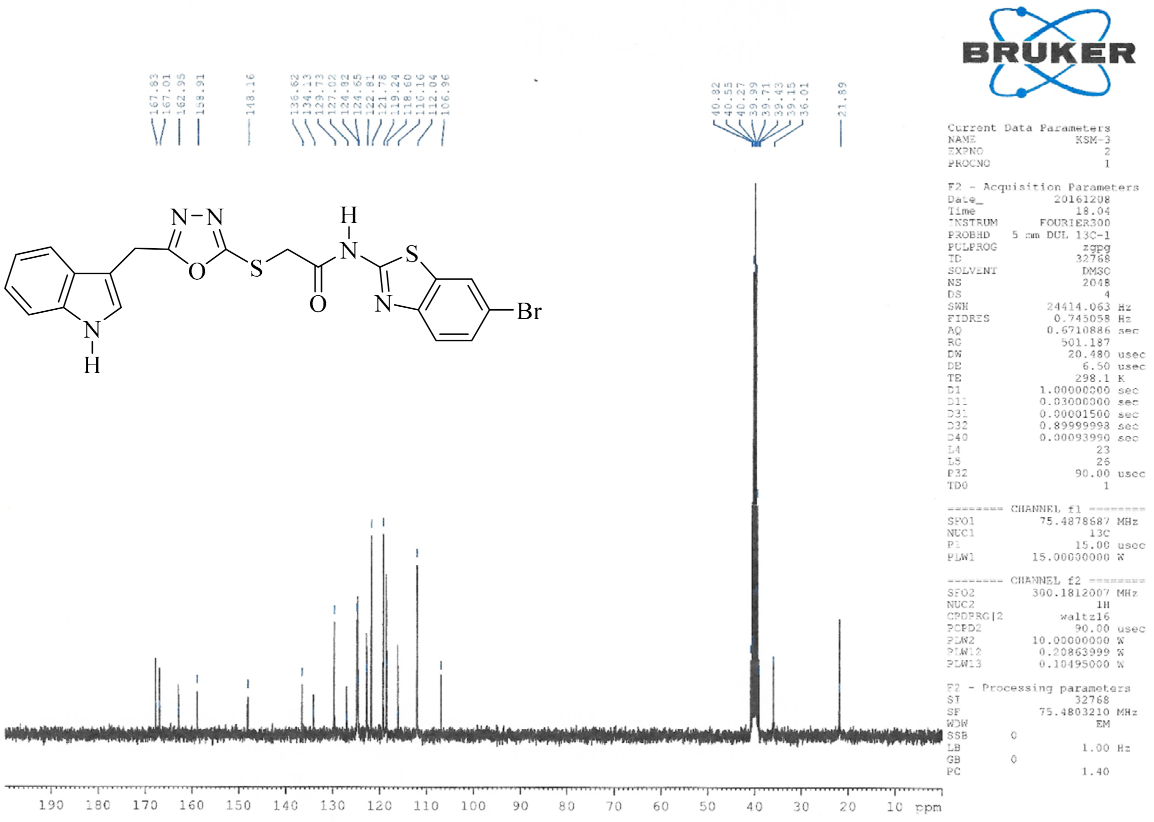

Figure S11. ^{13}C NMR spectrum of compound **2c**.

Formula Predictor Report - KSM-3_3.lcd

Data File: C:\LabSolutions\Data\Analiz\mdaltintop\KSM-3_3.lcd

Elmt	Val.	Min	Max	Elmt	Val.	Min	Max	Elmt	Val.	Min	Max	Elmt	Val.	Min	Max	Use Adduct
H	1	10	20	O	2	2	4	Cl	1	0	1	I	3	0	0	H
C	4	18	30	F	1	0	0	Br	1	0	1					
N	3	5	6	S	2	2	2	Ru	2	0	0					

Error Margin (ppm):	5	DBE Range:	0.0 - 30.0	Electron Ions:	both
HC Ratio:	unlimited	Apply N Rule:	yes	Use MSn Info:	no
Max Isotopes:	3	Isotope RI (%):	1.00	Isotope Res:	10000
MSn Iso RI (%):	10.00	MSn Logic Mode:	AND	Max Results:	500

Event#: 1 MS(E+) Ret. Time : 6.907 -> 6.987 Scan# : 1037 -> 1049

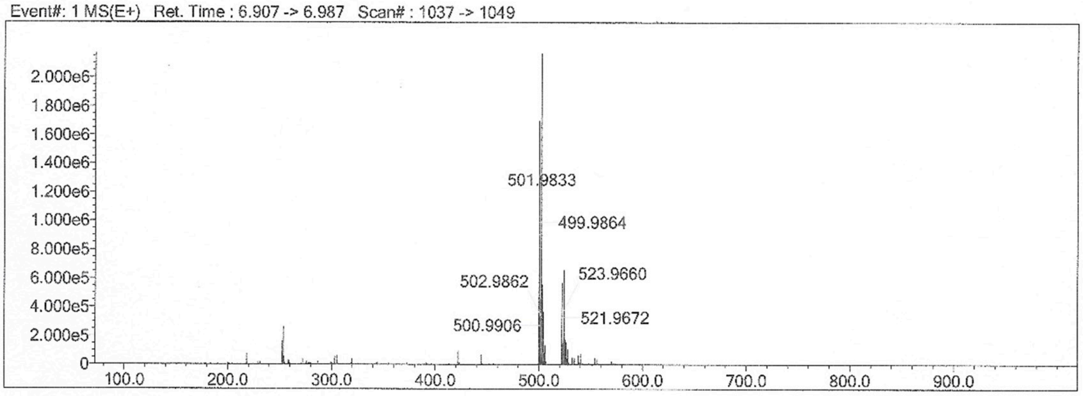

Measured region for 499.9864 m/z

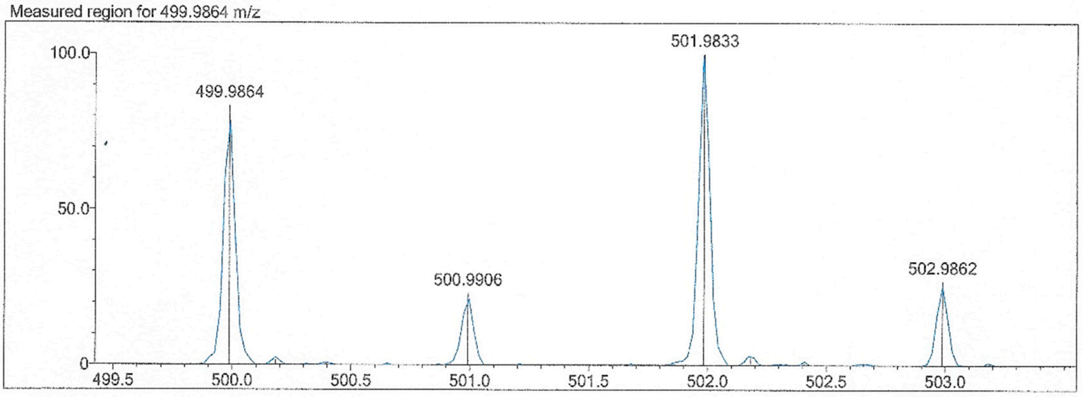

C20 H14 N5 O2 S2 Br [M+H]+ : Predicted region for 499.9845 m/z

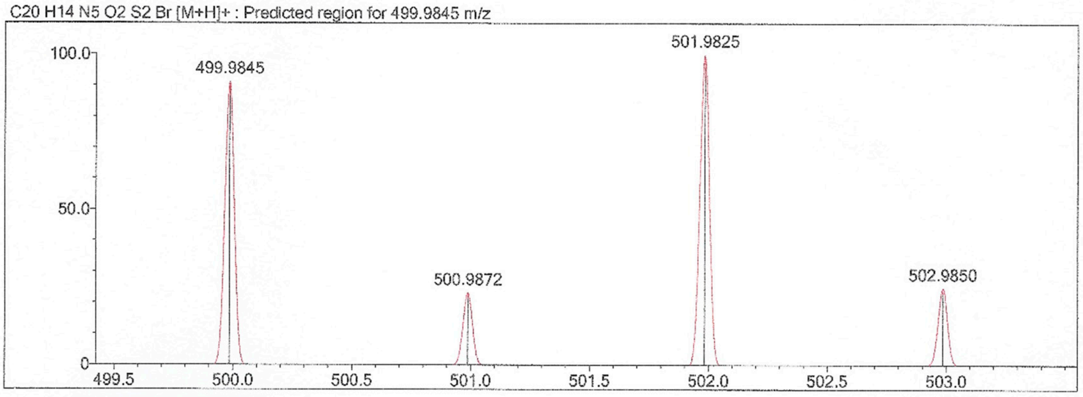

Rank	Score	Formula (M)	Ion	Meas. m/z	Pred. m/z	Df. (mDa)	Df. (ppm)	Iso	DBE
1	63.43	C20 H14 N5 O2 S2 Br	[M+H]+	499.9864	499.9845	1.9	3.80	68.20	16.0

Figure S12. HRMS spectrum of compound **2c**.

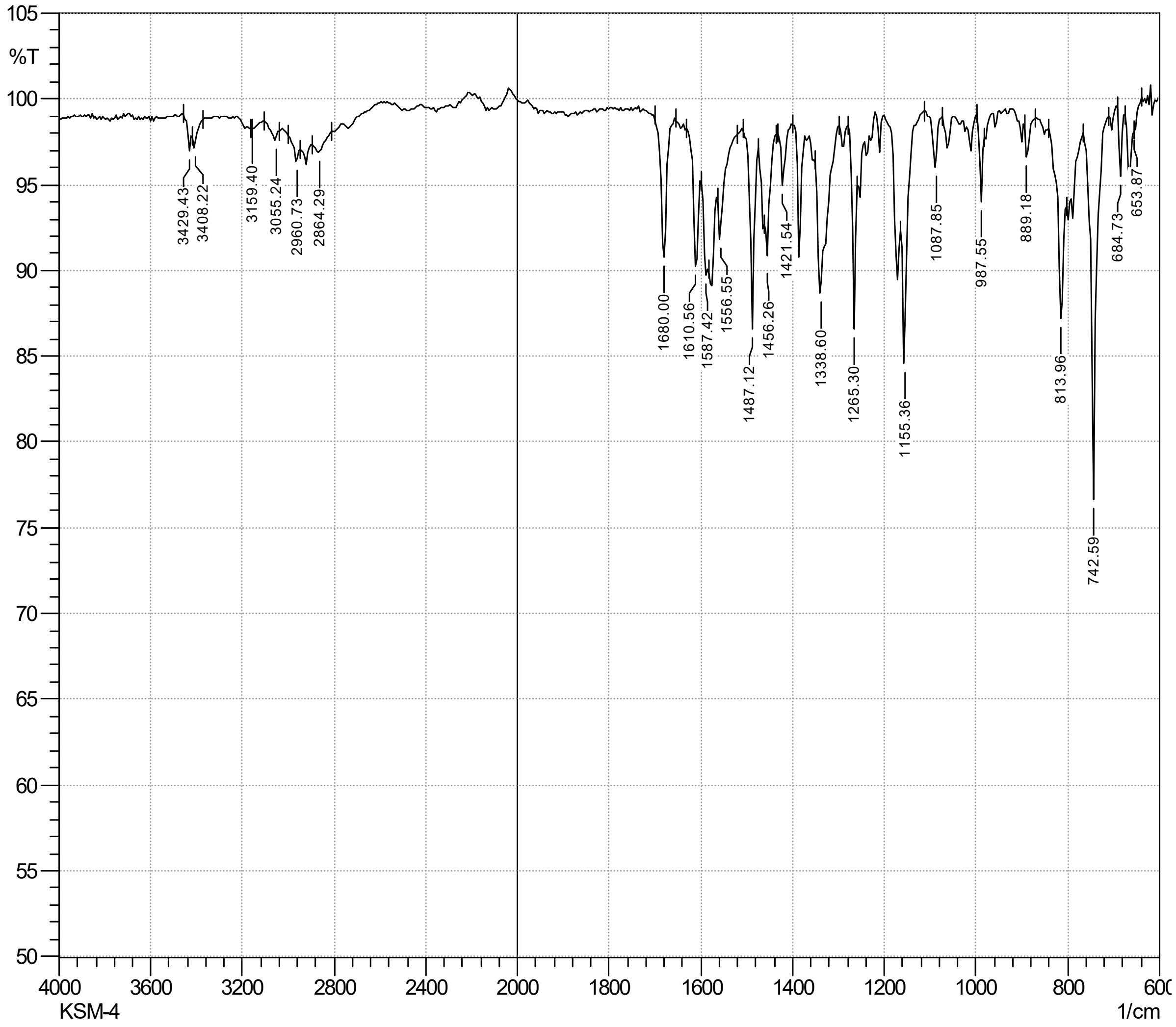

Figure S13. IR spectrum of compound **2d.**

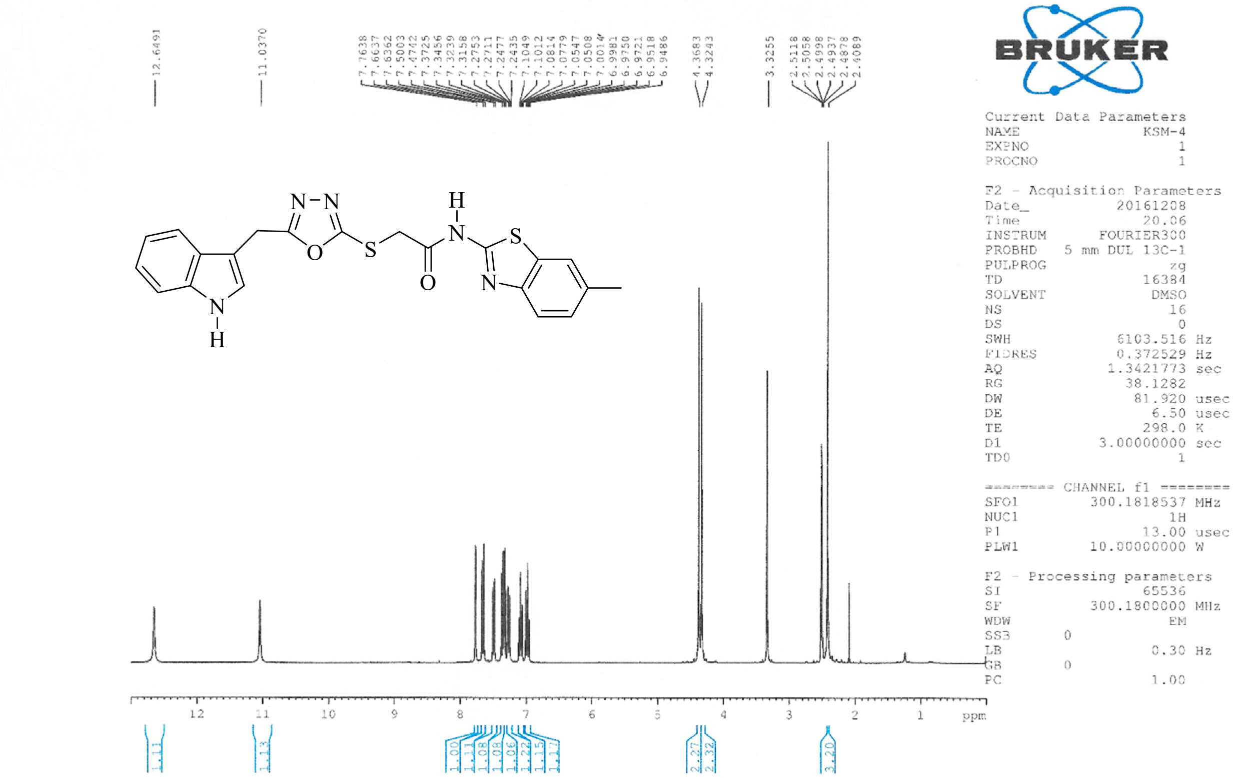

Figure S14. ^{1}H NMR spectrum of compound **2d**.

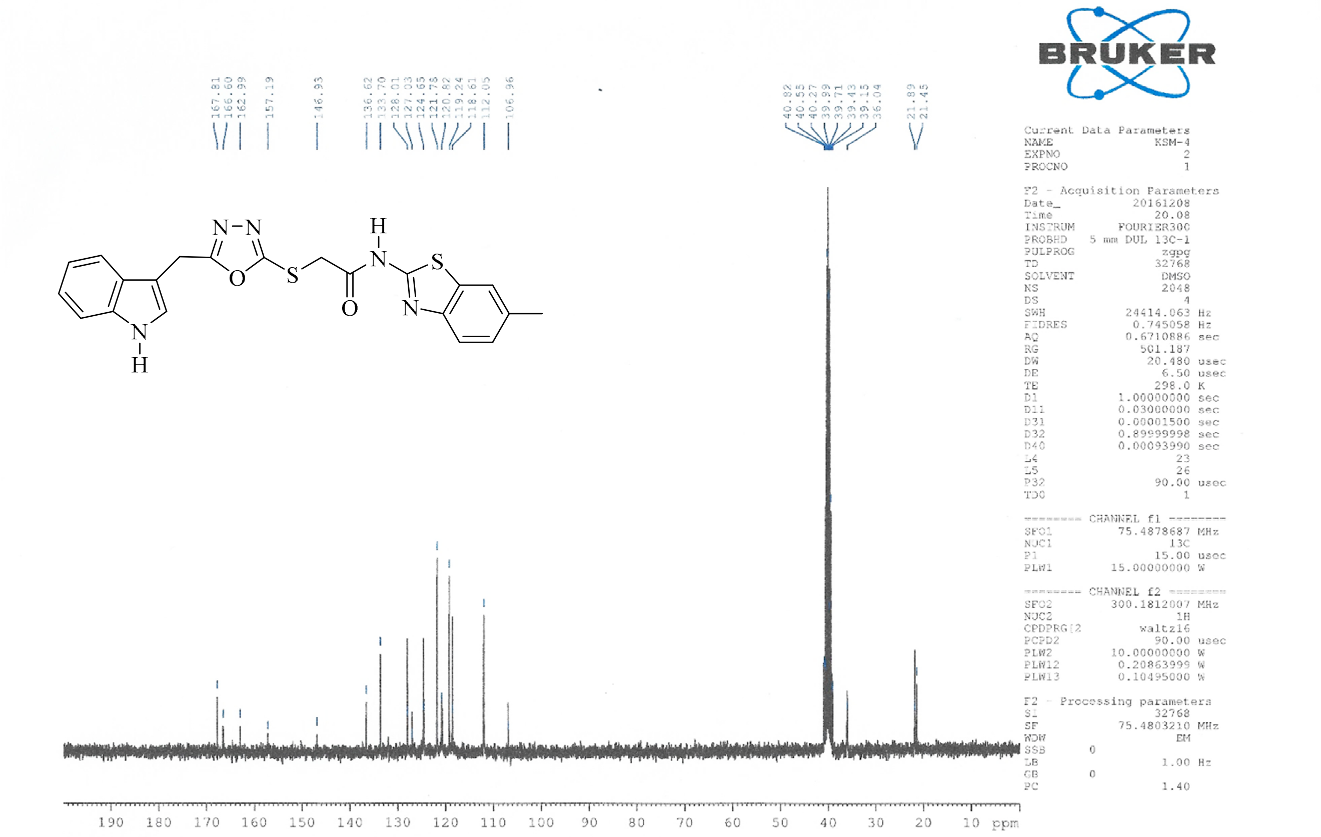

Figure S15. ^{13}C NMR spectrum of compound **2d.**

Formula Predictor Report - KSM-4_4.lcd

Page 1 of 1

Data File: C:\LabSolutions\Data\Analiz\mdaltintop\KSM-4_4.lcd

Elmt	Val.	Min	Max	Elmt	Val.	Min	Max	Elmt	Val.	Min	Max	Elmt	Val.	Min	Max	Use Adduct
H	1	10	20	O	2	2	4	Cl	1	0	1	I	3	0	0	H
C	4	18	30	F	1	0	0	Br	1	0	1					
N	3	5	6	S	2	2	2	Ru	2	0	0					

Error Margin (ppm):	5	DBE Range:	0.0 - 30.0	Electron Ions:	both
HC Ratio:	unlimited	Apply N Rule:	yes	Use MSn Info:	no
Max Isotopes:	3	Isotope RI (%):	1.00	Isotope Res:	10000
MSn Iso RI (%):	10.00	MSn Logic Mode:	AND	Max Results:	500

Event#: 1 MS(E+) Ret. Time : 6.573 -> 6.733 Scan# : 987 -> 1011

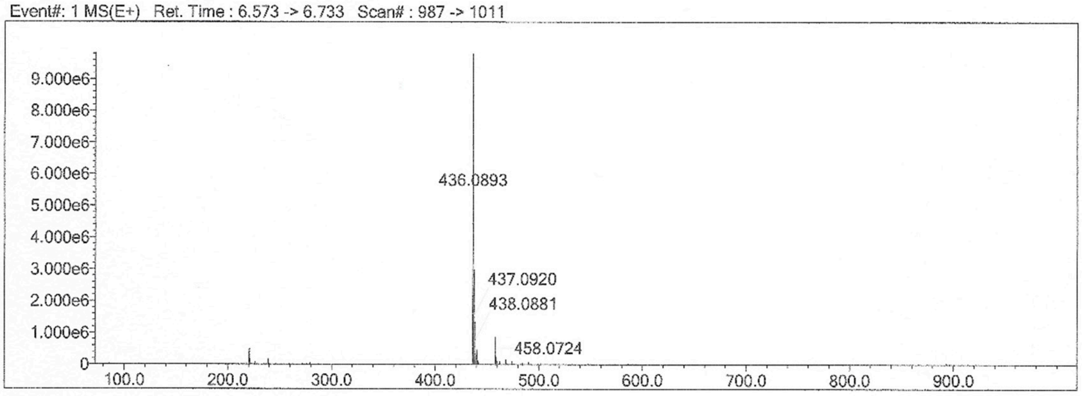

Measured region for 436.0893 m/z

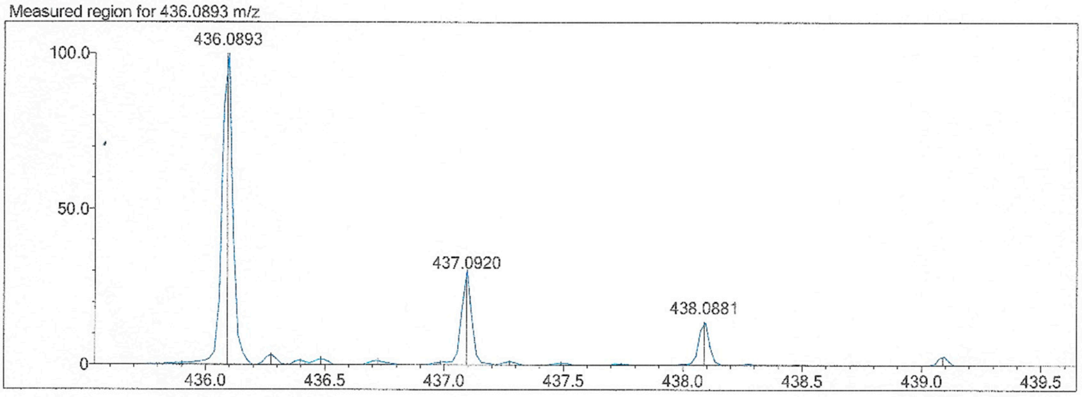

C21 H17 N5 O2 S2 [M+H]+ : Predicted region for 436.0896 m/z

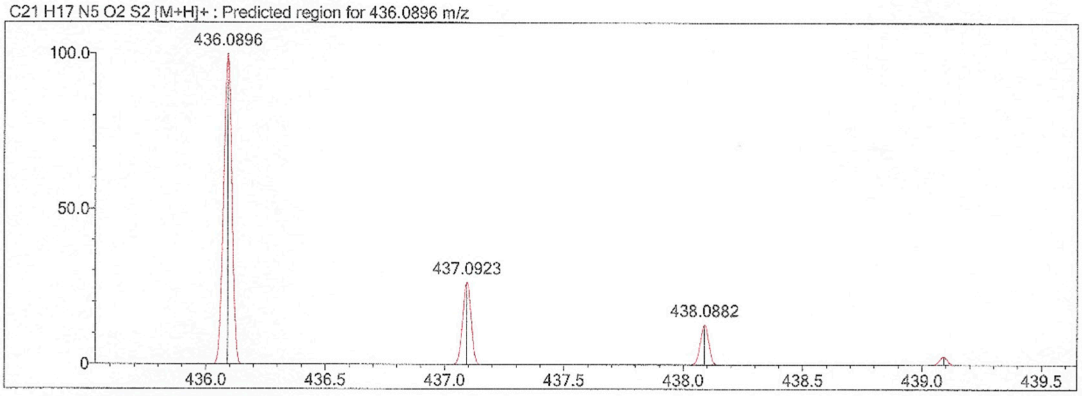

Rank	Score	Formula (M)	Ion	Meas. m/z	Pred. m/z	Df. (mDa)	Df. (ppm)	Iso	DBE
1	92.79	C21 H17 N5 O2 S2	[M+H]+	436.0893	436.0896	-0.3	-0.69	92.79	16.0

Figure S16. HRMS spectrum of compound **2d.**

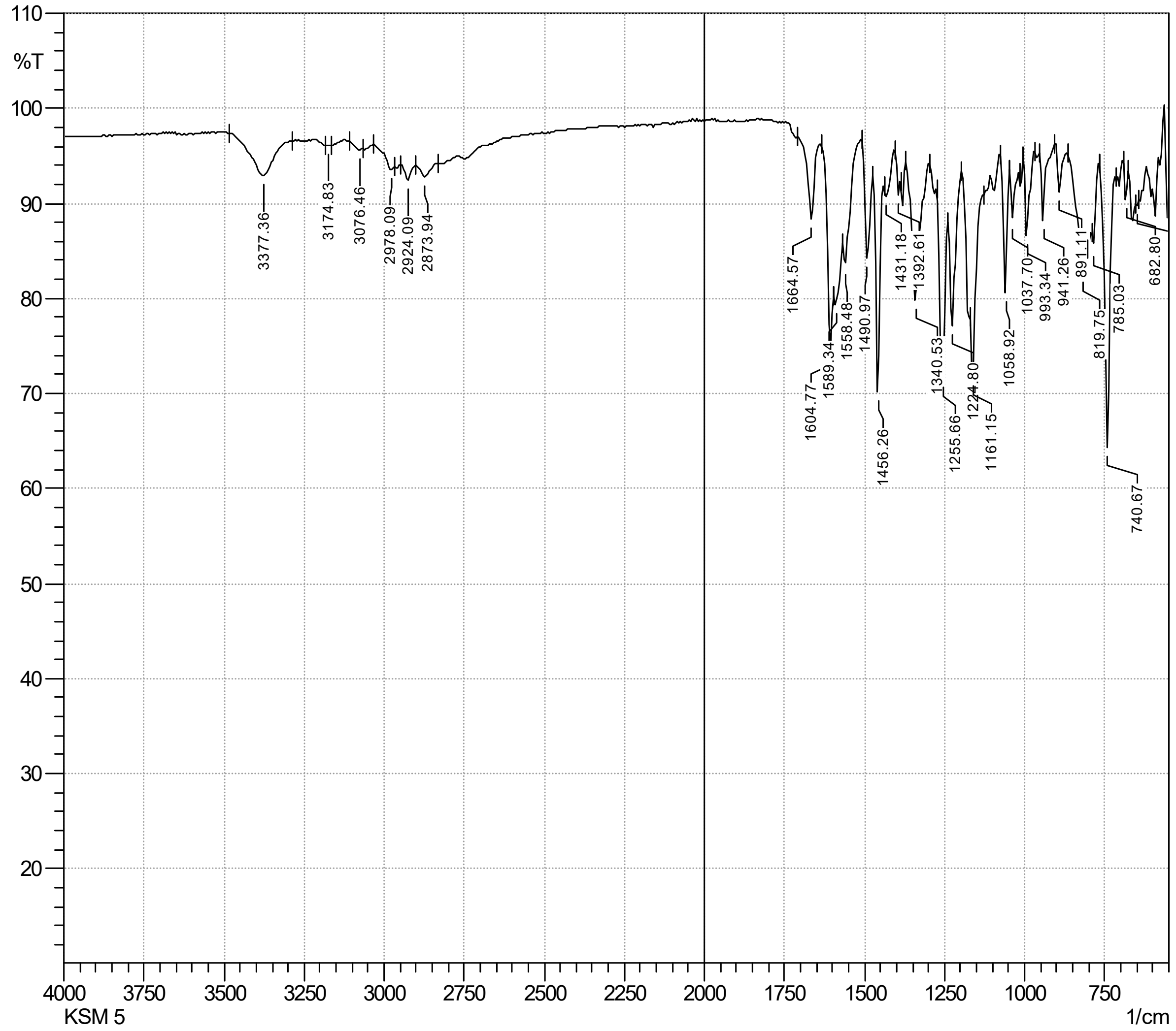

Figure S17. IR spectrum of compound **2e**.

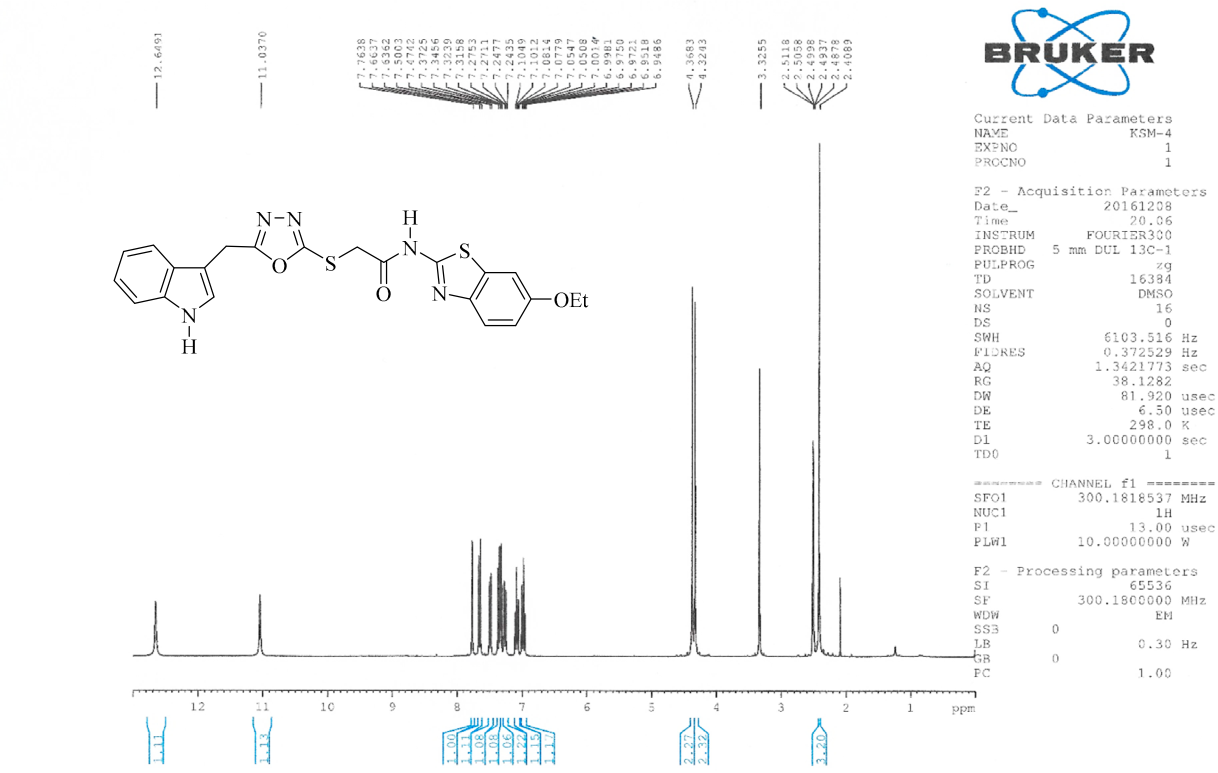

Figure S18. ^{1}H NMR spectrum of compound **2e**.

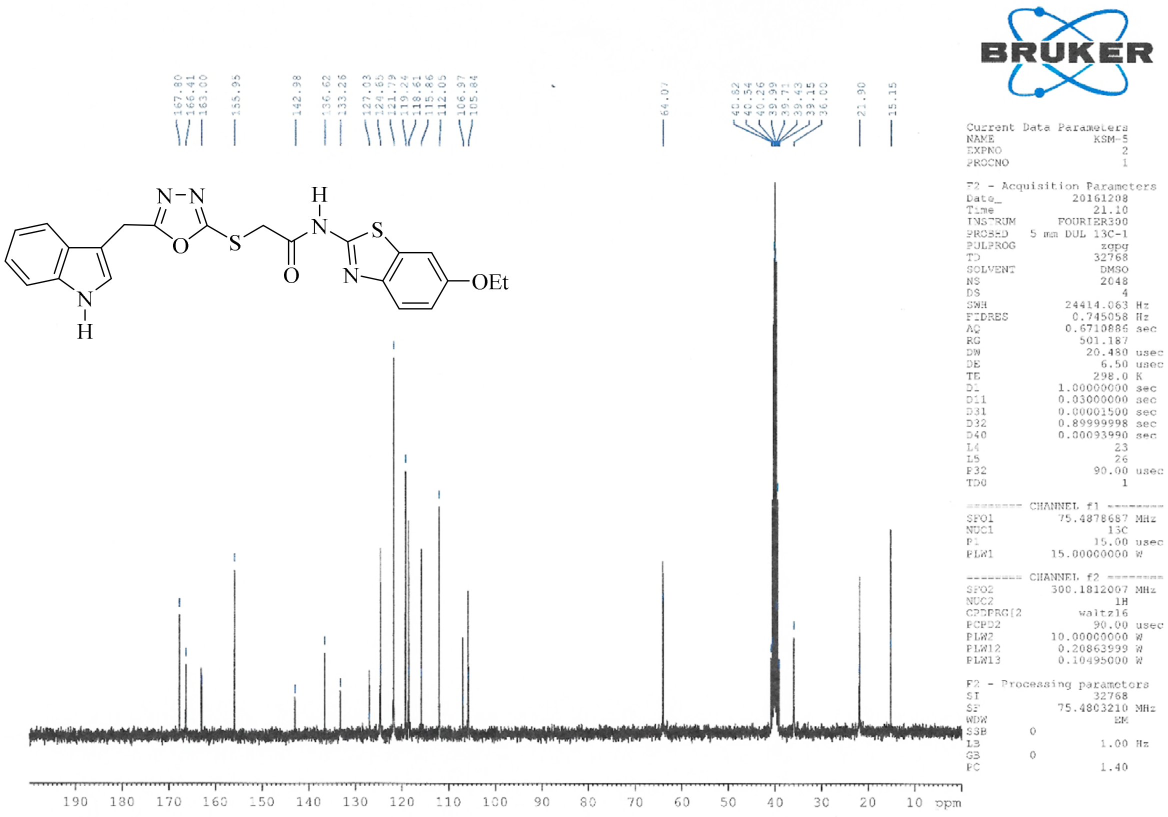

Figure S19. ^{13}C NMR spectrum of compound **2e**.

Formula Predictor Report - KSM-5_5.lcd

Page 1 of 1

Data File: C:\LabSolutions\Data\Analiz\mdaltintop\KSM-5_5.lcd

Elmt	Val.	Min	Max	Elmt	Val.	Min	Max	Elmt	Val.	Min	Max	Elmt	Val.	Min	Max	Use Adduct
H	1	10	20	O	2	2	4	Cl	1	0	1	I	3	0	0	H
C	4	18	30	F	1	0	0	Br	1	0	1					
N	3	5	6	S	2	2	2	Ru	2	0	0					

Error Margin (ppm): 5
HC Ratio: unlimited
Max Isotopes: 3
MSn Iso RI (%): 10.00

DBE Range: 0.0 - 30.0
Apply N Rule: yes
Isotope RI (%): 1.00
MSn Logic Mode: AND

Electron Ions: both
Use MSn Info: no
Isotope Res: 10000
Max Results: 500

Event#: 1 MS(E+) Ret. Time : 6.587 -> 6.707 Scan# : 989 -> 1007

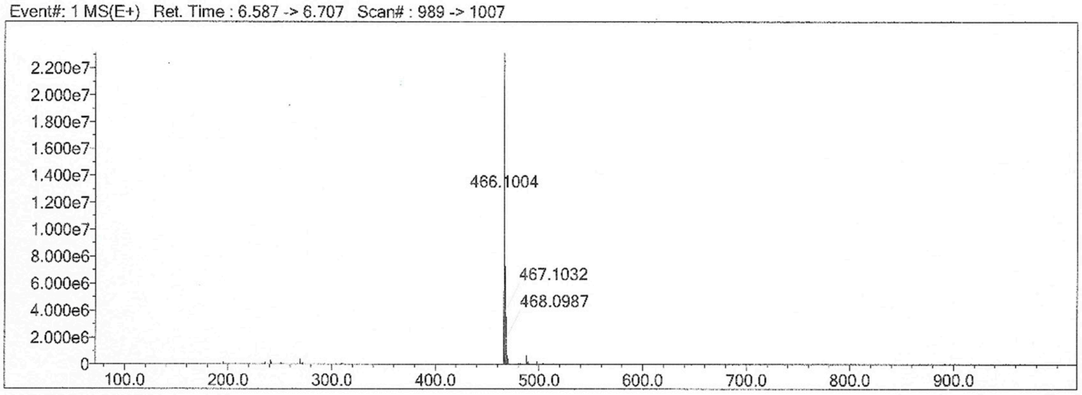

Measured region for 466.1004 m/z

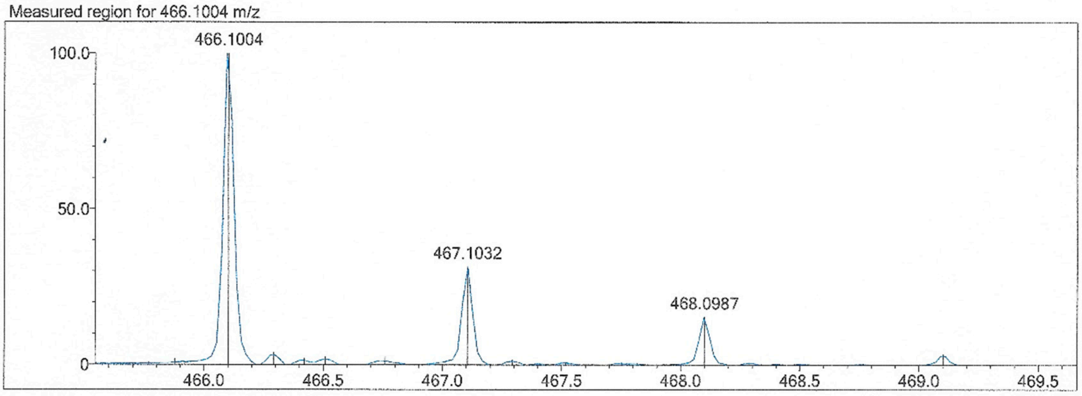

C22 H19 N5 O3 S2 [M+H]+ : Predicted region for 466.1002 m/z

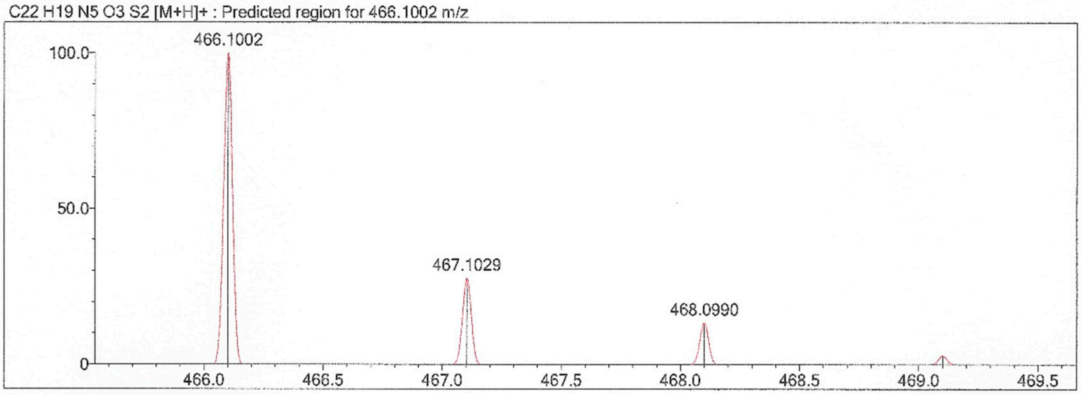

Rank	Score	Formula (M)	Ion	Meas. m/z	Pred. m/z	Df. (mDa)	Df. (ppm)	Iso	DBE
1	86.85	C22 H19 N5 O3 S2	[M+H]+	466.1004	466.1002	0.2	0.43	86.85	16.0

Figure S20. HRMS spectrum of compound **2e**.

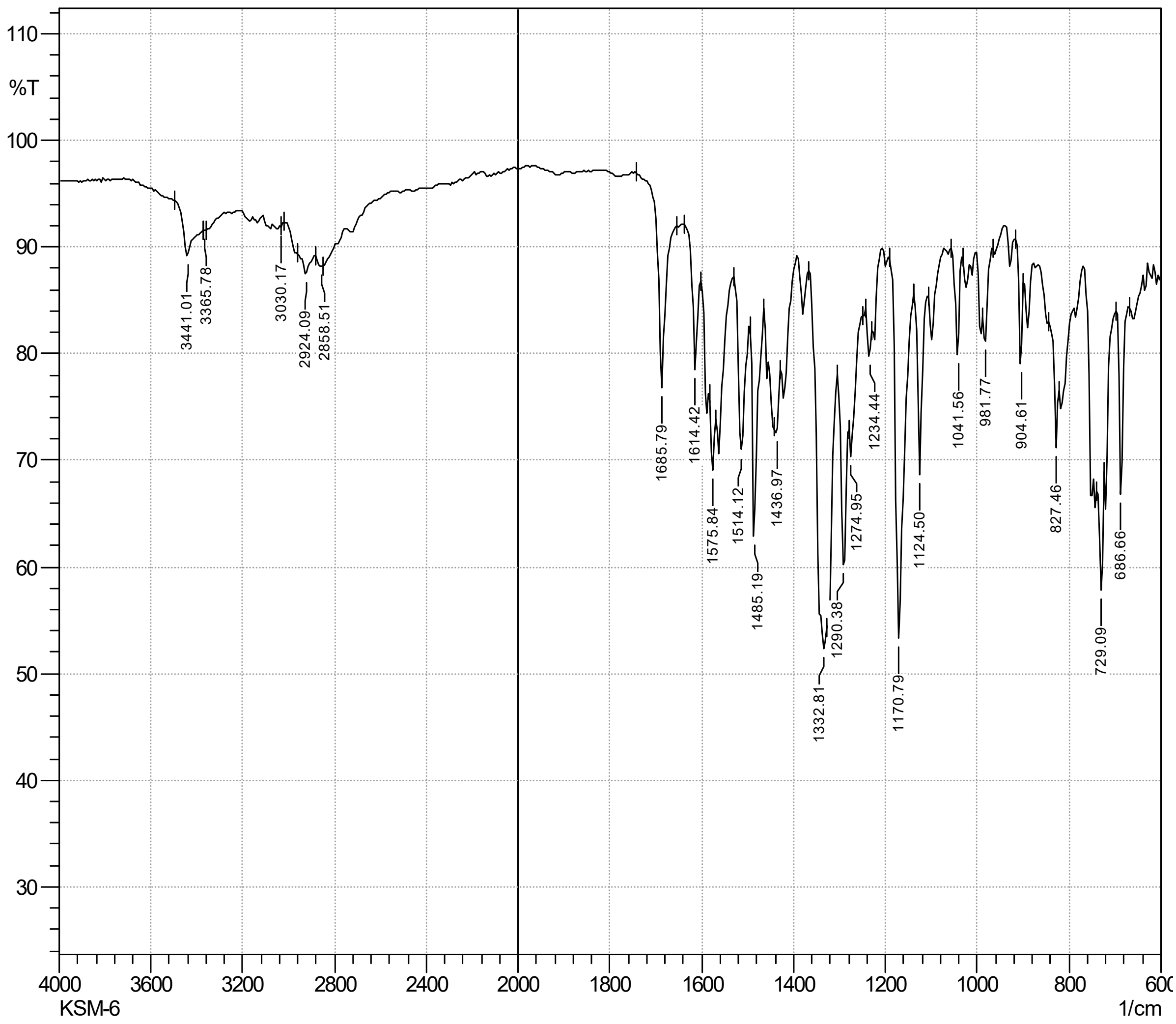

Figure S21. IR spectrum of compound **2f**.

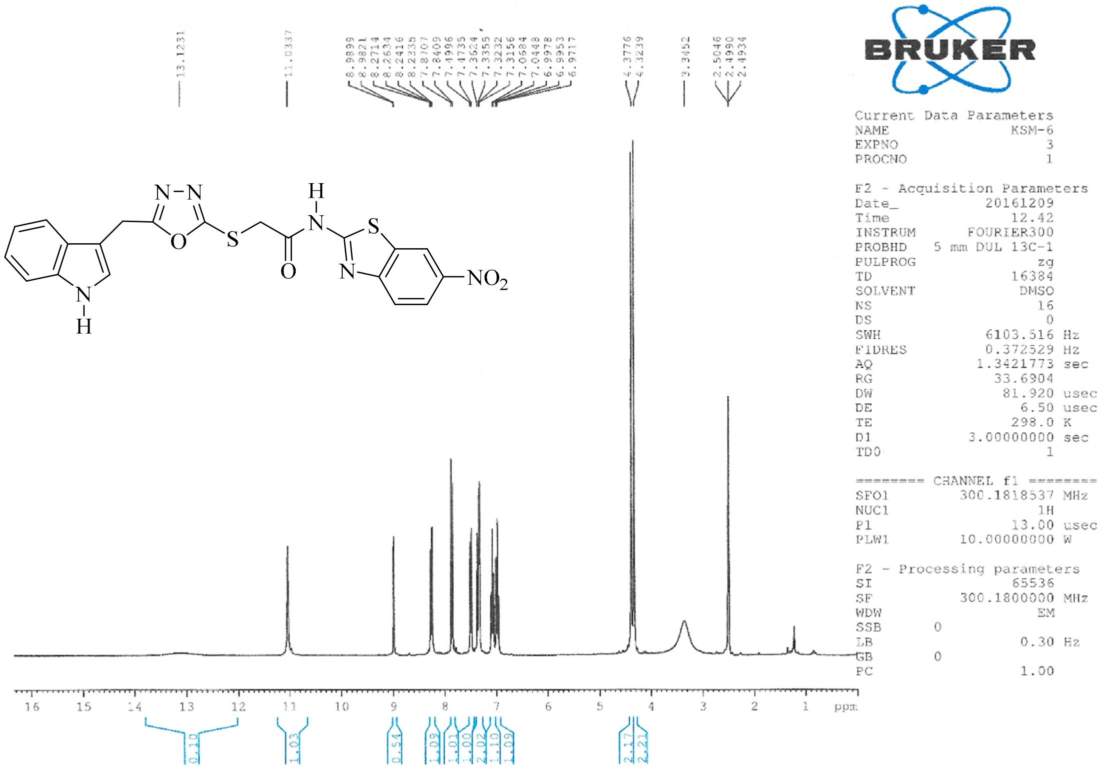

Figure S22. ^{1}H NMR spectrum of compound **2f**.

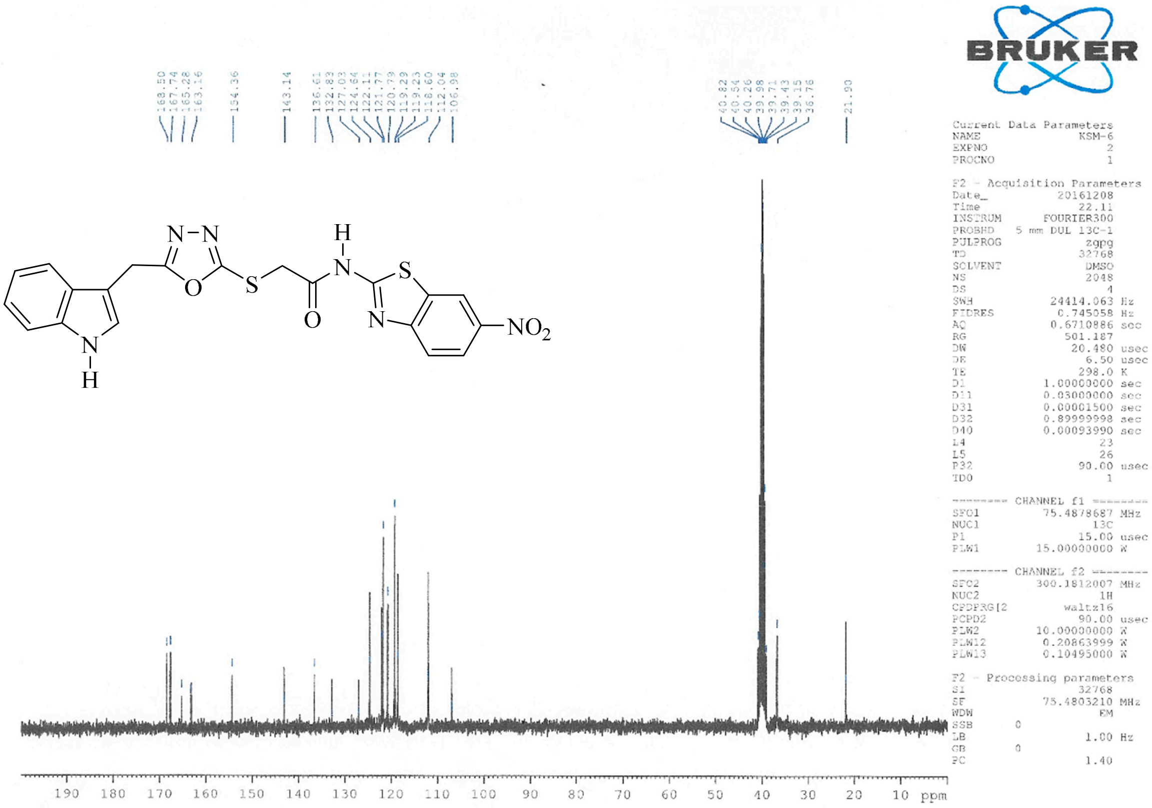

Figure S23. ^{13}C NMR spectrum of compound **2f**.

Formula Predictor Report - KSM-6_6.lcd

Page 1 of 1

Data File: C:\LabSolutions\Data\Analiz\mdaltintop\KSM-6_6.lcd

Elmt	Val.	Min	Max	Elmt	Val.	Min	Max	Elmt	Val.	Min	Max	Elmt	Val.	Min	Max	Use Adduct
H	1	10	20	O	2	2	4	Cl	1	0	1	I	3	0	0	H
C	4	18	30	F	1	0	0	Br	1	0	1					
N	3	5	6	S	2	2	2	Ru	2	0	0					

Error Margin (ppm):	5	DBE Range:	0.0 - 30.0	Electron Ions:	both
HC Ratio:	unlimited	Apply N Rule:	yes	Use MSn Info:	no
Max Isotopes:	3	Isotope RI (%):	1.00	Isotope Res:	10000
MSn Iso RI (%):	10.00	MSn Logic Mode:	AND	Max Results:	500

Event#: 1 MS(E+) Ret. Time : 6.400 -> 6.520 Scan# : 961 -> 979

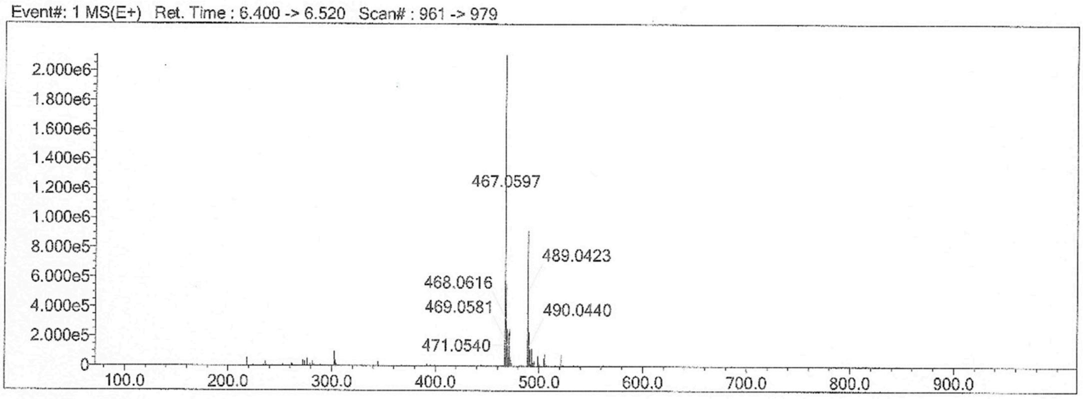

Measured region for 467.0597 m/z

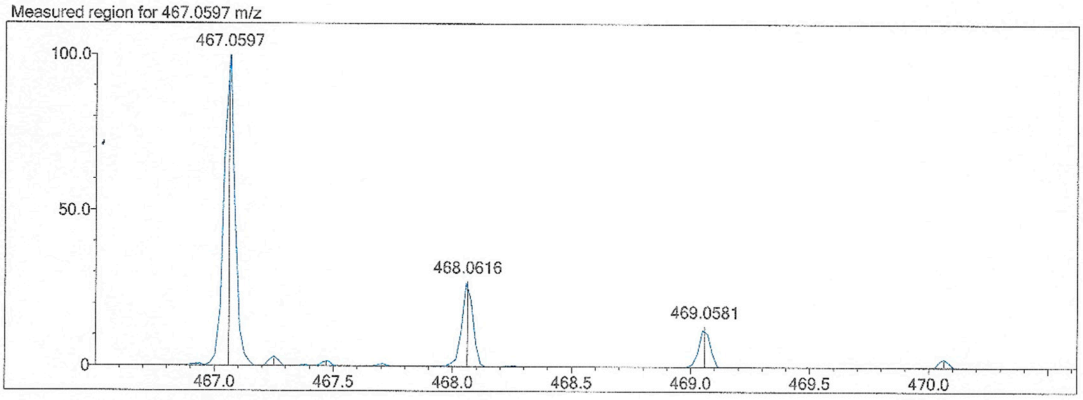

C20 H14 N6 O4 S2 [M+H]+ : Predicted region for 467.0591 m/z

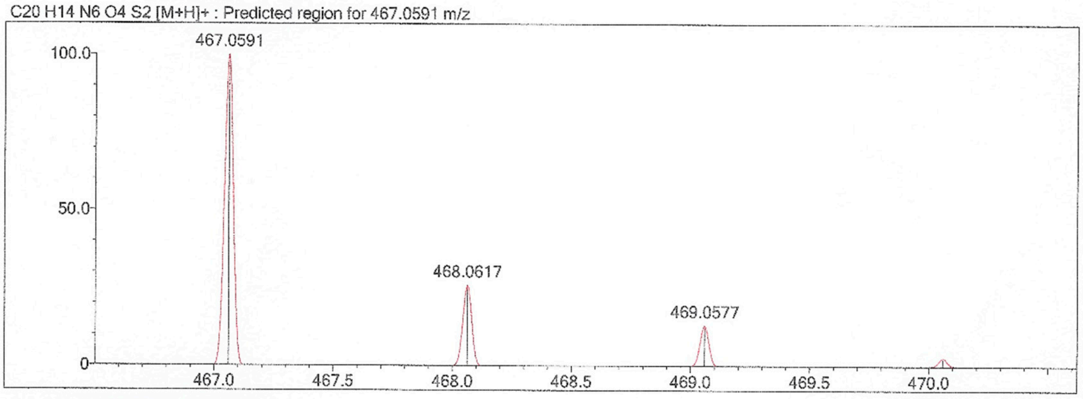

Rank	Score	Formula (M)	Ion	Meas. m/z	Pred. m/z	Df. (mDa)	Df. (ppm)	Iso	DBE
1	92.22	C20 H14 N6 O4 S2	[M+H]+	467.0597	467.0591	0.6	1.28	92.87	17.0

Figure S24. HRMS spectrum of compound **2f**.

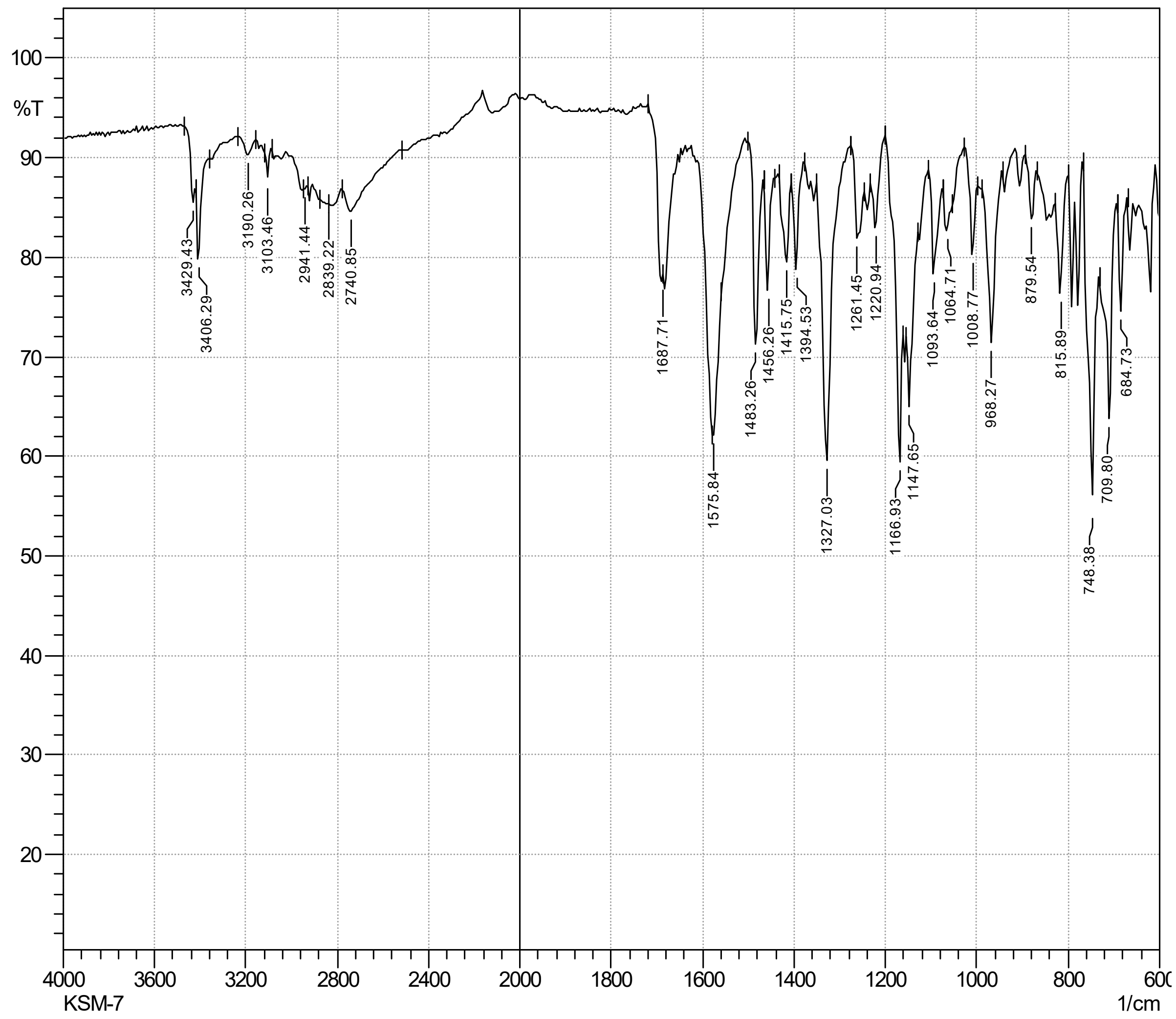

Figure S25. IR spectrum of compound **2g**.

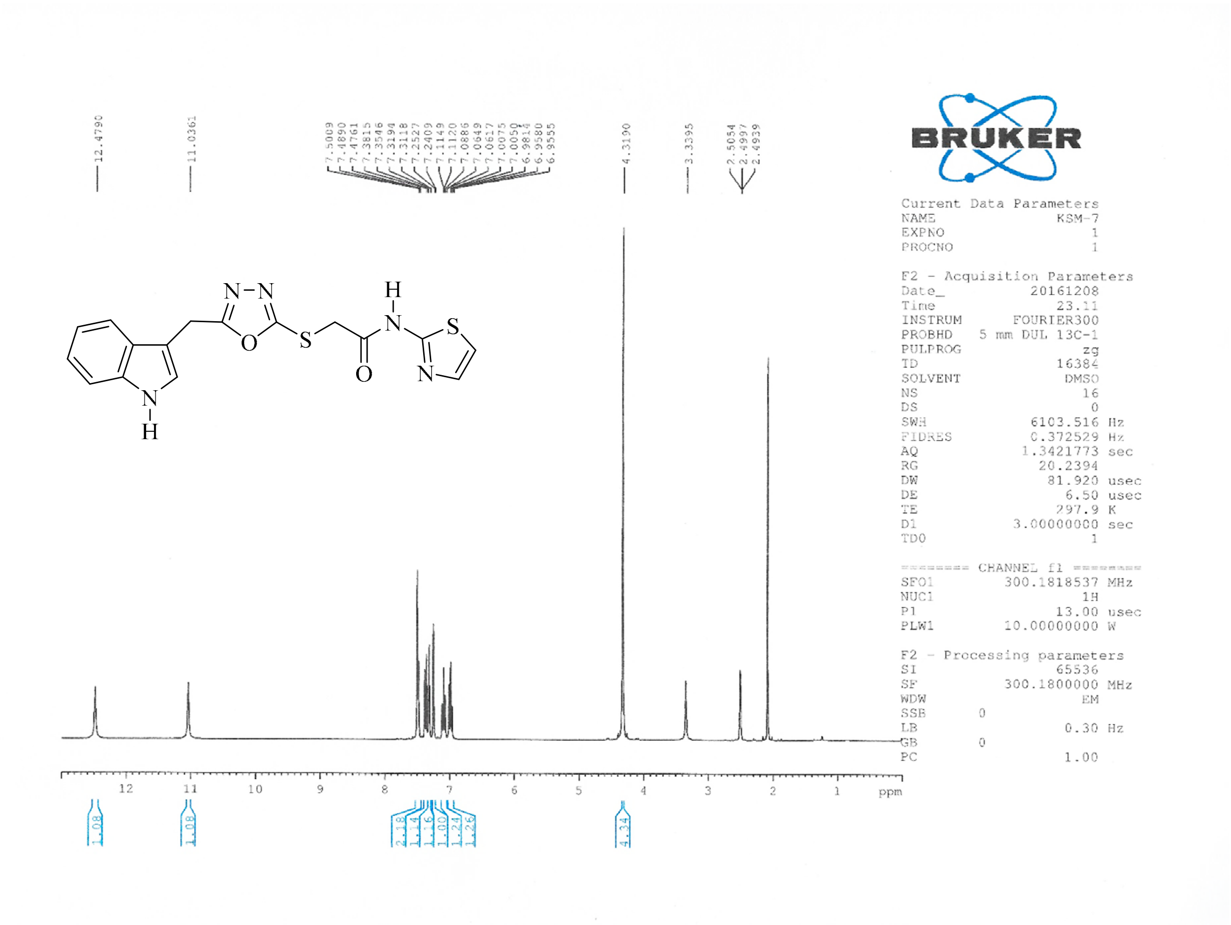

Figure S26. ^{1}H NMR spectrum of compound **2g**.

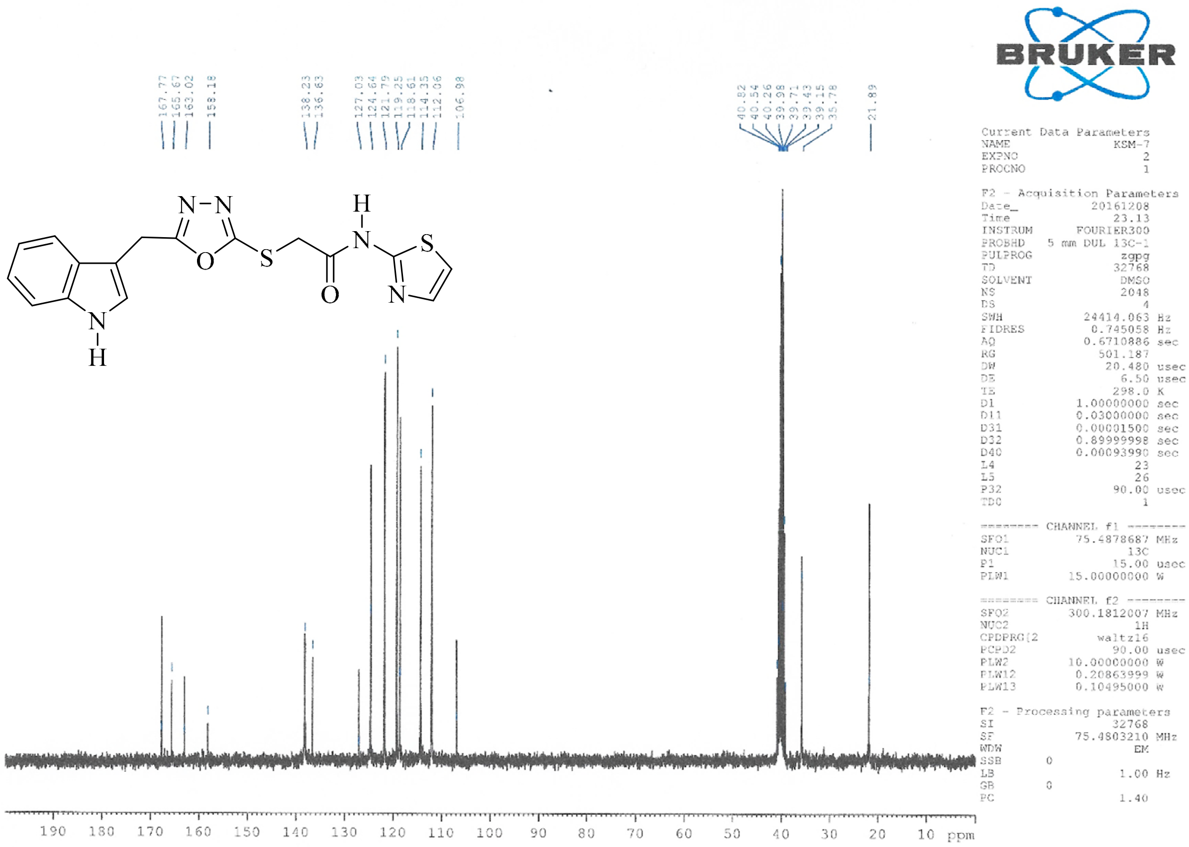

Figure S27. ^{13}C NMR spectrum of compound **2g**.

Formula Predictor Report - KSM-7_7.lcd

Page 1 of 1

Data File: C:\LabSolutions\Data\Analiz\mdaltintop\KSM-7_7.lcd

Elmt	Val.	Min	Max	Elmt	Val.	Min	Max	Elmt	Val.	Min	Max	Elmt	Val.	Min	Max	Use Adduct
H	1	10	20	O	2	2	4	Cl	1	0	1	I	3	0	0	H
C	4	15	30	F	1	0	0	Br	1	0	1					
N	3	5	6	S	2	2	2	Ru	2	0	0					

Error Margin (ppm): 5
HC Ratio: unlimited
Max Isotopes: 3
MSn Iso RI (%): 10.00

DBE Range: 0.0 - 30.0
Apply N Rule: yes
Isotope RI (%): 1.00
MSn Logic Mode: AND

Electron Ions: both
Use MSn Info: no
Isotope Res: 10000
Max Results: 500

Event#: 1 MS(E+) Ret. Time : 5.520 -> 5.613 Scan# : 829 -> 843

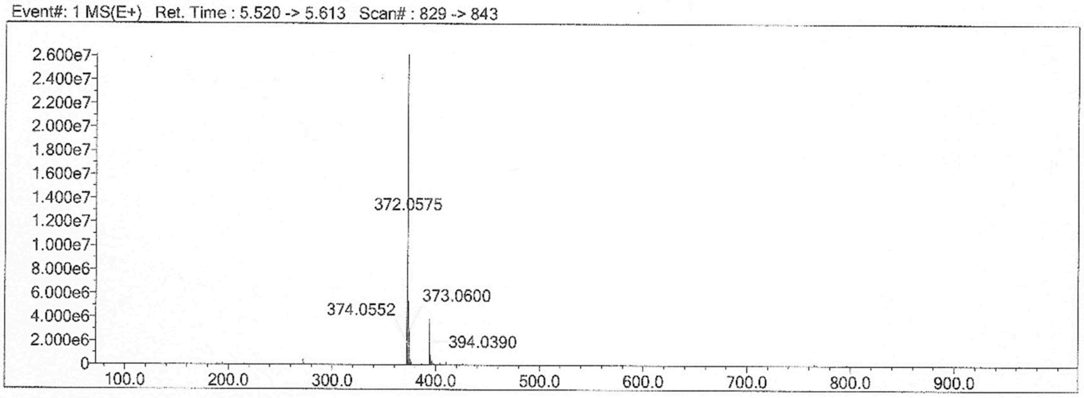

Measured region for 372.0575 m/z

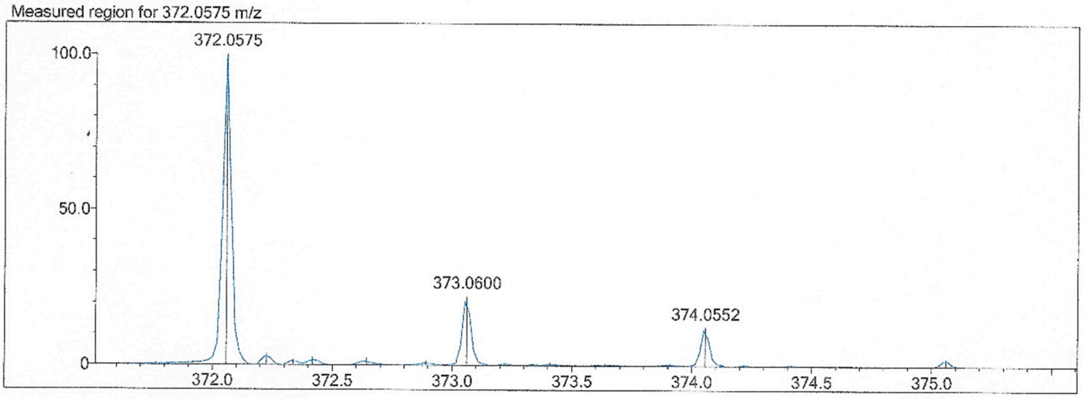

C16 H13 N5 O2 S2 [M+H]+ : Predicted region for 372.0583 m/z

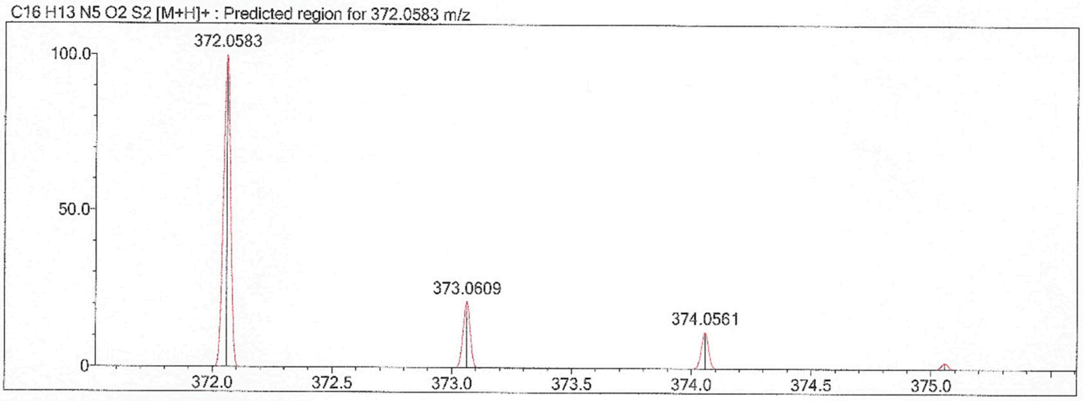

Rank	Score	Formula (M)	Ion	Meas. m/z	Pred. m/z	Df. (mDa)	Df. (ppm)	Iso	DBE
1	87.85	C16 H13 N5 O2 S2	[M+H]+	372.0575	372.0583	-0.8	-2.15	90.45	13.0

Figure S28. HRMS spectrum of compound **2g.**

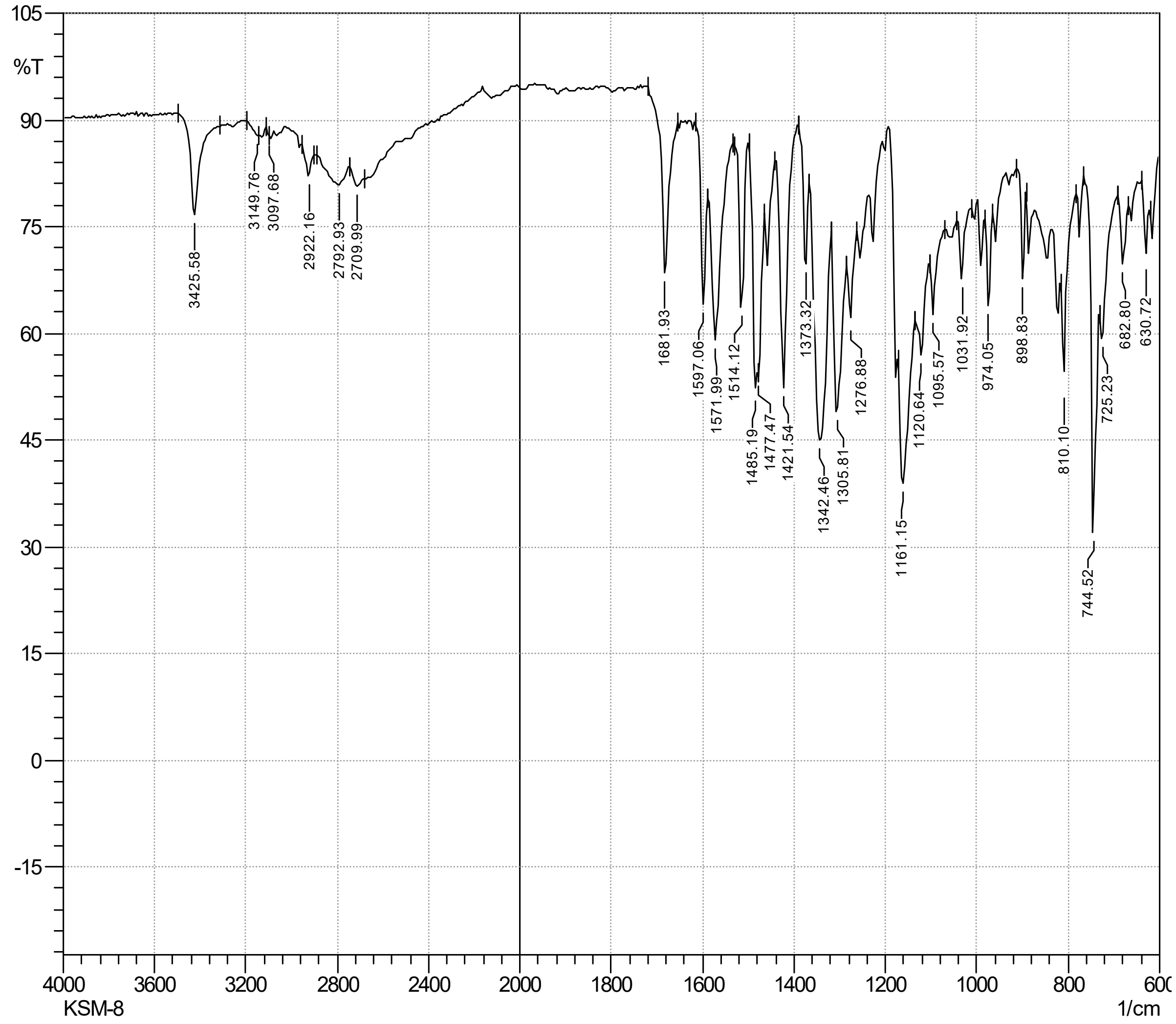

Figure S29. IR spectrum of compound **2h**.

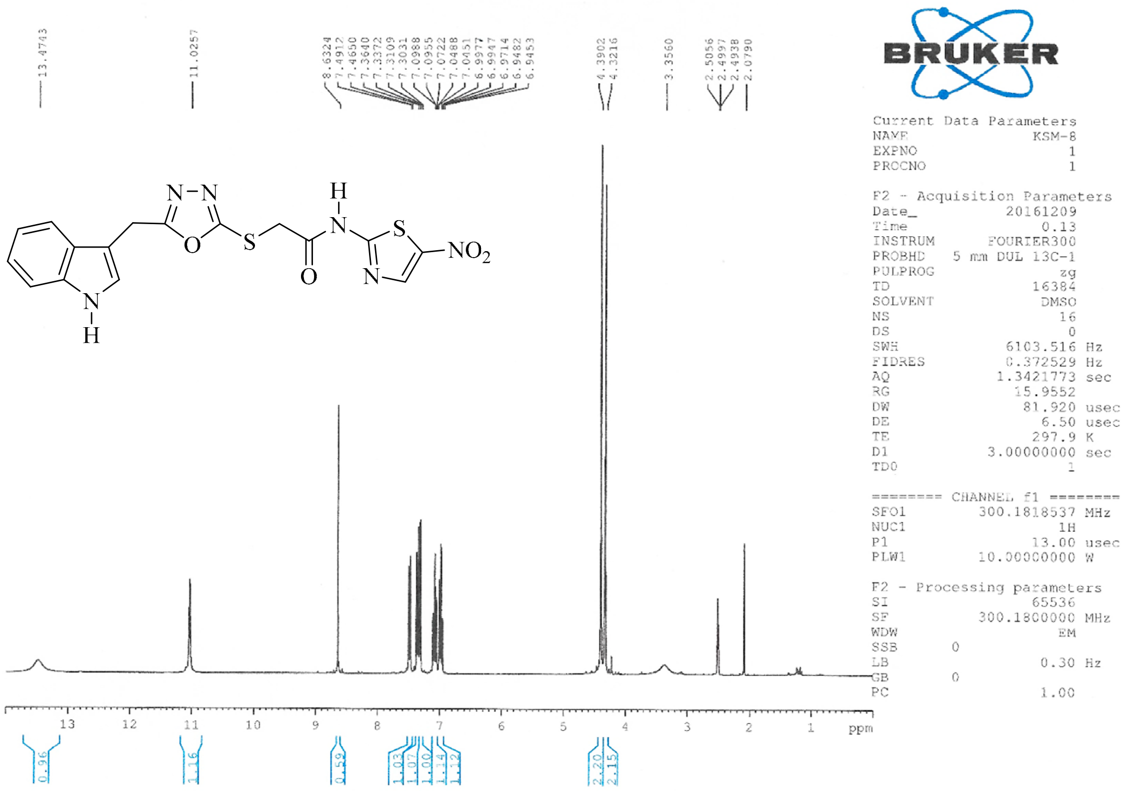

Figure S30. ^{1}H NMR spectrum of compound **2h.**

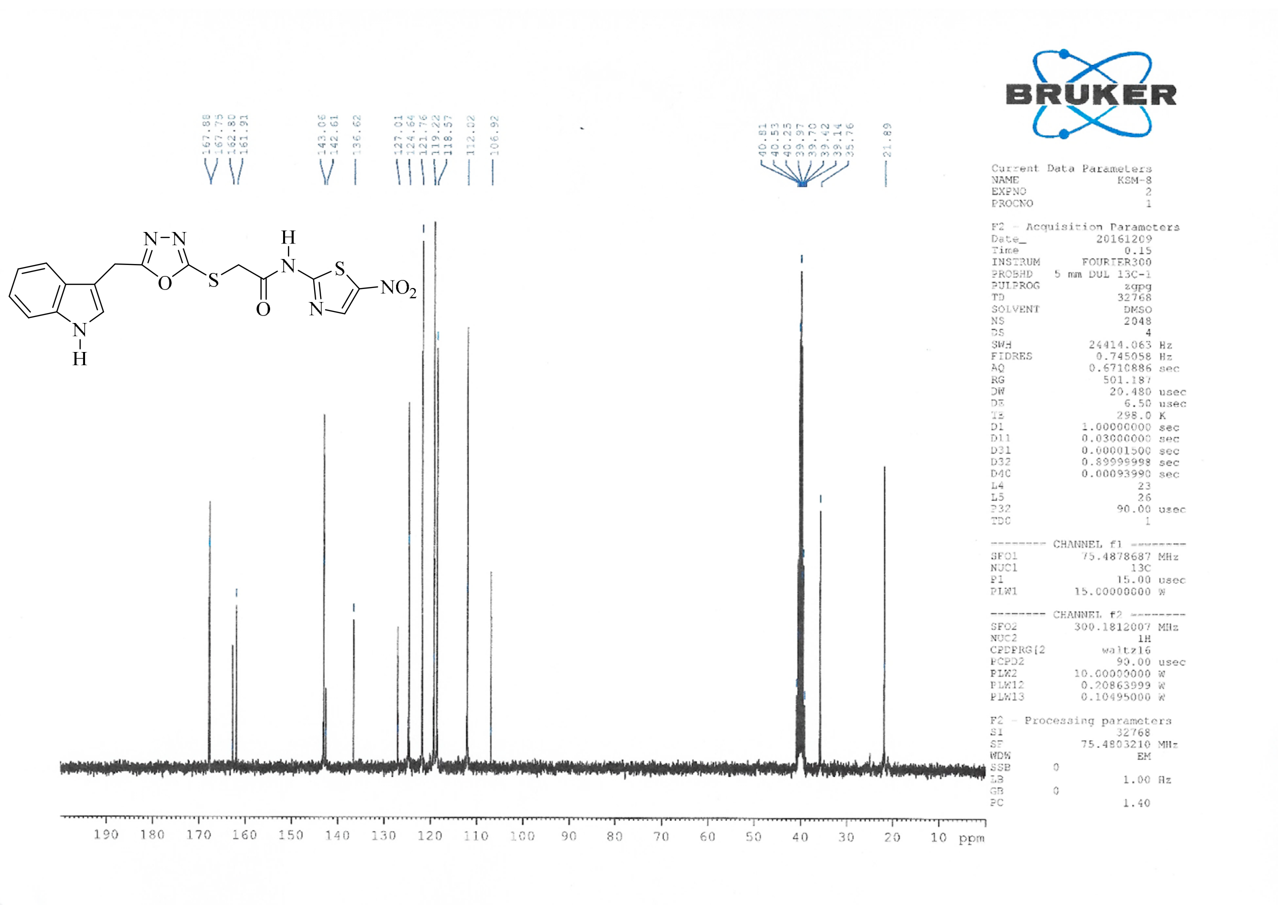

Figure S31. ^{13}C NMR spectrum of compound **2h**.

Formula Predictor Report - KSM-8_8.lcd

Data File: C:\LabSolutions\Data\Analiz\mdaltintop\KSM-8_8.lcd

Elmt	Val.	Min	Max	Elmt	Val.	Min	Max	Elmt	Val.	Min	Max	Elmt	Val.	Min	Max	Use Adduct
H	1	10	20	O	2	2	4	Cl	1	0	1	I	3	0	0	H
C	4	15	30	F	1	0	0	Br	1	0	1					
N	3	5	6	S	2	2	2	Ru	2	0	0					

Error Margin (ppm):	5	DBE Range:	0.0 - 30.0	Electron Ions:	both
HC Ratio:	unlimited	Apply N Rule:	yes	Use MSn Info:	no
Max Isotopes:	3	Isotope RI (%):	1.00	Isotope Res:	10000
MSn Iso RI (%):	10.00	MSn Logic Mode:	AND	Max Results:	500

Event#: 1 MS(E+) Ret. Time : 5.973 -> 6.160 Scan# : 897 -> 925

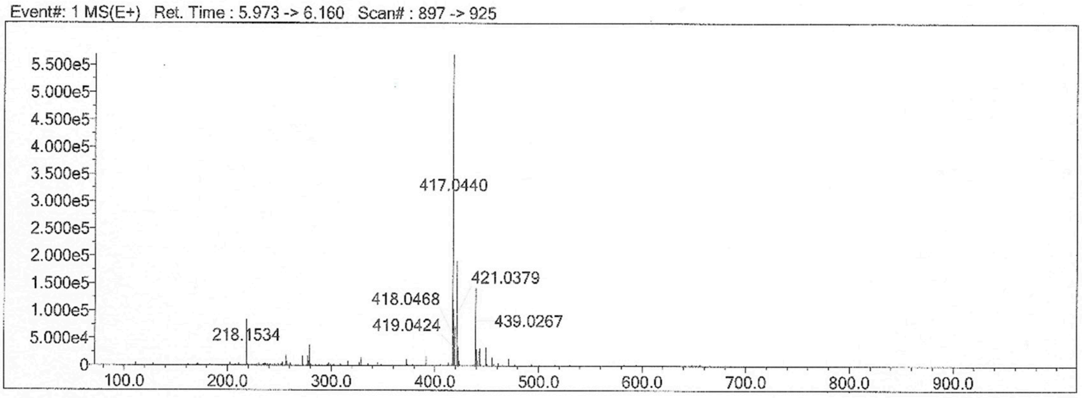

Measured region for 417.0440 m/z

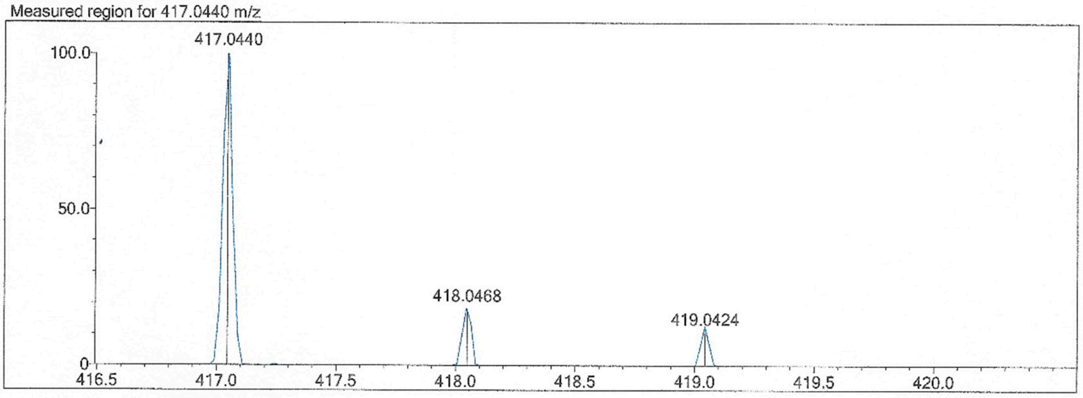

C16 H12 N6 O4 S2 [M+H]+ : Predicted region for 417.0434 m/z

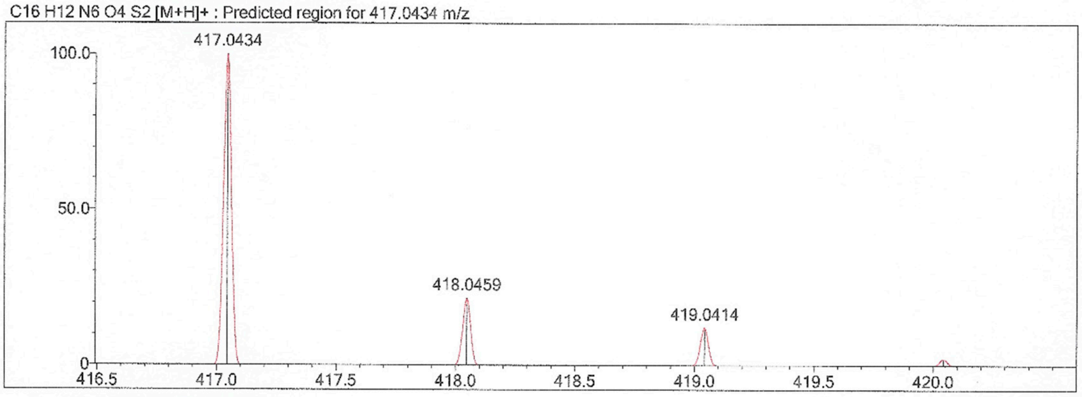

Rank	Score	Formula (M)	Ion	Meas. m/z	Pred. m/z	Df. (mDa)	Df. (ppm)	Iso	DBE
1	77.58	C16 H12 N6 O4 S2	[M+H]+	417.0440	417.0434	0.6	1.44	78.45	14.0

Figure S32. HRMS spectrum of compound **2h.**

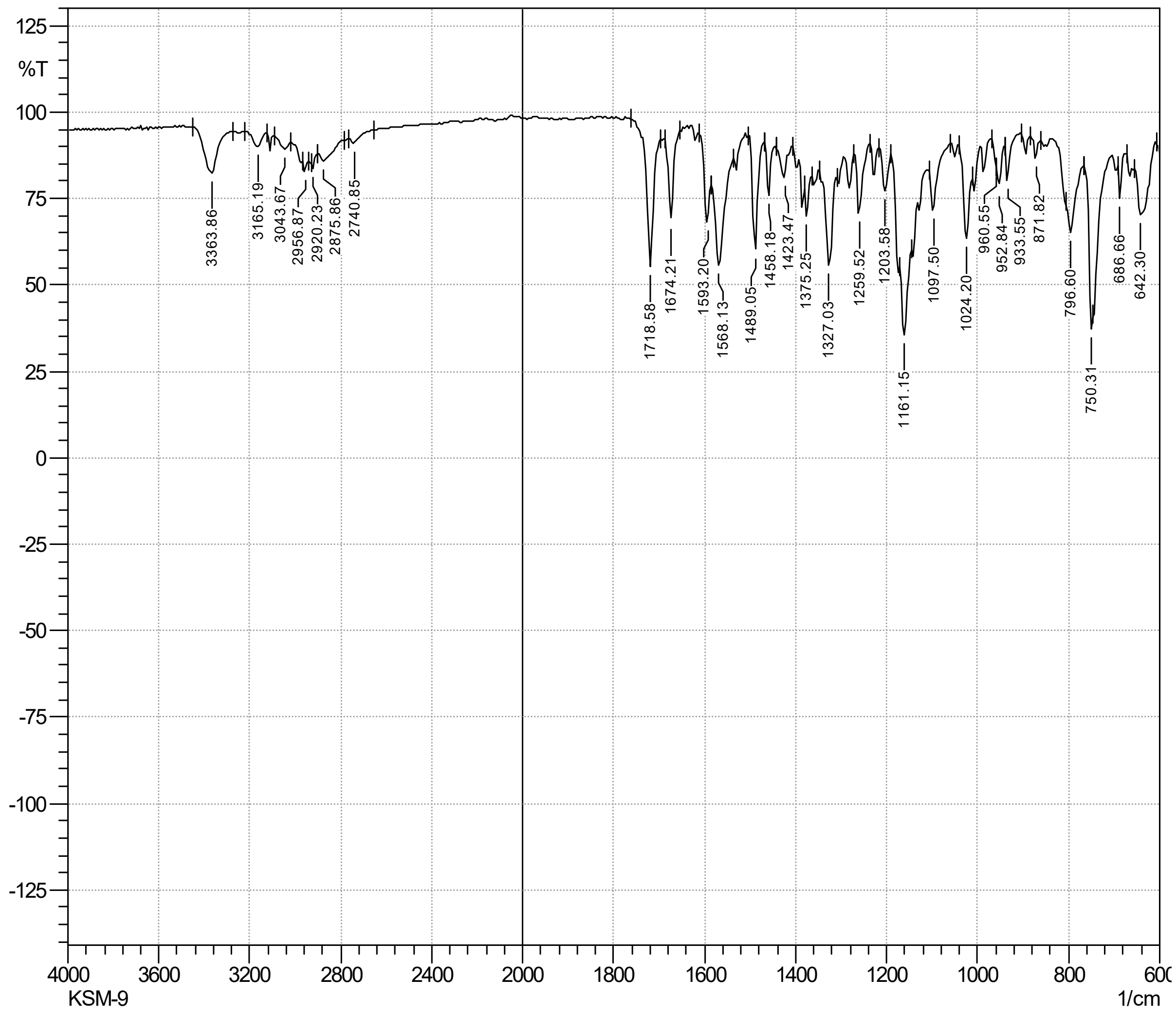

Figure S33. IR spectrum of compound **2i**.

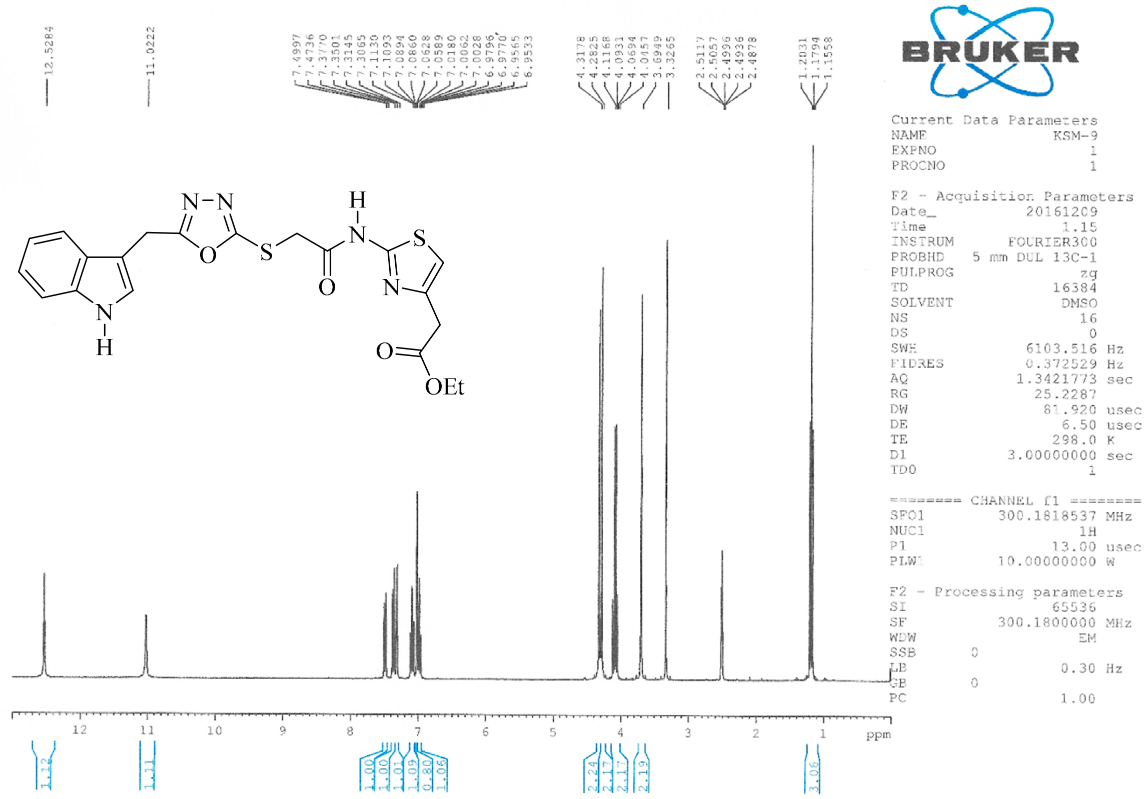

Figure S34. ^{1}H NMR spectrum of compound **2i**.

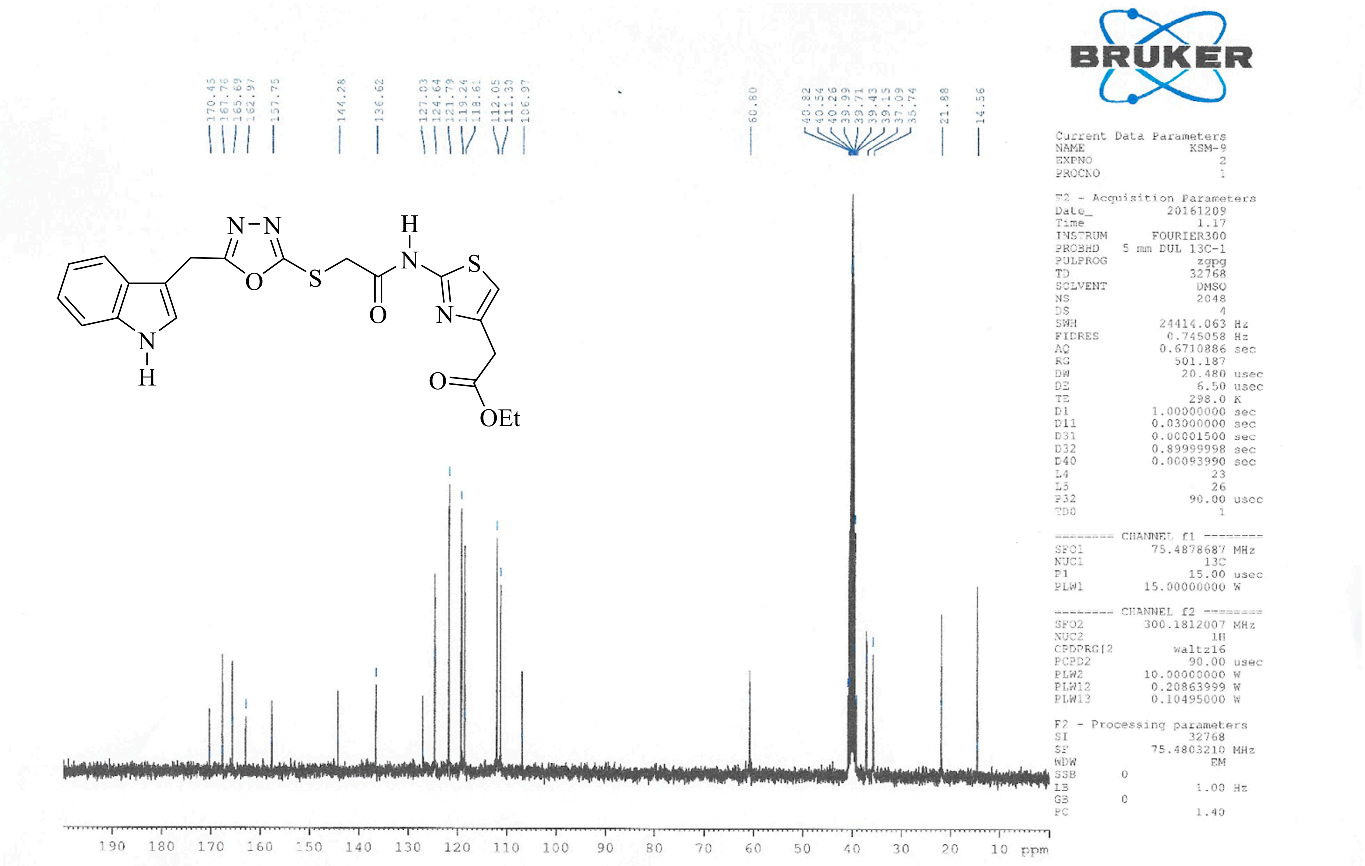

Figure S35. ^{13}C NMR spectrum of compound **2i**.

Formula Predictor Report - KSM-9_9.lcd Page 1 of 1

Data File: C:\LabSolutions\Data\Analiz\mdaltintop\KSM-9_9.lcd

Elmt	Val.	Min	Max	Elmt	Val.	Min	Max	Elmt	Val.	Min	Max	Elmt	Val.	Min	Max	Use Adduct
H	1	10	20	O	2	2	4	Cl	1	0	1	I	3	0	0	H
C	4	15	30	F	1	0	0	Br	1	0	1					
N	3	5	6	S	2	2	2	Ru	2	0	0					

Error Margin (ppm):	5	DBE Range:	0.0 - 30.0	Electron Ions:	both
HC Ratio:	unlimited	Apply N Rule:	yes	Use MSn Info:	no
Max Isotopes:	3	Isotope RI (%):	1.00	Isotope Res:	10000
MSn Iso RI (%):	10.00	MSn Logic Mode:	AND	Max Results:	500

Event#: 1 MS(E+) Ret. Time : 5.960 -> 6.067 Scan# : 895 -> 911

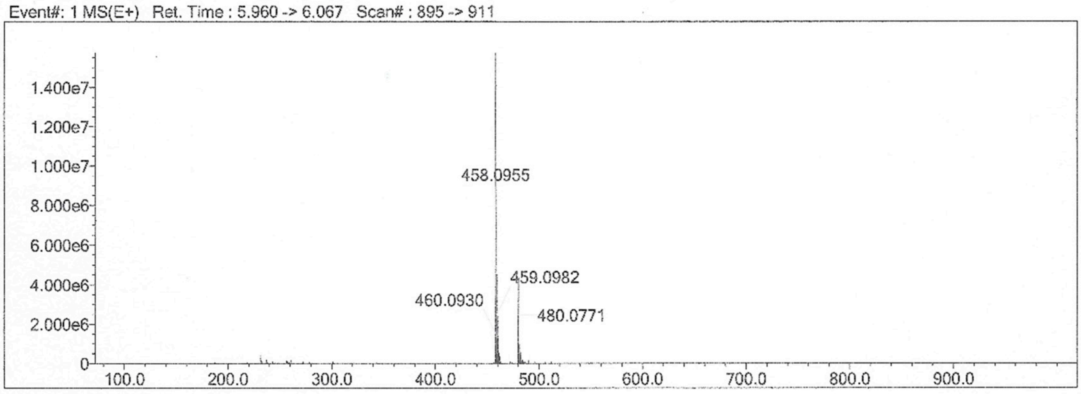

Measured region for 458.0955 m/z

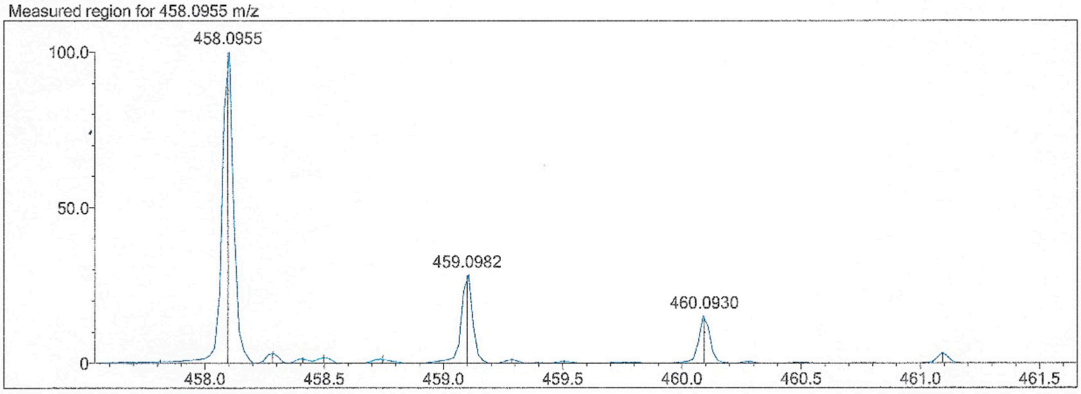

C20 H19 N5 O4 S2 [M+H]+ : Predicted region for 458.0951 m/z

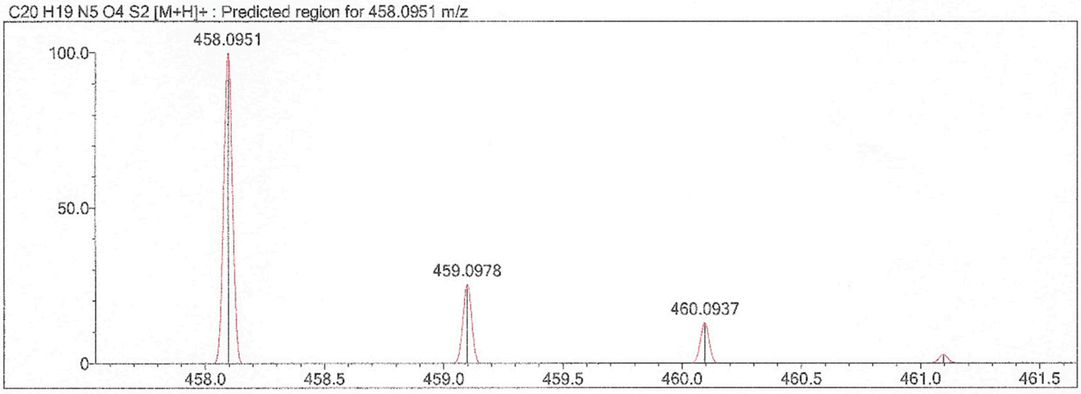

Rank	Score	Formula (M)	Ion	Meas. m/z	Pred. m/z	Df. (mDa)	Df. (ppm)	Iso	DBE
1	93.24	C20 H19 N5 O4 S2	[M+H]+	458.0955	458.0951	0.4	0.87	93.24	14.0

Figure S36. HRMS spectrum of compound **2i**.
